# Interactions between Nitric Oxide and Hyaluronan Implicate the Migration of Breast Cancer Cells

**DOI:** 10.1021/acs.biomac.2c00545

**Published:** 2022-08-03

**Authors:** Amir M. Alsharabasy, Sharon Glynn, Pau Farràs, Abhay Pandit

**Affiliations:** † CÚRAM, SFI Research Centre for Medical Devices, National University of Ireland Galway, Galway H91 W2TY, Ireland; ‡ Discipline of Pathology, Lambe Institute for Translational Research, School of Medicine, National University of Ireland Galway, Galway H91 TK33, Ireland; § School of Biological and Chemical Sciences, Ryan Institute, National University of Ireland Galway, Galway H91 TK33, Ireland

## Abstract

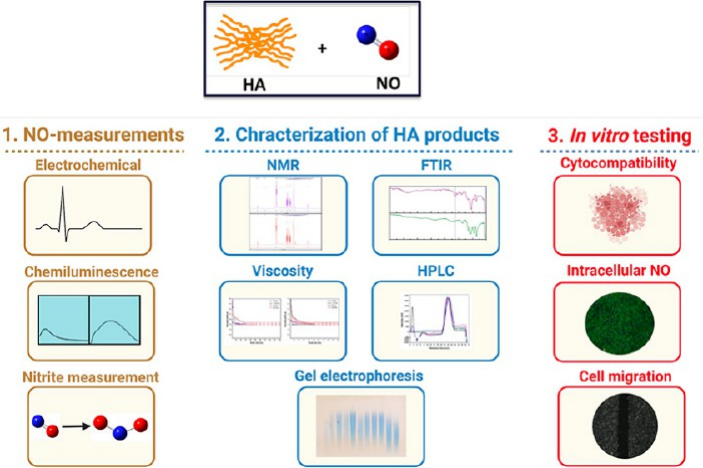

Nitric oxide (^•^NO) is one of the prominent free radicals, playing a pivotal role in breast cancer progression. Hyaluronic acid (HA) plays an essential role in neutralizing free radicals in tumor tissues. However, its interactions with nitric oxide have not been thoroughly investigated. Hence, this study attempts to understand the mechanism of these interactions and the different effects on the intracellular ^•^NO levels and migration of breast cancer cells. The affinity of HA to scavenge ^•^NO was investigated alongside the accompanying changes in specific physico-chemical properties and the further effects on the ^•^NO-induced attachment and migration of the breast cancer cell lines, MDA-MB-231 and HCC1806. The reaction of the nitrogen dioxide radical, formed via ^•^NO/O_2_ interactions, with HA initiated a series of oxidative reactions, which, in the presence of ^•^NO, induce the fragmentation of the polymeric chains. Furthermore, these interactions were found to hinder the NO-induced migration of cancer cells. However, the NO-induced HA modification/fragmentation was inhibited in the presence of hemin, a NO-scavenging compound. Collectively, these results help toward understanding the involvement of HA in the ^•^NO-induced cell migration and suggest the possible modification of HA, used as one of the main materials in different biomedical applications.

## 1 Introduction

Hyaluronan is a non-sulfated glycosaminoglycan composed of d-glucuronic acid and β-*N*-acetylglucosamine (GlcNAc), linked via alternating β-(1,4) and β-(1,3) glycosidic bonds, and distributed within the extracellular matrix (ECM) of various tissues.^1^ The polymer is synthesized by the action of the plasma-associated enzymes, hyaluronic acid (HA) synthases, and then extruded into the ECM.^2^ It has been reported that high levels of HA can be employed as a potential prognostic biomarker for breast cancer.^3,4^ Triple-negative breast cancer is one of the highly aggressive types of breast cancers with a poor prognosis.^5^ Moreover, the accumulation of HA creates gel-filled spaces in the ECM, guided by its high efficiency to form highly hydrated coils.^6^ The enhanced synthesis of HA by cancer cells forms a less dense matrix, responsible for the facilitation of tumor cell detachment and enhanced migration.^6,7^ These effects are also mediated by the various interactions between HA and the different cell surface receptors, including CD44 and the receptor of HA-mediated motility (RHAMM), where HA can modulate the growth, differentiation, migration, and invasion of the tumor cells both in vitro and in vivo.^8,9^ Hence, HA has been employed to fabricate multifunctional drug delivery systems and therapeutic devices.^10−12^ Moreover, HA-based three-dimensional in vitro cell culture models were developed for drug screening^13,14^ and studying the effects of matrix stiffness and its mechanical properties on cell functions.^15,16^

The reactive oxygen and nitrogen species are pro-oxidant molecules usually produced via various aerobic metabolic reactions, with essential roles in multiple physiological processes.^17−19^ However, under certain pathological conditions, their production exceeds the ability of their suppressing machinery, causing either oxidative stress in the case of increased reactive oxygen species (ROS) production or nitrosative stress when reactive nitrogen species (RNS) are produced excessively.^20^ This imbalance in the redox state is responsible for damaging various cellular macromolecules, including various carbohydrates, protein, DNA, and lipids, as summarized by Juan et al.,^21^ with implications for the development of different cancers.^22^ For instance, while HA is primarily retained within the tissue matrix due to its binding to the cell surface receptors, its cleavage by various free radicals can release lower-molecular weight (MW) chains, which can diffuse into the lymph and be uptaken by various cells types.^3^ The oxidative reactions of HA reported to date relied on their interactions with the hydroxyl radical (^•^OH),^23^ hypochlorous acid,^24^ and peroxynitrite (ONOO^•–^).^25,26^ Moreover, the catalytic effects of cupric and ferrous ions on H_2_O_2_-induced HA degradation were evaluated before.^27^

Nitric oxide (^•^NO) is one of the important RNS, which orchestrate various signaling pathways involved in controlling the proliferation and metastasis of tumors and angiogenesis, depending on its production level and duration of exposure.^28,29^ Moreover, it enhances various nitrosation and nitration reactions, particularly after interactions with molecular oxygen or oxygen radicals.^30^ However, unlike O_2_, ^•^NO functions as a chain-terminating agent owing to its quick interactions with various radicals.^31^ The interactions of ^•^NO with HA chains have been explored indirectly, focusing only on the reaction of ONOO^•–^ and HA. ONOO^•–^ is one of the main congeners of ^•^NO resulting from its reaction with the superoxide radical (O_2_^•–^). It was reported to ultimately degrade HA via the cleavage of the O-glycosidic linkages between its monomeric units.^25,26^ Moreover, while the reaction of ^•^NO with O_2_ generates HNO_2_, it was found that this reactive species is not active against HA due to the acetylation of its amine groups, but HNO_2_ can attack the free or N-sulfated amino groups such as those in heparin, initiating its oxidation and depolymerization.^32,33^

In the present work, the reactivity of ^•^NO against HA in the presence of molecular oxygen was studied. The ^•^NO scavenging by HA was evaluated first, followed by the investigation of the accompanying changes in its chemical structure, viscosity, and MW. These variations in properties were compared with those elicited following treatment of HA with ONOO^•–^, H_2_O_2_, and hyaluronidase. Next, the effects of HA and modified HA products on the adhesion, migration, and viability of the triple-negative breast cancer cells, MDA-MB-231 and HCC1806, in the presence of ^•^NO alongside the associated intracellular ^•^NO levels were examined. Finally, this study aims at getting an overview of the mechanism of interaction between HA and ^•^NO, which helps understand the physiological effects of ^•^NO on HA in cancer and paves the way for better optimization of new HA-based formulations proposed as treatments for breast cancer.

## 2 Materials and Methods

### 2.1 Materials

MDA-MB-231 cells (HTB-26) were from the American Type Culture Collection. iNOS-transfected HCC1806 were provided by Dr Faizan Khan, Lambe Institute for Translational Medicine. The molecular weights 16, 500, 700, 1000, and 1500 kDa were obtained from Lifecore Biomedical, Chaska, US. Hemin, Na_2_HPO_4_·2H_2_O, NaH_2_PO_4_·2H_2_O, phosphate-buffered saline (PBS), sodium hydroxide (NaOH), sodium azide (NaN_3_), deuterium oxide (D_2_O), anhydrous dimethyl sulfoxide (DMSO), ethylenediaminetetraacetic acid (EDTA), bromophenol blue, glycerol, H_2_O_2_, bovine serum albumin (BSA), potassium nitrite, ascorbic acid, 2,2-diphenyl-1-picrylhydrazyl (DPPH), hyaluronidase from bovine testes (400–1000 units/mg solid), tris(hydroxymethyl)aminomethane (TRIS), glycerol, agarose, ethanol, Stains-All stain, 2-(*N*-morpholino)ethanesulfonic acid (MES), 4-(4,6-dimethoxy-1,3,5-triazin-2-yl)-4-methylmorpholinium chloride (DMTMM), paraformaldehyde (PFA), RPMI-1640 medium, penicillin/streptomycin, l-glutamine, fetal bovine serum (FBS), and Hanks’ balanced salt solution without Ca^2+^ and Mg^2+^ (HBSS) were all purchased from Sigma-Aldrich. Water, CHROMASOLV for HPLC, acetic acid, luminol, recombinant human epidermal growth factor (EGF), and a Griess assay kit (G7921) were obtained from Fisher Scientific. 4-Amino-5-ethylamino-2′,7′-difluorofluorescein diacetate (DAF-FM DA) and ethidium bromide were provided by Invitrogen. The following ^•^NO donors were used during the study: sodium nitroprusside (SNP, Merck), (*Z*)-1-[*N*-(2-aminoethyl)-*N*-(2-ammonioethyl)amino]diazen-1-ium-1,2-diolate (DETA-NO), and *S*-nitroso-*N*-acetyl-d,l-penicillamine (SNAP) (Cayman Chemicals). 3-Morpholinylsydnonimine chloride (SIN-1) was supplied from Biotium.

**Scheme 1:**
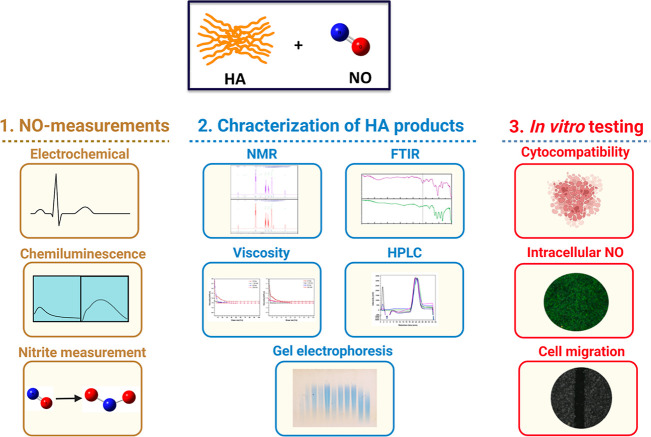
General Methods and Characterization Techniques Employed for (1) Measurement of the ^•^NO Scavenging by 1000 kDa HA, (2) Characterization of HA Following Modification by ^•^NO, and (3) In Vitro Testing of the Effects of the Different Products on Breast Cancer Cells. Schematic Created with BioRender.com.

The general methods and characterization techniques involved in the study are summarized in Scheme 1.

### 2.2 ^•^NO Measurements

SNAP served as the ^•^NO donor for building the standard curves in RPMI culture medium and 1× PBS. It was dissolved first in DMSO. The working solutions were freshly prepared in the tested solutions before each procedure for standard curve generation. DETA-NO solution was prepared by dissolving in 0.01 M NaOH, making aliquots, and finally diluted directly in the tested solution just before the main experiment. The stock solutions were consumed within 3 weeks of preparation. SNP and SIN-1 were dissolved directly in the testing solution for preparing the finally tested concentration.

#### 2.2.1 Electrochemical Detection

An ISO-NOP007 micro-sensor and a TBR 1025 Free Radical Analyzer from World Precision Instruments (WPI) Ltd., USA, evaluated the solution’s binding affinity between ^•^NO and 1000 kDa HA. The PowerLab hardware device (model: ML870) was the data acquisition system utilizing the LabChart 7 software (ADInstruments, Colorado Springs, USA) to analyze the generated voltage data. The sensor calibration in each solution was performed once a week following the injection of SNAP and 0.1 M CuSO_4_ with side monitoring of the temperature changes. To reduce the background current, before each test, the sensor was polarized overnight in the testing solution under a constant stirring speed, and the probe was set to ^•^NO with a current range of 100 nA and the sampling at 10 Hz. This was followed by purging the solution for 30 min with nitrogen gas before the injection of the SNAP calibrator or the tested concentration of the other ^•^NO donors and recording the voltage response over 3 h with intervals of 5 s. To test the effects of HA on the ^•^NO levels in the solution, DETA-NO was injected into the PBS solution to reach its final tested concentration. After 20 min, the HA solution was injected with monitoring of the accompanying change in voltage for 3 h. A HA solution was prepared overnight in PBS under continuous stirring at 4 °C, followed by direct dilution in the testing solution containing DETA-NO. For validation of the signal generated and its source, HA was injected into the solution without the presence of any ^•^NO donor, and the voltage readings were monitored. Moreover, to ensure that the ^•^NO levels were the only inducers of the signal, the solution was purged with nitrogen gas after injection of the NO donor. The drop in the signal relates to that exclusively. For comparison, the release of ^•^NO from 300 μM DETA-NO injected into a polarized PBS solution containing either 1000 μg/mL HA only or a mixture of HA and DMTMM was evaluated. DMTMM was used to activate the −COOH groups; HA and DMTMM were mixed in PBS solution for the final concentrations of 1 mg/mL and 2.78 mM, respectively. Following the incubation for 6 h at 37 °C, the electrode was polarized overnight in this mixture, followed by DETA-NO injection and voltage recording.

#### 2.2.2 CL Detection

Different concentrations of 1000 kDa HA were mixed with 1 mM SNP in phosphate buffer (50 mM, pH 7.4), followed by a separate injection of H_2_O_2_ and luminol through the stopped-flow system in the microplate reader (Varioskan Flash, Thermo Scientific). Next, the luminescence intensity was measured every 5 min over 2 h at 37 °C using the luminescence option. The HA concentrations tested were 100, 200, 500, and 1000 μg/mL against 1 mM SNP. The luminescence reaction was optimized, and the used concentrations of H_2_O_2_ and luminol were 50 and 1 mM, respectively. Moreover, the luminescence signal was monitored in the absence of SNP, where only different concentrations of HA were mixed with the chemiluminescence (CL) reagents.

#### 2.2.3 Nitrite Measurements

According to the manufacturer’s protocol, the generation of nitrite following the incubation of 1000 kDa HA with DETA-NO in PBS was detected using the Griess assay. First, the nitrite concentration resulting from the degradation of different concentrations of DETA-NO was measured following the dilution of the DETA-NO stock in PBS and incubation at 37 °C for 2 h. Next, DETA-NO was dissolved in PBS for a final concentration of 300 μM, mixed with 50, 100, 250, 500, 750, 1000, or 2000 μg/mL HA, and incubated for 2 h by the measurement of the nitrite concentrations. Finally, to monitor the kinetics of nitrite generation, DETA-NO was mixed with either 500 or 1000 μg/mL HA, and the nitrite concentration was measured after 1, 2, 4, 6, and 24 h of incubation at 37 °C. For nitrite detection, the working reagent was freshly prepared by mixing equal volumes of solutions A and B and adding 20 μL directly to premixed 150 μL of each tested sample and 130 μL of PBS, incubation at room temperature for 30 min, and measuring the absorbance at 548 nm using a plate reader. The blanks in the HA-containing systems were the corresponding ^•^NO-free HA-containing solutions.

### 2.3 Radical Scavenging Activity of HA Solutions

First, a standard curve of DPPH was generated employing the concentrations of 0, 5, 10, 15, 20, and 25 μM. Second, 1000 kDa HA was mixed with 25 μM DPPH for final concentrations of 100, 250, 500, 750, and 1000 μg/mL and incubated in the dark with shaking at 200 rpm for 30 min, followed by the measurement of the absorbance at 517 nm using a plate reader. The blanks were HA with the same tested concentrations but without DPPH. The 25 μM DPPH without any additives served as the 100% radical control, while ascorbic acid, with 5 and 10 mM final concentrations, was a positive control. The DPPH radical scavenging efficiency was calculated from the equation
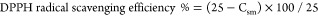
where 25 is the final concentration of HA-free DPPH solution in μM and *C*_sm_ is the concentration of remaining DPPH calculated from the standard curve.

### 2.4 HA Interactions with ROS, RNS, and Hyaluronidase

1000 kDa HA was initially dissolved in MES buffer (100 M, pH 6), followed by dilution in PBS and mixing with different ROS and RNS donors or hyaluronidase, incubated at 37 °C for 24 h, and freeze-dried. The final tested HA concentration was 1000 μg/mL. The NO donors were 30, 150, 300, and 600 μM of DETA-NO, 300 μM SNP, and 300 μM SNAP. The HA products were HA/30-DETA-NO, HA/150-DETA-NO, HA/300-DETA-NO, HA/600-DETA-NO, HA/SNP, and HA/SNAP, respectively. The NO donor solutions were prepared as described in Section 2.2, and the addition of any reducing agent did not accompany the release of ^•^NO from SNP and SNAP. For deactivation of DETA-NO, the stock solution was incubated at 37 °C for 8 days, with daily verification of ^•^NO release kinetics, and then mixed with the HA solution. In addition, the effects of hemin as one of the reported molecules that can bind to ^•^NO were studied; hemin, dissolved in DMSO, was diluted in PBS solution containing HA and 300 μM DETA-NO for the final concentrations of 4 and 8 μM and treated as described previously. These were denoted as HA/300-DETA-NO/4-Hemin and HA/300-DETA-NO/8-Hemin, respectively. H_2_O_2_ served as a donor of ROS and was tested at the final concentrations of 500 and 1000 μM, denoting the final products as HA/500-H_2_O_2_ and HA/1000-H_2_O_2_, respectively. SIN-1 was employed as a donor of both superoxide and ^•^NO, considering it as a donor of ONOO^•–^. It was dissolved in DMSO and diluted in PBS solution containing HA for a final concentration of 300 μM and denoted as HA/SIN-1. For comparison, HA degradation was initiated via its mixing with hyaluronidase to a final concentration of 100 U/mL, and the final product was denoted as HA/HAase. Moreover, the unmodified lyophilized HA was denoted as lHA, while the unmodified non-lyophilized HA (native HA) was denoted as nHA. The final products were characterized as follows.

#### 2.4.1 NMR

Each lyophilized HA product was dissolved in D_2_O for an approximate final concentration of 10 mg HA/mL in NMR glass tubes. The ^1^H NMR spectra were obtained at 400 MHz using a JEOL 400 MHz NMR ECX-400 Spectrometer between 12 and −1 ppm using a relaxation delay time of 3 s at room temperature.

#### 2.4.2 FTIR

The resulting powder was analyzed using an IRSpirit FTIR spectrophotometer, and the data acquisition was performed using Lab solutions IR software (version 2.25) (all from Shimadzu, Kyoto, Japan). The resulting curves were compared to the FTIR spectrum of lyophilized PBS, obtained without adding any of the reactants.

#### 2.4.3 Viscosity

The change in viscosity of 1000 kDa HA under the different treatments was monitored over time using an MCR-102, Anthon Paar rheometer (Graz, Austria) equipped with a Ø 50 mm stainless steel parallel plate geometry and an electrically heated plate. A 20 mL sample was tested, where the viscosity of 1000 μg/mL HA solution in PBS was monitored first at a constant temperature of 25 ± 0.04 °C with an increased shear rate from 0.001 to 100 1/s. This was followed by adding the different treatments and incubation at 37 °C to initiate the reactions, measuring the viscosity after 1.5, 6, and 24 h at 25 °C and testing three samples under each treatment group. Moreover, the viscosity of solutions containing 1000 μg/mL of HA with the average molecular weights of 16, 500, 700, 1000, and 1500 kDa was measured under previous conditions.

#### 2.4.4 High-Performance Liquid Chormatography

A high-performance liquid chromatography-refractive index detector (HPLC-RID) system was employed to monitor the changes in the MW of the different HA products and compare with that of the unmodified HA of known average MWs according to Čožíková et al.,^34^ with slight modifications. The separation was performed using an HPLC instrument (Shimadzu, prominence, LC-2030 plus), equipped with a high-sensitivity refractive index detector (RID-20A), and the data acquisition was performed using a Lab solutions Lite Main software (version: 5.93) (all from Shimadzu, Kyoto, Japan). Each HA product was dissolved in HPLC-grade water for a final concentration of 500 μg/mL. The injection volume was 100 μL, and the separation was carried out using an ultra-hydrogel column (cat. no. WAT011545) (7.8 × 300 mm; 10 μm) suitable for the MW range of 1–7000 kDa and thermostated at 40 °C. The mobile phase consisted of 100 mM NaH_2_PO_4_·2H_2_O with pH adjusted to 7.5 and 0.05% NaN_3_, with a 1 mL/min flow rate. Both the tested samples and mobile phase were filtered through Sarstedt Filtropur S 0.2 Syringe Filters (0.2 μm).

#### 2.4.5 Agarose Gel Electrophoresis

The change in the size of 1000 kDa HA following the different treatments was monitored by agarose gel electrophoresis. In brief, a 6 mm thick 1% agarose gel was prepared in 1× TAE buffer [40 mM TRIS, 20 mM acetic acid, 1 mM EDTA (pH 8)], and a 15-well comb (Fisher) was used to create the wells. After settling down for 20 min, the gel was transferred to the electrophoresis unit, filled with 1× TAE buffer, and pre-run for 6 h at 80 V. The HA samples were dissolved in PBS for a final concentration of 500 μg/mL; 6 μL of each one was mixed with 2 μL of the sample loading buffer (0.1% bromophenol blue, 40% glycerol), and the gel was run for 1 h at a constant voltage (100 V). The gel was then equilibrated in 30% ethanol for 1 h at RT, which was then exchanged with a fresh 30% ethanol solution containing 12.5 μg/mL Stains-All stain and left to rock for 24 h under exclusion of light. The stain was then decanted, and the gel was equilibrated in water for 1 h at RT away from light until it sank. Finally, the water was discarded, and the gel was exposed to normal light for around 10 min to remove the pink background, followed by its scanning on a standard color scanner (EPSON Perfection V850 pro).

### 2.5 In Vitro Study

#### 2.5.1 Cytocompatibility of nHA and Lyophilized HA Products

The cytocompatibility of different concentrations of 1000 kDa HA was evaluated first by culturing with MDA-MB-231 and iNOS-transfected HCC1806 cells. In brief, the cells were cultured overnight at a density of 25,000 cells/well in 48-multiwell plates in RPMI-1640 medium supplemented with 10% FBS, 100 IU/mL penicillin, 100 μg/mL streptomycin, and 2 mM l-glutamine at 37 °C in a humidified incubator with 5% CO_2_. In the case of HCC1806 cells, the medium was also supplemented with 2 μg/mL puromycin. Following the discarding of media, the cells were washed with PBS and cultured with HA. Here, a stock solution of HA was prepared in PBS, diluted to 4000 μg/mL in FBS-free RPMI, filtered, and diluted to different working concentrations, which were added to the cells for a further 48 h of culture, with the measurement of the metabolic activity after 24 and 48 h of culture. The tested concentrations of nHA were 25, 50, 100, 250, 500, 750, 1000, and 2000 μg/mL, while the lyophilized HA products were tested at the 500 μg/mL concentration only. When measuring the metabolic activity of cells, the culture media were discarded, PBS washed each well, and 200 μL of fresh 10% Cell-Quant AlamarBlue Cell Viability Reagent (Thermo Fisher) in PBS was added to each one. The cells were incubated for 3 h at 37 °C in 5% CO_2_, and the fluorescence was measured at an excitation of 550 nm and emission of 590 nm using the microplate reader. The percentage of metabolic activity was calculated by normalizing the fluorescence at each tested concentration to that of untreated cells. Three samples were tested for each concentration at each time point.

#### 2.5.2 Adhesion Assay

This assay was performed in 96-multiwell plates pre-coated with 1000 kDa HA to evaluate its effects on cell adhesion in the presence of DETA-NO. In brief, a stock solution of nHA in PBS was prepared and diluted to 200, 500, and 1000 μg/mL, and 50 μL of each one was added to a separate well. Similarly, a working concentration of 500 μg/mL HA/300-DETA-NO, produced following the incubation of HA with 300 μM DETA-NO for 2, 6, and 24 h and lyophilization, was used to coat the wells. The plates were then stored at 4 °C for 48 h, followed by washing with PBS and seeding of MDA-MB-231 cells at a density of 10,000 cells/well in the FBS-containing medium in the presence or absence of 300 μM DETA-NO. The number of adherent cells following their culturing for 2, 6, and 24 h was then determined by DAPI staining. The cells were washed with HBSS, fixed in 4% PFA for 15 min, washed and stained with 1 μg/mL DAPI for 7 min, and finally imaged using an Olympus IX81 Inverted Fluorescence Phase Contrast microscope. The number of attached cells was counted using ImageJ software. The percent cell adhesion was calculated by normalizing the average cell count per group to the average count of cells cultured directly without any coating. The percent cell adhesion was compared to that induced by BSA-coated wells.

#### 2.5.3 Measurement of the Intracellular ^•^NO

The MDA-MB-231 or iNOS-transfected HCC1806 cells were seeded at a density of 25,000 cells/well in a 48-multiwell plate in phenol red-free FBS-containing RPMI and cultured for 24 h for attachment. The media were then discarded, and the cells were washed with PBS. A stock solution of DAF-FM-DA was prepared in DMSO and diluted in phenol red-free 10% FBS-containing RPMI with a final concentration of 10 μM, and 200 μL was added to each well. After 1 hour of incubation at 37 °C in 5% CO_2_, the probe solution was discarded, and each well was washed three times with PBS at 10 min intervals. Finally, all PBS residues were removed, and 400 μL of phenol red and FBS-free RPMI medium containing different concentrations of 1000 kDa HA in the presence or absence of 300 μM DETA-NO was added to each well. The same procedures were repeated by treating MDA-MB-231 cells with 500 μg/mL of each lyophilized HA product and 300 μM DETA-NO. The changes in the fluorescence corresponding to the intracellular ^•^NO were monitored using a IncuCyte S3 Automated Live-Cell Analysis System. Four positions were randomly chosen per well with testing of three wells per group. The images were acquired every 1 h directly after adding the tested materials for 24 h. The assessment of fluorescence corresponding to intracellular ^•^NO was performed automatically by IncuCyte ZOOM via measuring the green object count for all cells stained green with DAF-FM and their intensity with normalizing the results to the total object count per image.

#### 2.5.4 Transwell Assay

The cell migration assay through transwells was performed according to Pijuan et al.^35^ The initial cell confluency was 80%, and following trypsinization, a 400 μL suspension of cells was seeded at a density of 50,000 cells in FBS-free RPMI onto the upper chamber of each transwell insert (membrane 8.0 μm pores, Cruinn Diagnostics). Following incubation for 1 h for attachment, each insert was transferred to a well of 24-multiwell plates containing 600 μL of FBS-containing RPMI only or in addition to 300 μM DETA-NO in the presence or absence of different concentrations of 1000 kDa HA. The cells were incubated further at 37 °C and 5% CO_2_, and the percentage of migrated cells through the membranes was calculated after 12 and 24 h in the case of MDA-MB-231 cells and only after 24 h in the case of iNOS-transfected HCC1806 cells. The cells were fixed as previously described in Section 2.5.2 and washed, and the non-migrated cells on the upper surface of the transwell membranes were gently scraped with a wet cotton swab before staining cells with DAPI. The cells on the lower surface were then imaged using an Olympus IX81 Inverted Fluorescence Phase Contrast microscope and counted using ImageJ software.

#### 2.5.5 Wound Healing Assay

In vitro wound-healing assay was employed to assess the effects of ^•^NO and HA on the migration of MDA-MB-231 and iNOS-transfected HCC1806 cells. The cells were trypsinized and counted, and 70 μL of FBS-containing RPMI containing 50,000 cells was added to each well of a sterile 2-well silicone insert (Ibidi) placed in a 24-multiwell plate, followed by overnight incubation in 5% CO_2_ at 37 °C for cell attachment. The inserts were then removed, and the cells were washed with PBS before adding 1 mL of phenol red-containing FBS-free RPMI containing 300 μM DETA-NO with and without 1000 kDa HA to each well. This was followed by imaging the whole well using the IncuCyte S3 Automated Live-Cell Analysis System at regular intervals of 1 h for 24 h in 5% CO_2_ at 37 °C. The images acquired were analyzed further by taking two snapshots from each image at each time point and measuring the change in the area of the gap using ImageJ software. Three samples were tested per group, the experiment was performed two times, and the averages of all measurements were taken.

### 2.6 Statistical Analysis

The SPSS Computer program (Version: 26) was used in the statistical analysis of the results. All data were expressed as the means ± S.D and analyzed using (Student’s) *t*-test. The differences were considered statistically significant at *p* < 0.05.

## 3 Results and Discussion

### 3.1 Electrochemical Detection of ^•^NO

The efficiency of HA to bind ^•^NO was evaluated using an electrochemical method, and its influence on the ROS and RNS involved in the H_2_O_2_/luminol-based CL reaction was assessed. DETA-NO has been employed for the main screening of the binding affinity of HA with ^•^NO. Its reported half-life of 20 h at 37 °C,^36^ making it ideal for the slow release of ^•^NO over a prolonged period, is considered advantageous for its in vitro applications. SNAP was employed for the generation of standard curves used in the quantification of ^•^NO in PBS, considering a degradation rate of 60% for SNAP once diluted in the solution as recommended by WPI. Figure S1 illustrates the changes in voltage reading following the consecutive addition of different concentrations of SNAP in PBS (Figure S1A) and one of the generated standard curves (Figure S1B).

**Figure 1:**
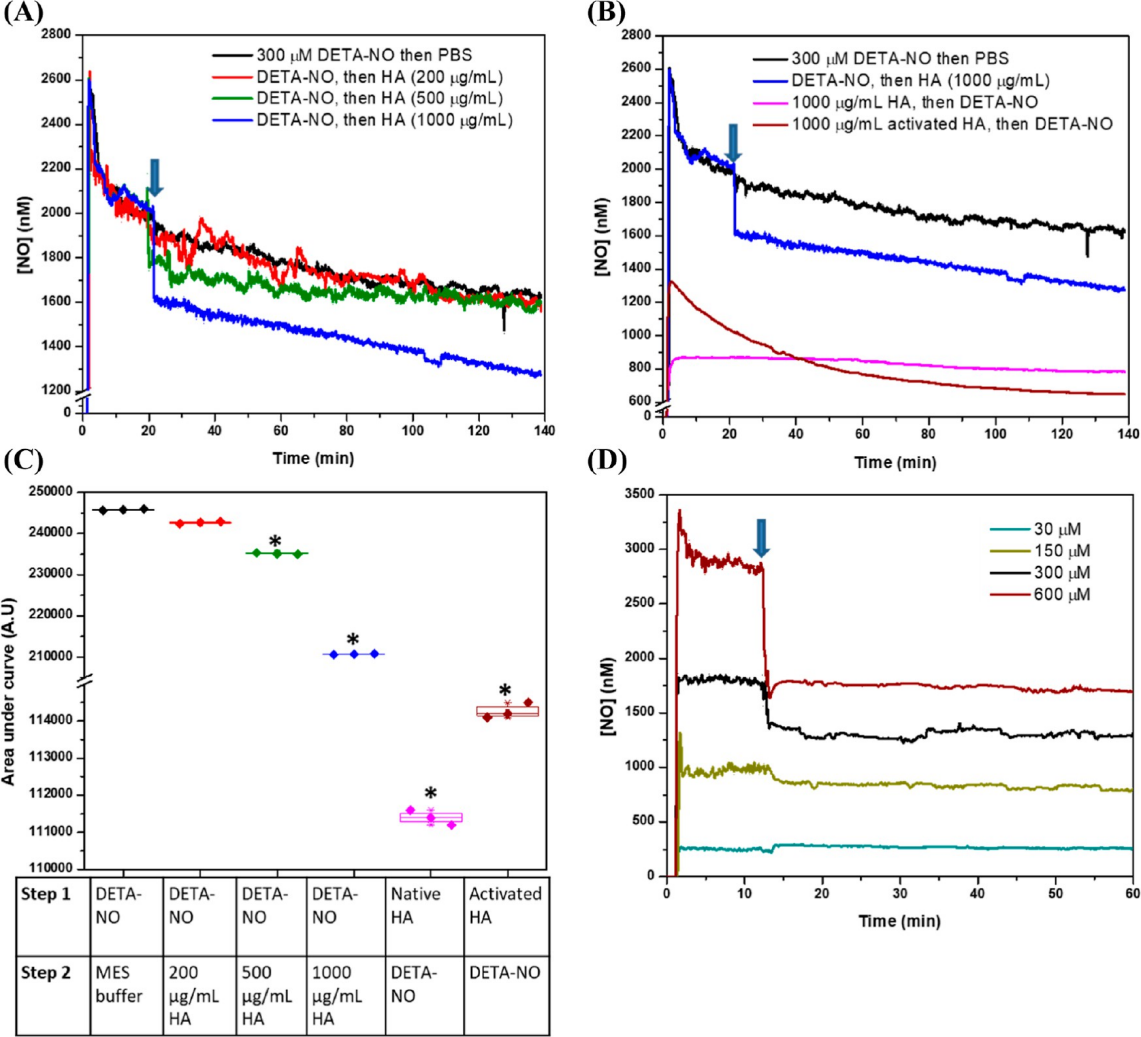
Temporal changes in the ^•^NO levels in PBS: (A) Average release profile of ^•^NO from 300 μM DETA-NO only (black color) and the changes in its concentrations after the further addition of 1000 kDa HA with the concentrations of 200 (red color), 500 (green color), and 1000 μg/mL (blue color). HA was added 18 min after DETA-NO injection, as indicated by an arrow. (B) Average release profile of ^•^NO from 300 μM DETA-NO, injected into an overnight-polarized PBS solution containing 1000 μg/mL HA (magenta color) or DMTMM-activated HA (brown color). (C) Box and whisker plots showing the mathematical area under the curves, and data represented are the means ± S.E.M. (D). ^•^NO levels released from 30, 150, 300, and 600 μM DETA-NO, followed by the injection of HA for a final concentration of 1000 μg/mL, as indicated by an arrow. The data are represented as the mean ^•^NO concentration of three measurements per group. *p < 0.05 compared to the control group (DETA-NO only group) using a two-tailed unpaired Student *t*-test.

In both tested solutions, FBS-free RPMI and PBS, the injection of DETA-NO caused an immediate increase in the voltage signal owing to the released ^•^NO, followed by a slow decay over time. However, the voltage and the accompanying generated ^•^NO levels decreased once HA was injected further into the PBS solution. This decrease in the ^•^NO concentration was proportional to the HA concentration, with 1000 μg/mL HA showing the highest drop-in ^•^NO concentration level (Figure 1A). Moreover, when the HA solution in PBS was polarized overnight, followed by the injection of DETA-NO, the maximum rate of ^•^NO release decreased by around 66.62 ± 1.2% compared to the HA-free system (Figure 1B). The calculated area under each curve is shown in Figure 1C, and the corresponding changes in the voltage readings are shown in Figures S2A and 1B. Similar inhibitory effects for ^•^NO release from DETA-NO were reported when the polarization solution contained the BSA or FBS-containing culture medium.^37^ However, it is still unclear how the components of the testing solution affect the degradation of the NO-releasing NONOate compounds. Interestingly, the effects of DMTMM-activated HA on the release of ^•^NO from DETA-NO were less than those of the native HA. Considering the possible covalent interactions between the activated carboxylic functional groups HA and DETA-NO, it can be concluded that this conjugation enhanced the degradation of DETA-NO and overcame the effects of the polymer itself on slowing NO release. However, further analysis of these observations is ongoing.

To check whether the decrease in voltage signal following HA injection relates to its possible interference with the electrode measurement, the signal was measured following the injection of HA for a final concentration of 1000 μg/mL without adding any ^•^NO-donor (Figure S2C). Although slight changes in the signal were observed in the case of HA alone, these were negligible compared to the drop in voltage following the addition of HA after DETA-NO.

Next, the interactions between ^•^NO, released from different concentrations of DETA-NO of the same batch, and HA in PBS are evaluated in Figure 1D. The concentration of released ^•^NO was proportional to that of DETA-NO, with a maximum release of 250, 1010, 1800, and 3250 nM from 30, 150, 300, and 600 μM DETA-NO, respectively. It should be noted that the release profile of ^•^NO from 300 μM DETA-NO is different from the one shown in Figures 1A and S2 due to batch-to-batch variation; however, the effects of HA are still comparable. HA injection caused an expected drop, followed by a slight decrease in ^•^NO concentration; nevertheless, this drop in ^•^NO levels was proportional to the DETA-NO concentration (Figure 1D). For instance, the ^•^NO levels from 150, 300, and 600 μM DETA-NO dropped significantly from 1010 ± 8 nM to 954 ± 112 nM (% of drop = 5.94%), from 1804 ± 17 nM to 1448 ± 15 (% of drop = 19.44), and from 2899 ± 15 nM to 1774 ± 13 nM (% of drop = 37.93) (*P* < 0.05), respectively (Table 2). Moreover, no significant change in the ^•^NO levels from 30 μM DETA-NO was detected following HA injection, except for a slight increase in the voltage signal owing to the HA itself, as illustrated previously. It is important to note that the ^•^NO measurements were performed in PBS, so the influence of HA on ^•^NO levels can be investigated with minimal interference from the culture medium components on DETA-NO degradation. A comparison of ^•^NO release from 30 and 300 μM DETA-NO in phosphate buffer and the FBS-containing medium showed a degradation rate of the NO-donor in the former solution higher than that in the culture medium (Alsharabasy et al., 2022).^37^

**Table 1:** Description of the Reaction Components between 1000 μg/mL 1000 kDa HA and Different NO Donors, H_2_O_2_, and Hyaluronidase

final product	reactants
HA/deactivated-300-DETA-NO	HA + 8 day-deactivated 300 μM DETA-NO
HA/30-DETA-NO	HA + 30 μM DETA-NO
HA/150-DETA-NO	HA + 150 μM DETA-NO
HA/300-DETA-NO	HA + 300 μM DETA-NO
HA/600-DETA-NO	HA + 600 μM DETA-NO
HA/300-DETA-NO/4-Hemin	HA + 300 μM DETA-NO + 4 μM hemin
HA/300-DETA-NO/8-Hemin	HA + 300 μM DETA-NO + 8 μM hemin
HA/500-H_2_O_2_	HA + 500 μM H_2_O_2_
HA/1000-H_2_O_2_	HA + 1000 μM H_2_O_2_
HA/HAase	HA + 100 U/mL hyaluronidase
HA/SIN-1	HA + 300 μM SIN-1
HA/SNP	HA + 300 μM SNP
HA/SNAP	HA + 300 μM SNAP

**Table 2:** Change in Voltage Readings in PBS Solution Containing Different Concentrations of DETA-NO before and after the Injection of 1000 kDa HA to a Final Concentration of 1000 μg/mL and the Accompanying ^•^NO Concentration (nM)^a^

	voltage (V)		NO concentration (nM)	
DETA-NO concentration (μM)	before	after	before	after
30	1.032	1.022	291 ± 4.2	286 ± 8
150	2.52	2.41	1010 ± 8	954 ± 12*
300	4.165	3.427	1804 ± 17	1448 ± 15*
600	6.43	4.1	2899 ± 15	1774 ± 13*

a Data are represented as mean ± SD, *n* = 3. *, *P* < 0.05 versus the ^•^NO concentration before HA injection.

The accompanying change in the voltage readings is shown in Figure S2D. Similar results were observed following the addition of HA to the PBS solution pre-injected with deactivated DETA-NO. These results suggest that the NO scavenging by HA is limited by the concentration of ^•^NO and the number of its molecules surrounding the HA chains.

The detection of nitrite, as the stable end product of ^•^NO following its reaction with atmospheric oxygen, via Griess assay relies on its interactions with sulfanilamide and *N*-(1-naphthyl)ethylenediamine dihydrochloride, producing an azo compound with a reddish-pink color. However, owing to the possible interactions of HA with these reagents, the blanks included HA solutions with the same tested concentrations against DETA-NO. Reaction 1 shows how nitrite is formed via a third-order kinetics (4 kequiv = 8 × 106 M^–2^·s^–1^ at 25 °C)^38^
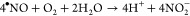
1

**Figure 2:**
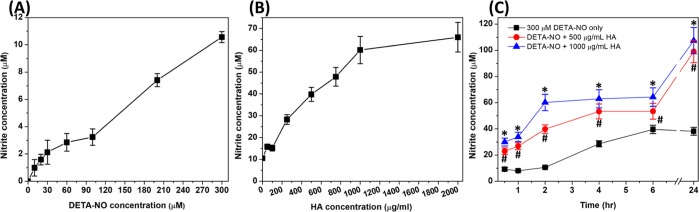
Detection of nitrite generated from (A) different concentrations of DETA-NO, incubated for 2 h in phosphate buffer (50 mM, pH 7.4) at 37 °C, (B) mixing of different concentrations of 1000 kDa HA with 300 μM DETA-NO and incubation for 2 h at 37 °C, and (C) incubation of a mixture of 500 or 1000 μg of HA with 300 μM DETA-NO for 24 h, with the measurement of the nitrite concentration at certain intervals using Griess assay. After the incubation period, 150 μL of each sample was mixed with 130 μL of phosphate buffer and 20 μL of the working reagent, incubated at RT for 30 min, and the absorbance was then measured at 548 nm. Data are represented as mean ± SD, *n* = 3. *^,#^, *P* < 0.05 in the case of 500 and 1000 μg/mL HA, respectively, versus the HA-free group using a two-tailed unpaired Student *t*-test.

Figure 2A shows the changes in nitrite levels corresponding to different concentrations of DETA-NO in PBS after incubation at 37 °C for 2 h. The nitrite concentration was proportional to the DETA-NO concentration owing to the different levels of released ^•^NO (Figure 1D). To compare the possible downstream effects of NO scavenging, the nitrite levels were measured after the incubation of different concentrations of HA, denoted as HA concentrations with 300 μM DETA-NO. Here, although the nitrite concentration in the case of DETA-NO only was 10.57 ± 0.41 μM, mixing with HA caused it to increase significantly proportional to HA concentration, despite the proven ability of HA to decrease the ^•^NO levels as measured electrochemically (Figure 2B). For instance, the concentrations of 1000 and 2000 μg/mL of HA showed the highest levels of nitrite, 60.12 ± 6.22 and 65.98 ± 6.79 μM, respectively, but with no significant differences between each other. To evaluate the kinetics of nitrite generation, Figure 2C shows the changes in nitrite concentration in the case of DETA-NO mixed with 500 and 1000 μg/mL of HA, where both concentrations increased the nitrite levels significantly over time compared to the HA-free systems, and there were no significant differences between them after 24 h. These results suggest a specific mechanism for ^•^NO oxidation, with possible modification of HA after binding to ^•^NO, as explained in the following sections.

### 3.2 CL Detection of ^•^NO

The first procedure for the CL detection of ^•^NO in the presence of HA was to evaluate the possible interactions between the CL reaction components and HA and their effects on the ^•^NO signal. 1000 kDa HA at different concentrations caused a drop in the luminescence signal generated via the H_2_O_2_/luminol reaction (Figure S3A). This can relate to the interactions of HA with various ROS.^23,27^ These radicals are the main reactants responsible for generating the H_2_O_2_/luminol-based luminescence signal,^39^ causing its quenching. The DPPH assay was employed to compare the efficiency of different HA concentrations to scavenge DPPH, as reported previously.^40^ This effect was concentration-dependent with insignificant differences between HA at 500, 750, and 1000 μg/mL showing around 75% of DPPH radical scavenging efficiency (Figure S3B), confirming the CL results.

**Figure 3:**
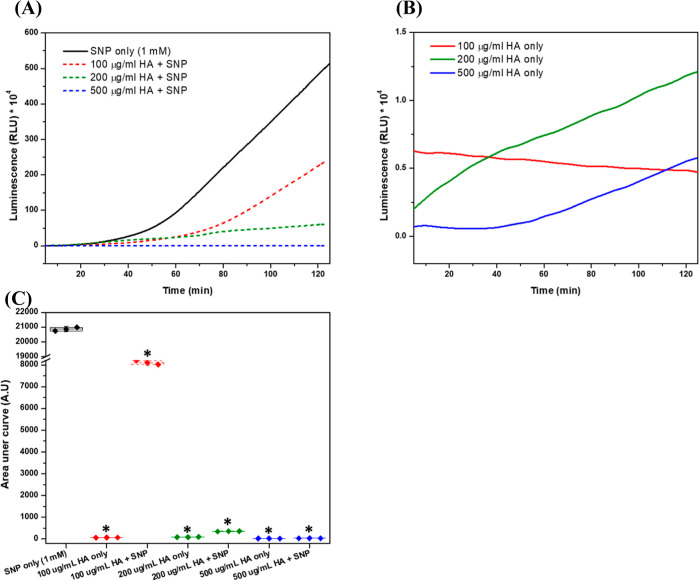
Effects of 1000 kDa HA on the ^•^NO-enhanced luminescence signal. (A) Luminescence intensity was measured over 125 min following the incubation of a mixture of 1 mM SNP with 100, 200, and 500 μg/mL HA in phosphate buffer (50 mM, pH 7.4). (B) Corresponding luminescence intensity in the absence of ^•^NO. (C) Box and whisker plots showing the mathematical area under the curves, and data represented are the means ± SD, *n* = 3. *, *P* < 0.05 compared to the control group (DETA-NO only group) using a two-tailed unpaired Student *t*-test.

Next, SNP was employed in the CL assay due to the burst release of ^•^NO once it is dissolved in the phosphate buffer, leading to a gradual enhancement in the luminescence signal once it reacts with luminol and the ROS involved in the reaction. The reactions involved have been widely investigated and employed in measuring ^•^NO in different biological systems.^41,42^ Moreover, the luminescence intensity increases over time, giving this reaction an advantage when studying the effects of different concentrations of HA and overcoming the problem of ROS scavenging by the HA chains themselves, having the advantage of being able to test many samples at the same time. However, it should be emphasized here that the results of this assay will only be valid in the case of relatively low concentrations of HA, characterized by the lowest efficiencies to inhibit the initial luminescence signal generation, compared to the higher ones which abolished the signal completely. Accordingly, the results corresponding to the effects of 100, 200, and 500 μg/mL HA against ^•^NO generated from SNP are shown in Figure 3A,B and the corresponding area under each curve is in Figure 3C. While the effects of all these concentrations on the ^•^NO-free signals were similar, differences were observed in the presence of SNP. Here, HA at 500 μg/mL caused complete inhibition of the luminescence signal, followed by HA at 200 μg/mL, and finally, HA 100 μg/mL showed the lowest effects. However, these changes in luminescence were all significantly different from the SNP-only signal. Similar results were reported by Saturnino et al., where the non-scavenged ^•^NO by HA was measured in the form of nitrite.^43^ Furthermore, a possibility still exists for the reaction of HA chains with peroxynitrite (ONOO^•–^), which is generated as a downstream RNS, produced in the buffer as in reaction 2, and induces luminol oxidation (reaction 3).^44^ Moreover, ONOO^•–^ has been reported to interact with HA causing partial fragmentation of its chain;^26,45^ however, the probability of these reactions is low due to the short period of the assay
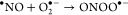
2
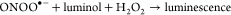
3

### 3.3 HA Interactions with ROS, RNS, and Hyaluronidase

#### 3.3.1 Chemical Modification of HA

Generally, ^1^H-NMR spectroscopy is not considered an ideal technique for characterizing the high-MW hyaluronate owing to the fast relaxation of these macromolecules and their restricted motions giving very weak signals.^46^

**Figure 4:**
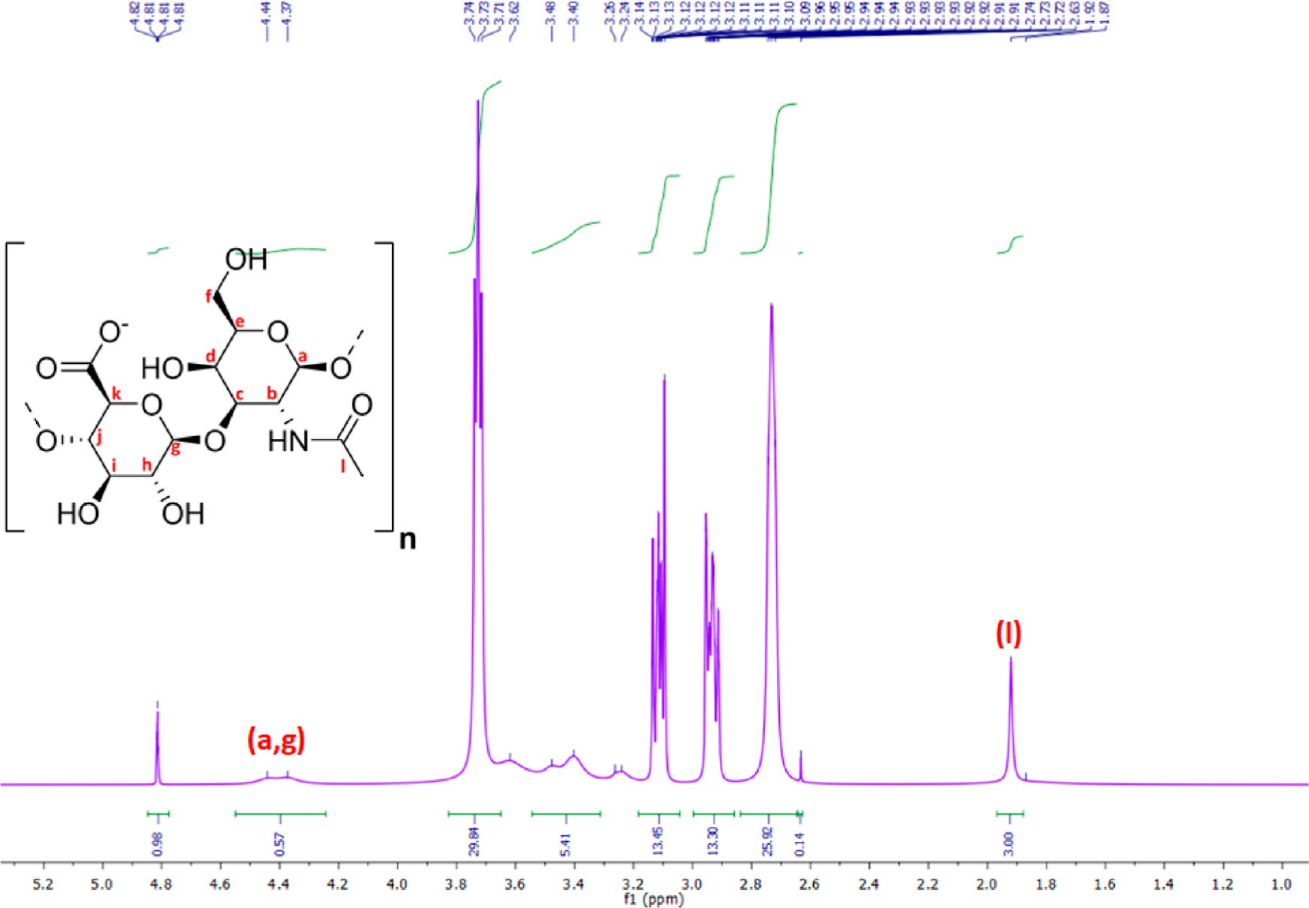
^1^H NMR spectrum of untreated and lyophilized 1000 kDa HA at 400 MHz and a temperature of 25 °C. The sample was dissolved in D_2_O.

Generally, significant differences in the NMR spectra of 1000 kDa HA were observed comparing the directly dissolved polymer in D_2_O, nHA, and HA dissolved in MES buffer, diluted in PBS, and freeze-dried before dissolving in D_2_O, referred to as lHA. While the peaks in the former case were superimposed, making it difficult to interpret them, the spectrum of lHA showed additional peaks corresponding to the precipitated MES with HA following freeze-drying (Figure S4). The chemical shift values are reported in parts per million (ppm). The new MES peaks were within the range of 2.6–3.9 ppm, specifically at 2.78, 2.9, 3.1, and 3.75 ppm, and were observed in all HA products following freeze-drying. Moreover, these peaks overlapped with the original low-intensity broad HA peaks, corresponding to the glycosidic hydrogens. However, the detailed 1H-NMR spectrum of lHA shows a separate peak for the methyl protons of the group (−NHCOCH_3_) at 1.92 ppm (l, singlet) (Figure 4). Furthermore, the anomeric 2H (a, g) was identified as a doublet peak at 4.37 and 4.44 ppm (non-reducing ends). In comparison, the anomeric H at the reducing end was detected at 4.81 ppm (quartet).^47^ No peaks corresponding to the exchangeable protons from −NH and −OH groups were detected.

**Figure 5:**
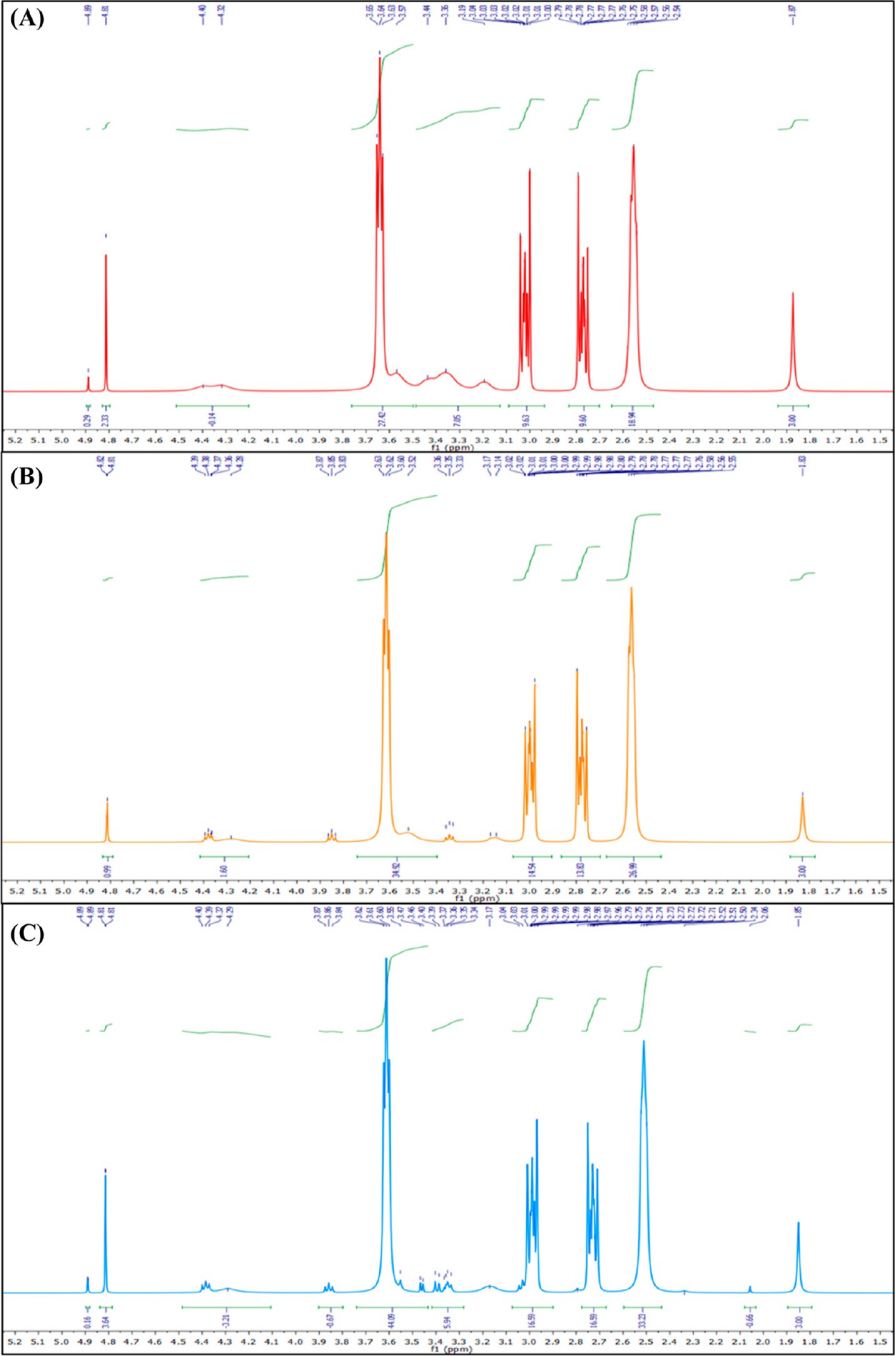
Comparison of the ^1^H NMR spectra of (A) HA/30-DETA-NO, (B) HA/300-DETA-NO (6 h), and (C) HA/300-DETA-NO (24 h). Following the lyophilization of the different HA products, they were dissolved in D_2_O, and the NMR spectra were recorded at 400 MHz. A description of each HA product is given in Table 1.

Upon the treatment of HA with 30 and 300 μM DETA-NO and lyophilization, shifting for the peaks corresponding to the glycosidic hydrogens was observed without any changes in the anomeric peak and the peak for the group (−NHCOCH_3_) (Figure 5A–C). Moreover, this shifting was observed after the treatment of HA with 300 μM DETA-NO for 6 h and remained constant for 24 h (Figure 5B,C). In the case of HA/30-DETA-NO, this shifting was not accompanied by significant differences in the peak numbers and multiplicities compared to HA itself, except for the resolution of the anomeric H peak into two singlet peaks at 4.89 and 4.81 ppm.

**Figure 6:**
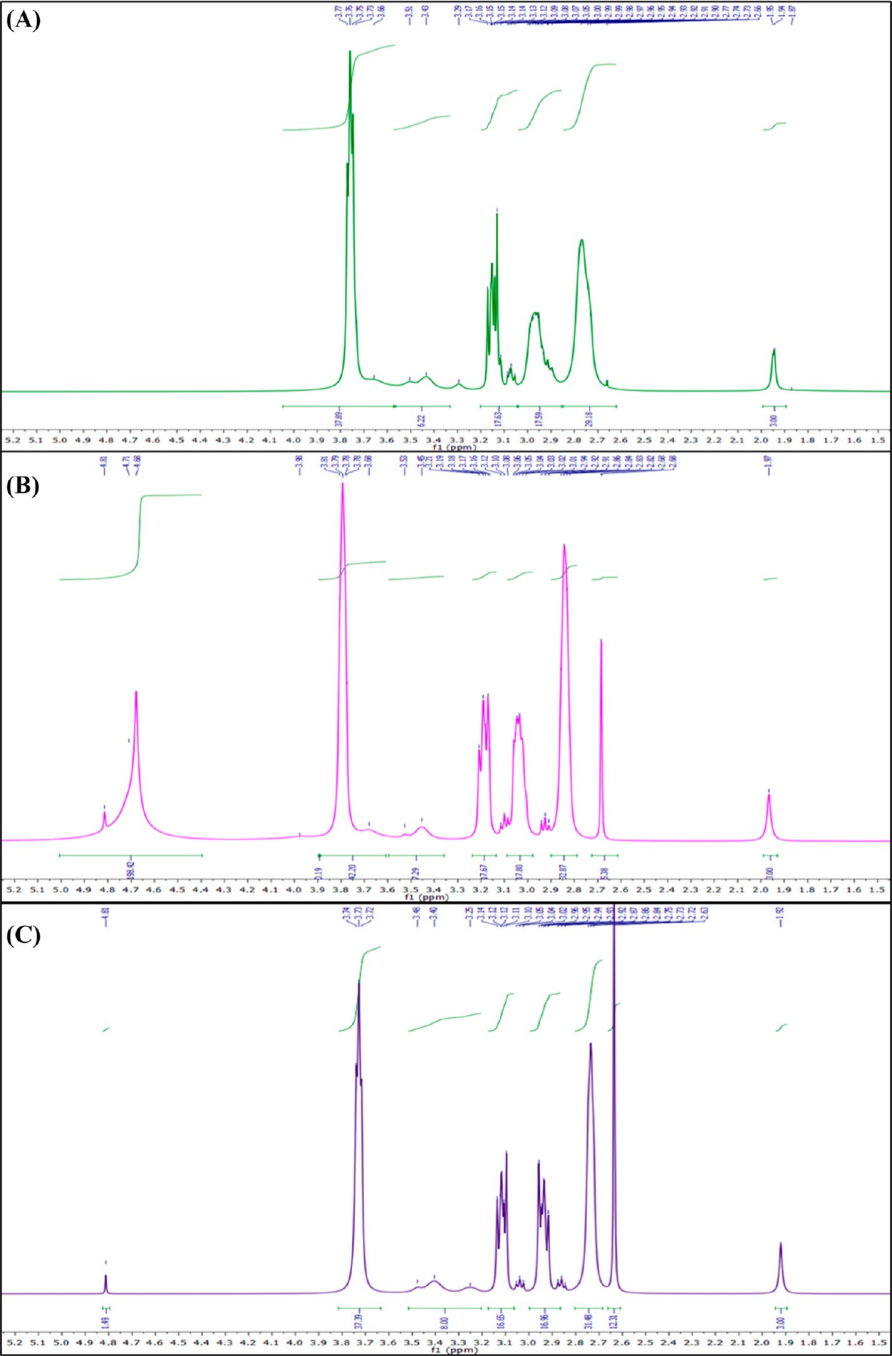
Comparison of the ^1^H NMR spectra of (A) HA/600-DETA-NO, (B) HA/300-DETA-NO/4-Hemin, and (C) HA/300-DETA-NO/8-Hemin. Following the lyophilization of the different HA products, they were dissolved in D_2_O, and the NMR spectra were recorded at 400 MHz. A description of each HA product is given in Table 1.

However, in HA/300-DETA-NO, a new peak was detected at 2.06 ppm (singlet), which corresponds to the group (−NHCOCH_3_) located in a terminal location and the central singlet peak at 1.85 ppm. This suggests a possible cleavage of the O-glycosidic bond, similar to the results observed by Sliadovskii et al.^48^ Moreover, similar peaks were observed within the range of 1.92–2.05 ppm in the case of HA/300-SNAP, without any differences in HA/300-SNP (Figure S5B,C). In addition, although a separate peak was not detected in the case of HA/600-DETA-NO, the proton of −NHCOCH_3_ was detected as a doublet centered at 1.95 ppm, supporting the findings of HA/300-DETA-NO (Figure 6A). Furthermore, the anomeric H peaks could not be detected in HA/600-DETA-NO, which indicates possible degradation of HA following the treatment.

The observed changes in the spectrum of HA/300-DETA-NO were absent when HA was treated with DETA-NO in combination with hemin, referring to possible interference of hemin with ^•^NO preventing its modifying effects for HA (Figure 6B,C), with final main peaks similar to those of the untreated HA. Moreover, a new singlet peak was observed at 2.68 and 2.63 ppm in the case of 4 and 8 μM hemin, respectively, with multiple peaks within the ranges of 2.9–2.96 and 3.07–3.14 ppm, corresponding to the hemin entrapped within the HA chains following lyophilization, which can be easily detected with NMR.

In the case of HA/300-DETA-NO/4-Hemin, the ratio of integration between the peak at 2.68 ppm and the peak of −NHCOCH_3_ was 1.66, while it reached 4.1 ppm at 8 μM hemin. However, owing to the low concentration of hemin itself and its dimerization in the aqueous solution, the assignment of its peaks is not possible here.

Peroxynitrite (ONOO^•–^) has been reported previously as one of the strong non-specific degradative agents of HA,^33,45^ with possible implications for some diseases. SIN-1 is reported as a donor of O_2_^•–^. and ^•^NO, which combine producing ONOO^•–^, considered a peroxynitrite donor.^49^ However, the NMR spectra of untreated HA and SIN-1-treated HA were nearly identical (Figure S6A), so the NMR technique was not sensitive enough to monitor any ONOO^•–^-mediated modification of HA, the results of which were similar to the results reported by Corsaro et al.^50^ However, the following sections will clarify these modifications.

In the case of HA treated with H_2_O_2_, there were no differences in the NMR spectra before and after the treatment (Figure S6B). The mechanism of free-radical-mediated degradation of HA has been described before, with metallic ions such as cupric and ferrous ions playing essential roles as catalysts for the degradation in the presence of H_2_O_2_.^23,27^ However, in the current study, the degradation of HA was performed in the presence of H_2_O_2_ only to compare its effects to those of ^•^NO and ONOO^•–^ donors.

The testicular HAase performs its functions by hydrolysis of HA at the β-1,4-glycosidic bond, producing tetrasaccharide units.^51^ By comparing the ^1^H-NMR of HA before and after hydrolysis using testicular HAase, a group of peaks can be observed corresponding to different types of anomeric protons, including a quartet at 4.45 ppm, a doublet at 4.81 ppm, and a new peak centred at 5.13 ppm (Figure S6C), attributed to the proton signal of the α-d-anomer of HA oligomers as explained by Vikha et al.^51^

**Figure 7:**
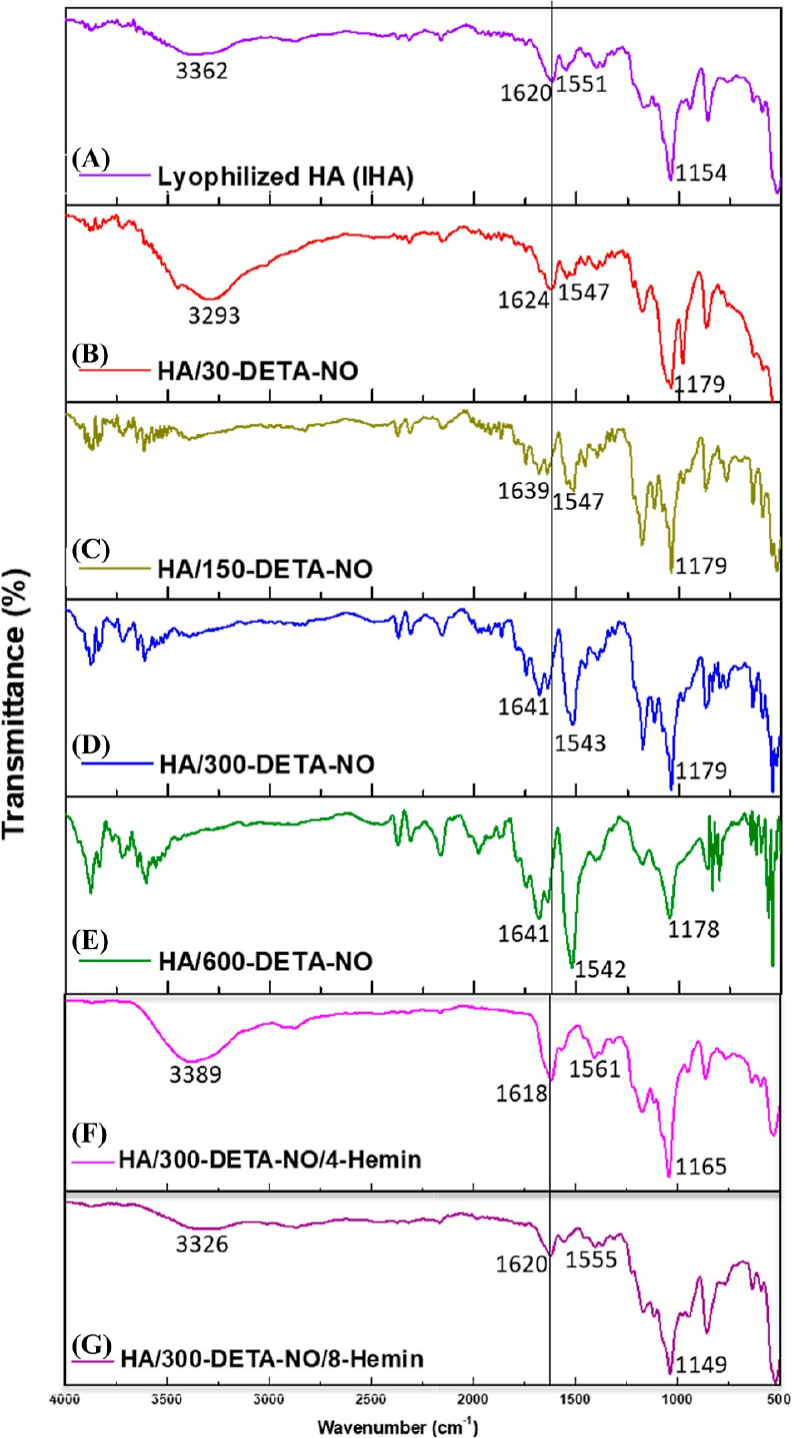
FTIR spectrum of (A) 1000 kDa lHA, (B) HA/30-DETA-NO, (C) HA/150-DETA-NO, (D) HA/300-DETA-NO, (E) HA/600-DETA-NO, (F) HA/300-DETA-NO/4-Hemin, and (G) HA/300-DETA-NO/8-Hemin. A description of each HA product is presented in Table 1.

FTIR has been employed since it is a relatively easy analytical method to obtain direct information on the chemical changes in specific functional groups following various chemical treatments of polymers. Figure 7 shows the FTIR spectra of 1000 kDa lHA and nHA, treated at a 1000 μg/mL concentration with 30, 150, 300, and 600 μM DETA-NO for 24 h, and the prominent detected peaks are summarized in Table S1. The assignment of bands for HA was based on the data reported previously by Gilli et al., Alkrad et al., Wu et al., and Chen et al.^27,52−54^ It should be borne in mind that some differences were observed before and after the lyophilization of HA. For instance, the peak at 3362 cm^––1^ corresponding to OH and NH stretching was broader following HA lyophilization than the nHA. Moreover, the band at 1401 cm^–1^, attributed to C–O stretching, did not change with lyophilization.

Furthermore, a new weak band was observed at 1745 cm^–1^ and can be assigned to asymmetric C=O stretching, similar to that observed by Gilli et al.^52^ referring to partial protonation of the −COOH group of HA following the dissolution in PBS and lyophilization, where the main band at 1620 cm^–1^ was also maintained. However, this extra band at 1745 cm^–1^ can also be attributed to the C=O stretching from the lyophilized remnants of PBS.^55^ These changes were also observed after the treatment of HA with DETA-NO (Figure 7B–E), but with a stronger band observed within the range of 1740–1750 cm^–1^, which, in these cases, confirm the attribution to amide I of a new C=O group generated following the treatment of HA.

In the case of DETA-NO, the detection of this band started mainly in HA/150-DETA-NO, and its intensity was proportional to the concentration of DETA-NO. Additionally, the broader bands within the range of 2900–3400 cm^–1^ under these treatments relate to a possible disturbance in the H-bonding following the oxidation of the monomer rings. However, in the presence of hemin, the peak for OH and NH stretching was observed with a stronger intensity due to its protective effects on the NO-induced changes in the hemin structure. Although the FTIR spectrum of hemin also shows a distinct peak within this range, this was weaker than that of HA. Moreover, as the concentration of the tested hemin is low compared to that of HA, its corresponding FTIR peaks will be negligible. By comparing the intensity of the peak at 1740 cm^–1^ to the main one at 1641 cm^–1^ following the addition of 300 μM DETA-NO to both 500 and 1000 μg/mL HA and incubation for different time points, it is evident that the generation of the C=O group is more significant at the lower HA concentrations (Figure S7A,B). Moreover, the intensity of the peak at 1740 cm^–1^ increased over time, relating to an enhancement of the oxidation reaction with a more extended incubation period. With the addition of hemin and 300 μM DETA-NO, these changes disappeared with insignificant differences from the untreated HA (Figure 7F,G, Table S2), parallel to the observed NMR results reported previously.

Similar results were observed in the case of HA/SNP and HA/SNAP (Figures S8B,C and Table S3). While both SNP and SNAP can spontaneously release 1 mol of ^•^NO per mole of the parent compound, this depends on the composition of the dissolution medium. For SNP, this requires it to be dissolved in a medium with a specific pH in the presence of a particular reducing agent such as Na_2_SO_4_ and ascorbic acid,^56^ which was not the case in the current study. This can account for the weaker intensity of the band at 1740 cm^–1^ in the case of HA/SNP than that of HA/SNAP. Furthermore, a new characteristic peak was observed at 1918 cm^–1^, attributed to the NO-stretching vibration in SNP itself,^57^ owing to its low degradation rate, so some SNP molecules maintained their composition and precipitated within the HA matrix during the lyophilization (Figure S8B). In the case of HA/SNAP, the half-life of SNAP is around 6 h in an aqueous solution with a pH within the range of 6–8 at 37 °C,^58^ while it is approximately 20 h in the case of DETA-NO.^36^ Accordingly, during the whole incubation period of 24 h, higher concentrations of ^•^NO would be expected to be released from SNAP than from DETA-NO, considering the differences in mole ratio between ^•^NO and its parent compound in each case. Therefore, the accompanying effects of ^•^NO on the HA chains would be faster in the case of SNAP, and this may explain the higher intensity of the C=O band at 1740 cm^–1^ in HA/SNAP (Figure S8C) than that of HA/150-DETA-NO. In contrast, the FTIR spectrum of HA/SIN-1 did not show any differences from HA itself (Figure S8D). SIN-1 is not only a ^•^NO donor but also releases superoxide species, which react directly with ^•^NO, generating ONOO^•–^ ions, which seem to have another mechanism of reaction with HA, despite its reported efficiency to degrade HA.^25,33,45^

For comparison, the effects of H_2_O_2_ on HA were also investigated, and many characteristic bands of HA could not be observed relating to a possible change in the chemical structure of HA following its treatment (Figure S5E, Table S3). However, these observations are different from the previous results and mechanism reported by Hrabárová et al. and Chen et al.^23,27^ It was suggested that there is an involvement of a Weissberger’s oxidation system involving the reactive species generated from H_2_O_2_, metal ions, and ascorbate toward HA degradation with the oxidation of the glucuronate residues. Moreover, these modifications were not accompanied by any changes in the FTIR spectrum of the native HA. However, as the current systems lack the presence of any metal ions, there may be a different mechanism for HA modification and the further degradation induced by the reactive species resulting from H_2_O_2_ degradation, but this is outside the scope of the current study. Moreover, no significant differences were observed before and after HA treatment with hyaluronidase (Figure S8F, Table S3), referring to a regular fragmentation of HA through the enzymatic action causing hydrolysis of the β-1,4-glycosidic bond, which supports the ^1^H-NMR findings.

#### 3.3.2 HA Fragmentation Following Different Treatments

**Figure 8:**
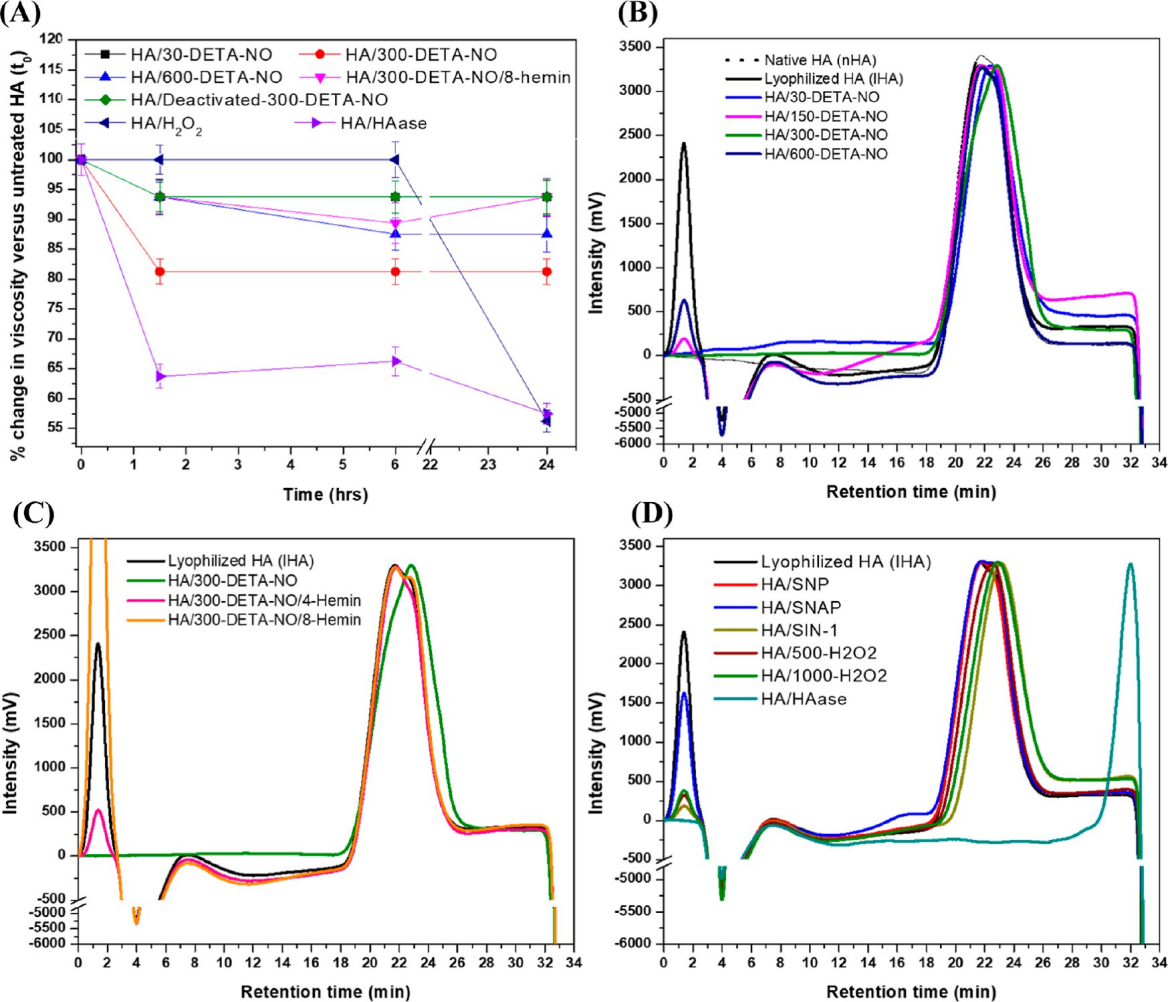
HA fragmentation following different treatments. (A) Change in viscosity calculated over time of 1000 kDa HA following treatment with 30, 300, and 600 μM DETA-NO; a mixture of 300 μM DETA-NO and 8 μM hemin; deactivated 300 μM DETA-NO; 1000 μM H_2_O_2_; or 100 U/mL hyaluronidase. At time 0, the initial viscosity of 1000 μg/mL HA in PBS was measured, followed by the different treatments and incubation at 37 °C for 24 h when calculating the viscosity of each solution after 1.5, 6, and 24 h. Data are represented as mean ± SD, *n* = 3. (B) Comparison of the HPLC-RID chromatograms of nHA, unmodified lHA, HA/30-DETA-NO, HA/150-DETA-NO, HA/300-DETA-NO, and HA/600-DETA-NO. (C) Comparison of the chromatograms of nHA, lHA, HA/300-DETA-NO, HA/300-DETA-NO/4-Hemin, and HA/300-DETA-NO/8-Hemin. (D) Comparison of the chromatograms of lHA, HA/SNP, HA/SNAP, HA/SIN-1, HA/500-H_2_O_2_, HA/1000-H_2_O_2_, and HA/HAase. Following the incubation of each mixture in PBS for 24 h at 37 °C, it was lyophilized, dissolved in HPLC grade water for a final concentration of 500 μg/mL, and separated by the HPLC system and detected by RID. The description of each HA product is given in Table 1.

The dynamic viscosity of the HA solutions under different treatments was monitored over time, and % of change from the untreated HA at a shear rate of 52 1/s is shown in the Figure 8A, while the actual viscosity values measured over time within the shear rate of 0.01–100 1/s are shown in Figure S9A–G. A slight decrease in the viscosity was observed after 1.5 h of mixing HA with both 30 μM and deactivated 300 μM DETA-NO (Figure S9A,E). However, a more significant decrease in viscosity was found in the case of HA/300-DETA-NO and HA/600-DETA-NO, with a higher % of the change in the former case after 24 h of incubation (Figure S9B,C), relating to the initiation of HA depolymerization/fragmentation in the presence of ^•^NO.

However, the addition of hemin to the HA/DETA–NO mixture caused the partial viscosity of HA to be maintained, in contrast to the HA/300-DETA-NO (Figures S9D). This suggests that by scavenging of ^•^NO, its inducing effects for HA degradation can be modulated. Moreover, although no changes in the viscosity of HA were observed after mixing with 1000 μM H_2_O_2_ over the first 6 h of incubation, a drop in viscosity was observed after 24 h (Figures S9E). Furthermore, hyaluronidase caused a rapid decline in the viscosity of the HA solution after 1.5 h of incubation, which continued over time as an indicator of the enzymatic degradation of HA (Figure S9F). These viscosity measurements give an overview of the possible degradation of HA once incubated with ^•^NO, ROS, and hyaluronidase and help understand the results of the other characterization techniques. However, bearing in mind the generation of radicals via the action of the different ROS/RNS in the systems involved, possible chain-termination reactions can occur during the incubation period and affect the viscosity measurements (Lurie et al.).^59^ Moreover, for comparison, the values measured for HA of different MWs are shown in Figure S9H.

RID is a commonly used detector for the isocratic LC separation of multiple polymers, with various advantages, including its ease of use and sensitivity to the polymer concentration.^60^ The *R*_t_ values calculated from the chromatograms of HA with different MWs showed that the longest time was observed in the case of the lowest-MW HA, 16 kDa, at 28.74 min, while 1500 kDa showed the shortest *R*_t_ (20.56 min), which was different by only 1.203 min from the 1000 kDa HA (*R*_t_ = 21.763 min). Moreover, a difference in *R*_t_ of only 0.287 min was observed between 700 kDa HA (*R*_t_ = 22.05 min) and 1000 kDa HA, with the elution of 500 kDa HA detected after 23 min. Comparing these standard results with the chromatograms of the HA products, the slight changes in the *R*_t_ value of HA following treatment with ROS or RNS may be understood to result from a significant difference in the MW of the polymer. When comparing the *R*_t_ of lHA and nHA, similar retention times were observed, but these were long after the incubation of HA with 300 μM DETA-NO (*R*_t_ = 22.36 min), with only slight changes in the case of the other concentrations of DETA-NO (Figure 8B). The difference in *R*_t_ of 0.683 min between lHA (*R*_t_ = 21.677 min) and HA/300-DETA-NO may indicate partial depolymerization between 1000 kDa HA and 700 kDa or 500 kDa HA. These observations may relate to either side reactions of ^•^NO, especially at its higher fluxes generating side NO congeners such as N_2_O_3_, or a controlled reactivity of HA against the released ^•^NO, with possible modification of the HA structure without inducing a change in its MW or viscosity, and this has been explored using ^1^H-NMR and FTIR.

However, with the addition of hemin to HA/DETA-NO mixtures, as in the case of HA/300-DETA-NO/4-Hemin and HA/300-DETA-NO/8-Hemin, this shifting in *R*_t_ vanished without any differences from the non-modified HA (Figure 8C). By comparing the effects of the same concentrations of DETA-NO, SNP, SNAP, and SIN-1, only the peroxynitrite donor showed an *R*_t_ at 23.14 min, with a difference in *R*_t_ of 1.463 min, and it was similar to that of H_2_O_2_, relating to a decrease in the MW of HA following the treatment (Figure 8D). H_2_O_2_ has a reported efficiency to cause a degradation of HA at specific concentrations in the presence of metallic ions.^23,27^ Furthermore, as SIN-1 is considered a donor of both ^•^NO and O_2_^•–^, this can explain the decrease in the MW of the resulting HA. Moreover, as expected, hyaluronidase caused the most significant change in MW of HA (*R*_t_ = 31.99 min), reflecting its catalytic degradation effects.

The change in the degree of polymerization of HA following the different treatments was monitored by agarose gel electrophoresis, where the loaded HA chains migrate through the gel based on the MW. The faster migration accompanies the shorter HA chains/fragments, while the longer the HA chains, the slower their migration. The first four bands to the left of the gel shown in Figure S10A are the control bands, including native unmodified 500, 700, and 1000 kDa HA and unmodified but lyophilized 1000 kDa HA, followed by the different HA products. Moreover, Figure S10B shows the optical density (OD) calculations for each separated band. Table 3 summarizes the distance migrated by each HA sample from the loading point at the edge of each well to the point with the maximum density in each separated band. 500 kDa showed the fastest migration, followed by 700 kDa, while the lyophilized and non-lyophilized 1000 kDa HA showed the lowest distance of migration without any differences between each other. The HA products from HA/DETA-NO systems showed different migration abilities, where the migration of HA/30-DETA-NO was not different from that of lHA, followed by a slight enhancement in the case of HA/600-DETA-NO, while HA/300-DETA-NO showed the fastest migration. These results relate to certain fragmentation/degradation levels of HA once treated with DETA-NO, which depend on the concentration of the released ^•^NO, where the lowest DETA-NO concentration did not cause any effects, while 300 μM was the one that produced the most significant effect. However, although the ^•^NO levels from 600 μM are the highest among the tested DETA-NOs, there is still a high probability of self-association of ^•^NO molecules producing the stable N_2_O_3_ molecules, which may be the reason for the lower migration rate at this concentration than in the case of HA/300-DETA-NO. Interestingly, the effects of 300-DETA-NO were negated in the presence of hemin, where the HA/300-DETA-NO/hemin products showed migration levels similar to those of the unmodified lHA. These results support the hypothesis that hemin will function as a ^•^NO-scavenging compound, further protecting HA from the fragmentation reactions.

**Table 3:** Calculated Relative Mobility of HA Chains through the Agarose Gel Following the Electrophoresis of Unmodified HA with Different MWs, Lyophilized Untreated 1000 kDa HA, HA/30-DETA-NO, HA/300-DETA-NO, HA/600-DETA-NO, HA/300-DETA-NO/4-Hemin, HA/300-DETA-NO/8-Hemin, HA/SIN-1, HA/1000-H_2_O_2_, and HA/HAase^a^

group			distance of migration from the loading point (cm)
untreated HA (kDa)	500		3.94 ± 0.33
	700		2.24 ± 0.25
	1000	Untreated	1.70 ± 0.20
		Lyophilized	1.70 ± 0.24
HA/30-DETA-NO			1.70 ± 0.18
HA/300-DETA-NO			2.26 ± 0.30
HA/600-DETA-NO			1.73 ± 0.25
HA/300-DETA-NO/4-Hemin			1.73 ± 0.20
HA/300-DETA-NO/8-Hemin			1.70 ± 0.18
HA/1000-H_2_O_2_			3.02 ± 0.28
HA/SIN-1			3.58 ± 0.30
HA/HAase			N/A

a Following the scanning of the electrophoresis blot, the distance of migration from the well (loading point) to the point with the highest OD in each band was measured using ImageJ software. Data are represented as mean ± SD, *n* = 3. The description of each HA product is illustrated in Table 1.

Furthermore, H_2_O_2_ and SIN-1 caused fragmentation of HA, explained by the faster migration levels. These results confirm the efficiencies of ^•^NO at specific concentrations to cause partial depolymerization of the HA chains, which are comparable to the already reported effects of ROS generated from H_2_O_2_^23,27^ and of ONOO^•–^, resulting from SIN-1.^45^ Moreover, no bands were detected in the case of HA/HAase, suggesting a high level of HA degradation, and the resulting fragments could not be detected, especially as this technique is not sensitive for the HA chains with MWs less than 25 kDa. These observations, alongside the HPLC results, confirm the partial degradation/depolymerization of HA under the different treatments. Moreover, considering the changes within the molecular structure of HA under some of them, the HA fragmentation may result from partial oxidation reactions on specific positions on the glycosidic rings causing degradation of the O-glycosidic bonds or may be due to the enzymatic action directly breaking these bonds.

### 3.4 Mechanism of Reaction

**Scheme 2:**
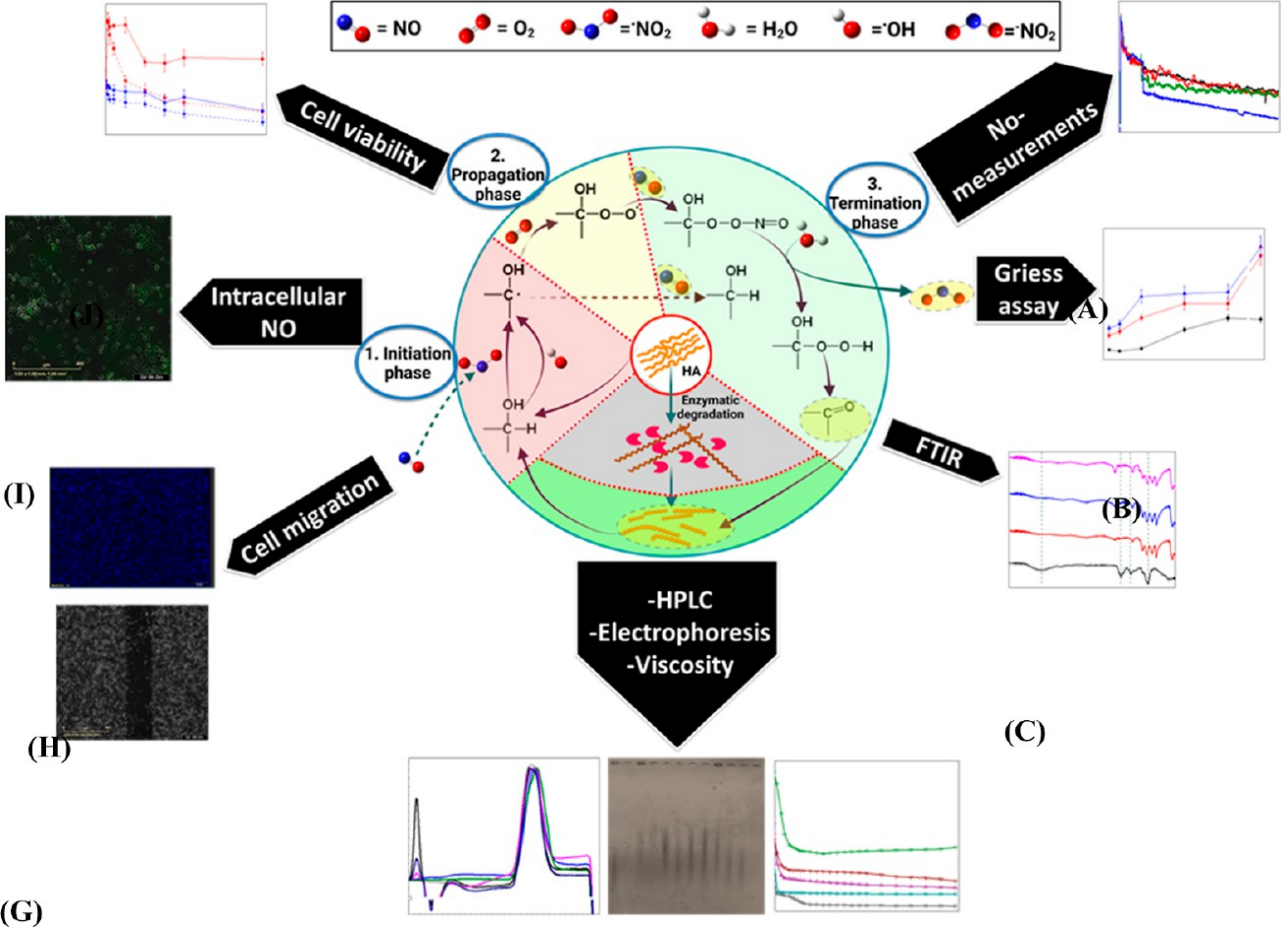
Cascade of the Main Extracellular Radical Reactions Involved in the Degradation of HA and How This Was Evaluated The reaction starts with (1) the initiation phase in which either ^•^OH or ^•^NO_2_, produced from the reaction of ^•^NO with molecular oxygen, attacks the C atoms forming the main rings of the monomer units, followed by (2) the propagation phase in the presence of O_2_. The reaction is terminated by steps within (3) the termination phase, starting with ^•^NO, leading finally to a partial fragmentation of the HA chains, which can either close the catalytic cycle or start a new initiation phase. These radical-induced reactions are compared with the hyaluronidase-catalyzed HA degradation. Schematic created with BioRender.com.

From the previous results, the mechanism of HA modification with the subsequent depolymerization can be understood, especially as various positions on the monomer units are prone to oxidation, mainly at the carbon atoms bonded to −OH groups. Scheme 2 summarizes the main phases involved in the interaction of HA with ^•^NO and HAase-induced HA degradation. The alcohols at the C6 atoms will be oxidized to either the −CHO or −COOH group, but the corresponding chemical shifts were not detected in the ^1^H-NMR spectra. Although ^•^NO is a reactive free radical, it cannot abstract any allylic or tertiary H atoms from organic molecules at room temperature, where the H–NO bond with a strength of 208.6 ± 2.1 kJ/mol is weaker than the C–H bond in the organic compound.^61^ Accordingly, the possible reactions of ^•^NO with the monomer units of HA can be through binding with already generated free radicals on one of the previously mentioned reactive positions, with a mechanism similar to that of ^•^NO reacting with both lipid peroxyl and alcohol peroxyl radicals. In the case of SIN-1, the released ^•^NO and superoxide anion (O_2_^•–^) react together, producing ONOO^•–^ through a second-order reaction (reaction 2; *K*_eq_ = 6.7 × 10^9^ M^–1^·s^–1^).^62^ According to the observations of Al-Assaf et al. and Stankovská et al., the interactions between HA and ONOO^•–^ are not direct but take place via certain intermediates, starting with its protonation into ONOOH, which decays via a first-order reaction (*K*_eq_ = 1.15 s^–1^) producing ^•^OH and nitrogen dioxide (^•^NO_2_).^25,26^ The ^•^OH start the process of hydrogen abstraction from HA, where Lurie et al., 2003 suggested a mechanism for the formation of peroxyl radicals on the C atoms of the monomer rings in the HA backbone.^59^ Here, the reaction initiates with the abstraction of H atoms from the −OH groups on these C atoms via various ROS species in solution, forming C-centered macroradicals (reaction 4). The estimated rate constant of reactions of ethanol and propanol with ^•^OH was 1.9 × 10^9^ M^–1^·s^–1^^63^
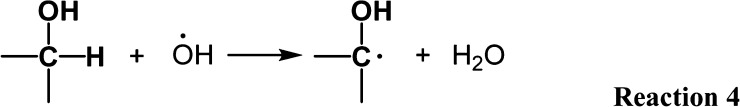
4

However, in the case of ^•^NO donors, ^•^NO and O_2_ combine slowly, forming ^•^NO_2_ (reaction 5; *K*_eq_ = 2 × 10^6^ M^–2^·s^–1^)^64^
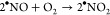
5

^•^NO_2_ is a one-electron oxidant, which can abstract H atoms from HA, similar to the functionality of ^•^OH radicals (reaction 6)
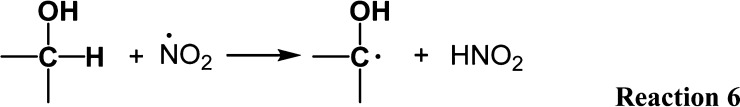
6

In the propagation phase, a peroxy-macroradical is formed in the presence of molecular oxygen (reaction 7), which may decay via the interactions with an active free-radical species. The estimated rate constant of the oxygenation reaction of the 1-hydroxy-1-methylethyl radical, having the closest structure to that of the free radical on the C atom of the ring, was 4.2 × 10^9^ M^–1^·s^–1^, while it reached 2.9 × 10^9^ M^–1^·s^–1^ in the case of reacting oxygen with the glucose radical^63^
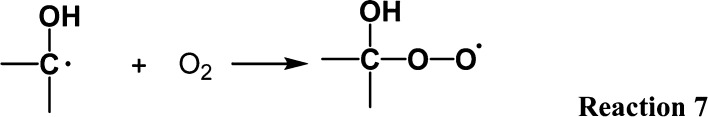
7

Considering ^•^NO release from DETA-NO, it will interact with the peroxy-macroradical forming organic peroxynitrite (reaction 8) as an intermediate (*K*_eq_ = 2 × 10^9^ M^–1^·s^–1^)^65^
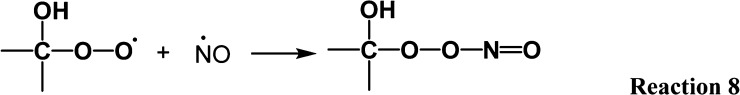
8

The latter species will then be hydrolyzed, generating nitrite (NO_2_^–^) and a hydroperoxide group connected to the monomer ring (reaction 9), which then decomposes into a stable keto form (reaction 9) accompanied by the cleavage of the O-glycosidic bond, especially when the reaction takes place at the anomeric carbon atom, as reported previously.^23^ Furthermore, disproportionation of the peroxyl radicals was also suggested as one of the reactions leading to the generation of stable keto-forms^66^
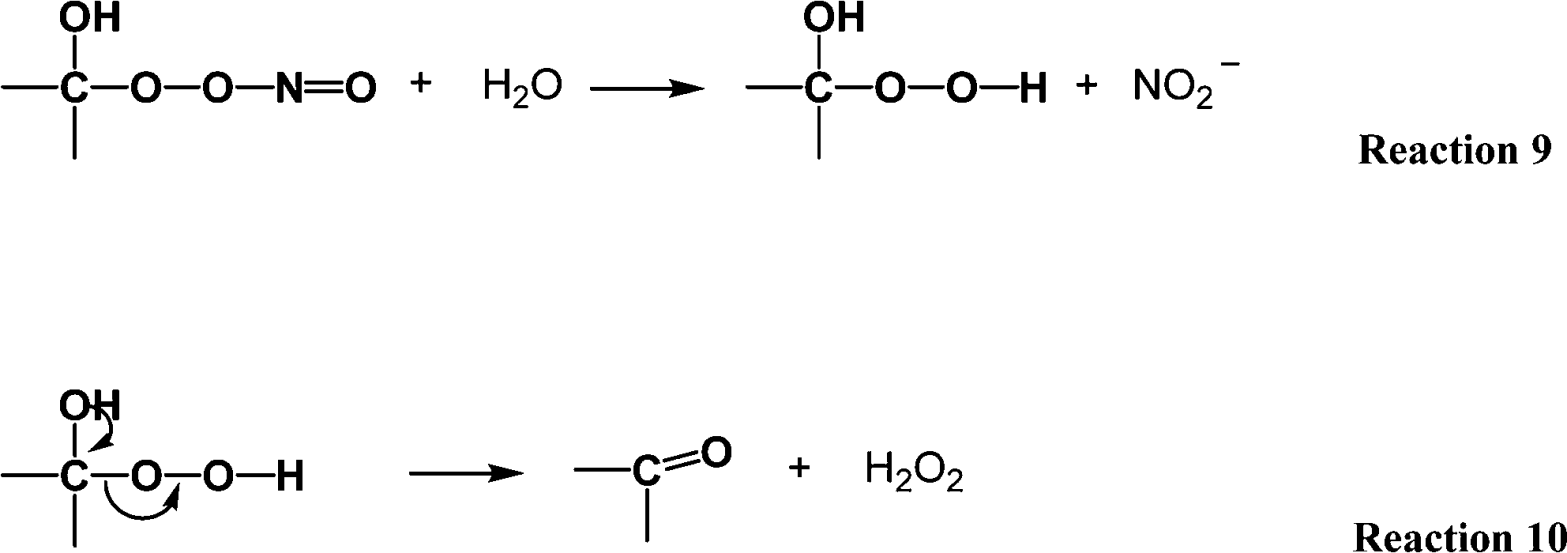
9

Following the treatment of HA with DETA-NO, the increase in nitrite concentration, alongside the detected new band at 1745.59 cm^–1^ (C=O stretching) in the FTIR spectrum (Figure 6), supports this mechanism. Moreover, the unstable organic peroxynitrite can also decompose into the alkoxyl radical (RO^•^) and ^•^NO_2_ species in the form of a caged radical pair (reaction 11; *K*_eq_ = 0.1 to 0.3 s^–1^),^65,67^ where the former can interact with ^•^NO forming an alkyl nitrite (RNO_2_) and the reaction is terminated
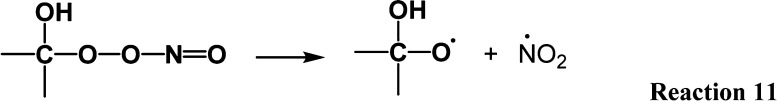
11

Furthermore, another possibility still exists for ^•^NO’s reaction with the radical species from reaction 1, causing its neutralization and producing NO_2_^–^ ions; however, this pathway will not generate oxidized rings (reaction 12). A similar mechanism was proposed during the reaction between ^•^NO and oxidized α-tocopherol^68^
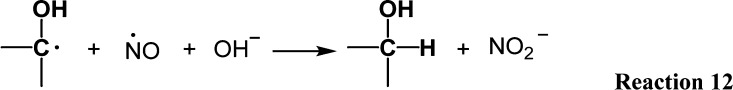
12

In addition, another possibility remains concerning the self-reaction of the radical organic species and the peroxyl radicals, which can also compete with their reactions with ^•^NO. However, the later species seem to have higher reactivity due to the steric hindrance of polymer chains in the former case.

Additionally, ^•^NO_2_ can react further with excess ^•^NO forming N_2_O_3_, considered a NO^+^ donor via transfer of NO^+^ not via its release. N_2_O_3_ can also be produced from the reaction of an excess of ^•^NO with O_2_ (*K*_eq_ = 8 × 10^6^ M^–2^·s^–1^).^45^ However, in this case, N_2_O_3_ will function as a nitrosating agent,^69^ mainly targeting the thiols. These primary and secondary amines are groups not available in the structure of HA as described by Duan and Kasper.^70^ Accordingly, the potential groups for the oxidation reactions are at the monomeric units’ C2, C3, and C4 positions. The latter position is responsible for the further degradation of the polymer chain once attacked by the radicals. Furthermore, in the cellular systems, various active oxygen species are generally produced, which can initiate a reaction similar to that of the ^•^OH radicals.

### 3.5 In Vitro Study

#### 3.5.1 Cytocompatibility of nHA and Lyophilized HA Products and Effects on Cell Adhesion

**Figure 9:**
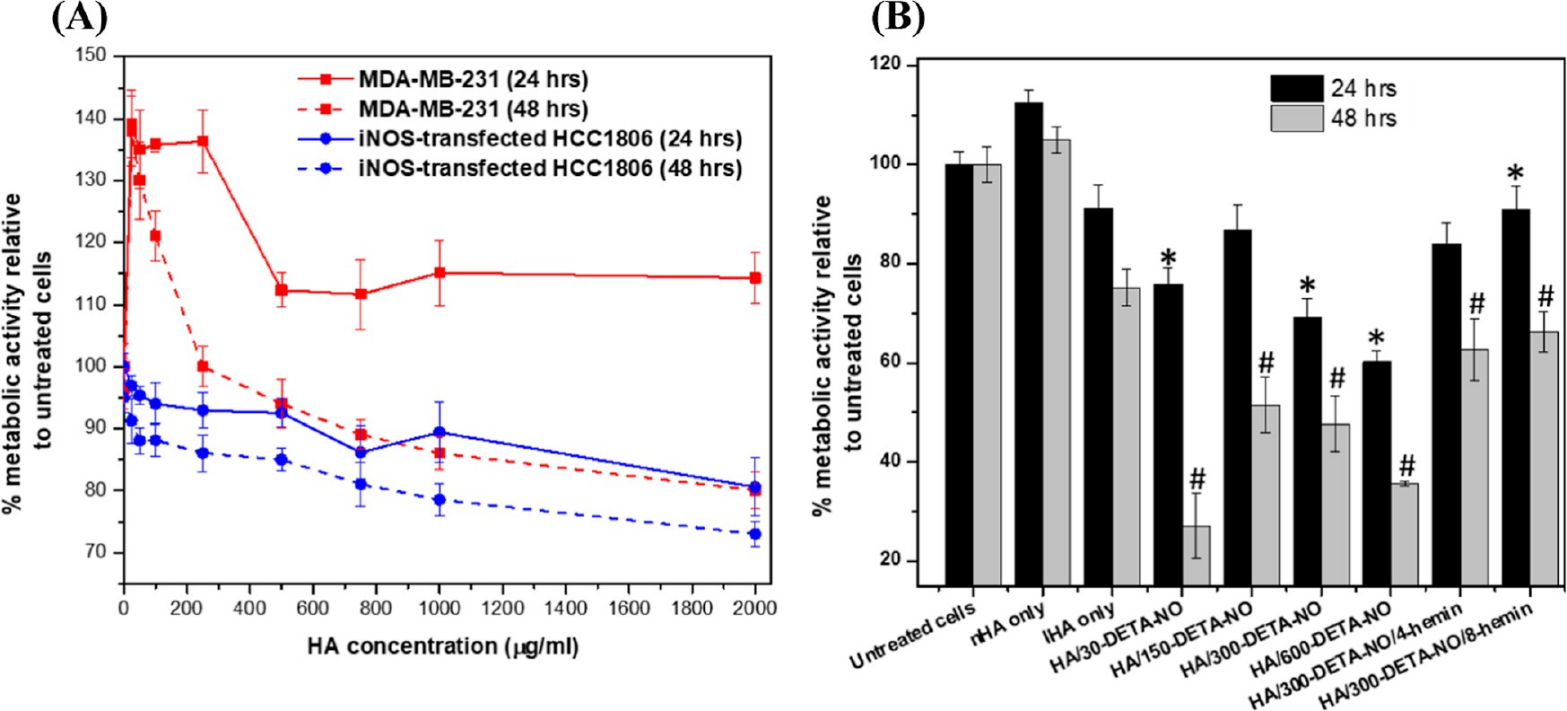
In vitro cytotoxicity of 1000 kDa nHA and HA products. (A) Metabolic activity of MDA-MB-231 (red color) and iNOS-transfected HCC1806 (blue color) against different concentrations of 1000 kDa HA (nHA) after 24 and 48 h of culture. (B) Metabolic activity of MDA-MB-231 after 24 (black color) and 48 h (gray color) of culture with nHA, untreated lHA, HA/30-DETA-NO, HA/300-DETA-NO, HA/600-DETA-NO, HA/300-DETA-NO/4-hemin, and HA/300-DETA-NO/8-hemin. Each HA product was diluted in FBS-free RPMI to a final concentration of 500 μg/mL, added to the cells cultured further at 37 °C in 5% CO_2._ The metabolic activity was measured using the alamarBlue assay. Data are represented as mean ± SD, *n* = 3. *^,#^, *P* < 0.05 versus the cells treated with lHA only for 24 and 48 h, respectively, using a two-tailed unpaired Student *t*-test. A description of each HA product is presented in Table 1. The effects of different concentrations of DETA-NO on the metabolic activity of MDA-MB-231 were evaluated before.^37^ To assess the possibility of the remaining NO donor or H_2_O_2_ in the lyophilized HA products, these were dissolved in PBS containing 1 mM luminol, and the luminescence readings were measured over time. This was to find whether these products contain certain components which can enhance the luminescence intensity, such as ROS or RNS. An example of the results after 50 min of incubation at 37 °C is shown in Figure S12. There is still a probability that non-degraded DETA-NO is present in the lyophilized HA products, which enhanced the luminescence signal upon dissolving HA in PBS. However, this does not explain the sharp decrease observed in metabolic activity of cells in HA/30-DETA-NO compared to the other HA/DETA-NO products. Similarly, although the HA/H_2_O_2_ products did not enhance the luminescence signal, these products strongly inhibited cell viability. Accordingly, the observed effects of the different HA products are due to the modified HA chains compared to the released radicals in solution.

1000 kDa HA at all tested concentrations was cytocompatible with the MDA-MB-231 cells. The 25, 50, 100, and 250 μg/mL concentrations significantly enhanced the metabolic activity above 130% after 24 h of culture (Figure 9A). Moreover, the higher concentrations showed similar effects on the metabolic activity reaching around 110%, with a general decrease after 48 h of culture, but maintained at above 80% at all tested concentrations. However, in the case of iNOS-transfected cells, a lower metabolic activity was observed at all tested concentrations after 24 h of culturing, with the concentration of 2000 μg/mL causing the most significant decrease. In addition, a further decrease in metabolic activity was detected after 48 h, but to levels different from those observed in MDA-MB-231 cells relating to the different behavior of cells toward HA. As reported before, hyaluronan, via its main cellular receptor, CD44 isoforms, regulates the proliferation of cancer cells by certain reported mechanisms.^71−73^ HA, therefore, could block the selective binding of certain nanoparticles targeting the activated CD44 in cancer cells, as reported previously.^74,75^ HA at specific concentrations enhanced the metabolic activity of the tested cells, which mainly relates to these reported mechanisms. However, a possible change in the accompanying signals may take place at the higher concentrations, possibly due to the complete blocking of the CD44 isoforms, and this effect is cell line-dependent. Moreover, Gedikli et al. reported different cytotoxic effects of HA on different cell lines, dependent on the concentration.^76^ However, the exact mechanism is still under debate, and we are currently developing our understanding of how HA interferes with the metabolic pathways within the tested MDA-MB-231 and HCC1806 cells.

As the size of HA is one of the determining factors for the interactions between HA and the cell receptors, including CD44, RHAMM, and TLRs,^77,78^ the effects of the products from HA reactions were evaluated first by measuring the metabolic activity of MDA-MB-231 cells. lHA without prior modification was more cytotoxic than nHA, suggesting possible modification/fragmentation in the polymer structure during the freeze-drying process. Accordingly, the statistical analysis of these data was performed and compared to the results in the case of lHA only. The different HA products showed decreased metabolic activity after 24 h of culture, which was more significant after 48 h than the native HA (Figure 9B). This may relate to the partial fragmentation of HA and the possible cytotoxic effects of low-MW-HA chains. In addition, the metabolic activity of cells treated with HA/300-DETA-NO reached 69.12 ± 3.87 and 47.66 ± 5.62% after 24 and 48 h, respectively; the HA products from HA/DETA-NO with 4 and 8 μM hemin showed enhancement in the metabolic activity. Here, it was 84.02 ± 4.25 and 62.59 ± 6.25 after 24 and 48 h of cell culture with HA/300-DETA-NO/4-hemin, respectively, while it reached 90.85 ± 4.7 and 66.28 ± 4.08 at the same time points in the case of HA/300-DETA-NO/8-hemin. Considering the possible cytotoxic effects of HA fragments following their treatment with DETA-NO, the scavenging of ^•^NO by hemin would protect it, and the effects of the final HA products will be expected to have higher cytocompatibility.

Moreover, although the HA/SNP and HA/SNAP products did not significantly affect the metabolic activity compared to those of lHA, the HA/H_2_O_2_ products only caused the most significant drop in the metabolic activity, similar to that elicited by DMSO (Figure S11). The effects of HA on the viability of MDA-MB-231 were found to be MW-dependent as reported previously,^79^ with 200 kDa HA being less cytocompatible with MDA-MB-231 cells than the HA with an MW less than 10 kDa. Moreover, the binding of HA oligomers to the CD44 and TLR4 receptors was MW-dependent. It was also found that the high binding energies of these short chains are accompanied by less cell viability.^80^ These results can explain the observed metabolic activity of MDA-MB-231 cells incubated with the HA products; however, each lyophilized product is a mixture of chains of different MWs, and the exact effect of each fragment cannot be studied under the current experimental conditions.

As a part of HA in the ECM exists as an immobilized fraction via its interactions with the other ECM components, the attachment of cells to wells pre-coated with HA was studied, where the immobilization was performed via physical adsorption of the HA layers to the well. Although the culturing of cells with DETA-NO did not induce their adhesion significantly in the absence of any coating, the attachment was improved considerably once the wells were coated with either BSA or HA, with the adhesion rate dependent on HA concentration (Figure 13A). The effects of ^•^NO only were similar to those observed by Lahiri and Martin, 2009, when ^•^NO was released from SNP, and the effects on cell adhesion depended on the incubation period.^81^ It has to be pointed out that the tested DETA-NO concentration, 300 μM, was not toxic to the cells. The % of cell adhesion is shown in Figure 13A, while Figure S14 compares the distribution of cell counts in all acquired spots within each group. While the cell adhesion to BSA-coated wells was significantly improved in the presence of DETA-NO compared to that of the medium only, no significant changes were observed in the case of a well with immobilized 200 μg/mL HA. However, the adhesion decreased significantly on culturing of cells with ^•^NO to immobilized 500 μg/mL HA. Although the well coating with 1000 μg/mL HA significantly enhanced the cell attachment compared to the non-coated wells, the % of cell adhesion was lower than that of the other groups. In addition, the presence of ^•^NO decreased this percentage as well, but this was insignificant compared to the ^•^NO-free system. Herrera-Gayol and Jothy reported similar results for the effects of immobilized HA on cancer cell adhesion.^82^ Moreover, Tavianatou et al. showed that treating MDA-MB-231 cells with HA of different MWs up to 200 kDa enhanced their attachment to the collagen type I matrix.^79^ These observations, alongside the results presented, confirm the potential of the immobilized HA to alter cell behavior, mediated mainly by the binding of HA to the cell receptors. Moreover, these binding affinities can also be considered dependent on the MW, as described in Section 3.5.1.

The effects of ^•^NO on cell adhesion to microvessels are not consistent and depend on the cell type and the local ^•^NO concentrations, as summarized by Zhang et al.^83^ However, some studies found that the adhesion of the circulating tumor cells to the microvessels is preferred on the sites with high ^•^NO concentrations,^83^ which enhances the microvessel permeability.^84^ However, from the recent results (Figures S13A and S14), the role played by BSA and ^•^NO in the enhancement of cell attachment seems to be different when ^•^NO is added to the cultured cells on an HA-coated plate. Teriete et al. and Banerji et al. investigated the modes of interactions between CD44 and the polymeric chains of HA and how this binding regulates a series of downstream cellular effects.^85^ Moreover, Bhattacharya et al. found that both the deacetylation and sulfation of HA reduced its binding affinity to the CD44 receptor compared to nHA.^86^

Furthermore, following the coating of wells with HA/300-DETA-NO, produced after the treatment of HA with 300 μM DETA-NO for 2, 6, and 24 h and lyophilization, different attachment behaviors were observed (Figure S13B). For instance, the 2 h incubation of HA with DETA-NO did not lead to significant differences in cell attachment compared to untreated HA. At the same time, the more extended periods of HA treatment were accompanied by more inhibition of cell adhesion with insignificant differences in whether ^•^NO was added to the culture medium. Consequently, due to the binding affinity between the HA chains and cell receptors, the reaction of ^•^NO with HA chains, which is responsible for specific alterations in its chemical structure as illustrated in Sections 3.2 and 3.3, may cause some changes to these interactions and thereby affect the cell attachment. Moreover, the prior treatment of HA with ^•^NO, followed by lyophilization, was found to affect its further reactions to new amounts of ^•^NO.

#### 3.5.2 Effects of HA and HA Products on the Intracellular ^•^NO

**Figure 10:**
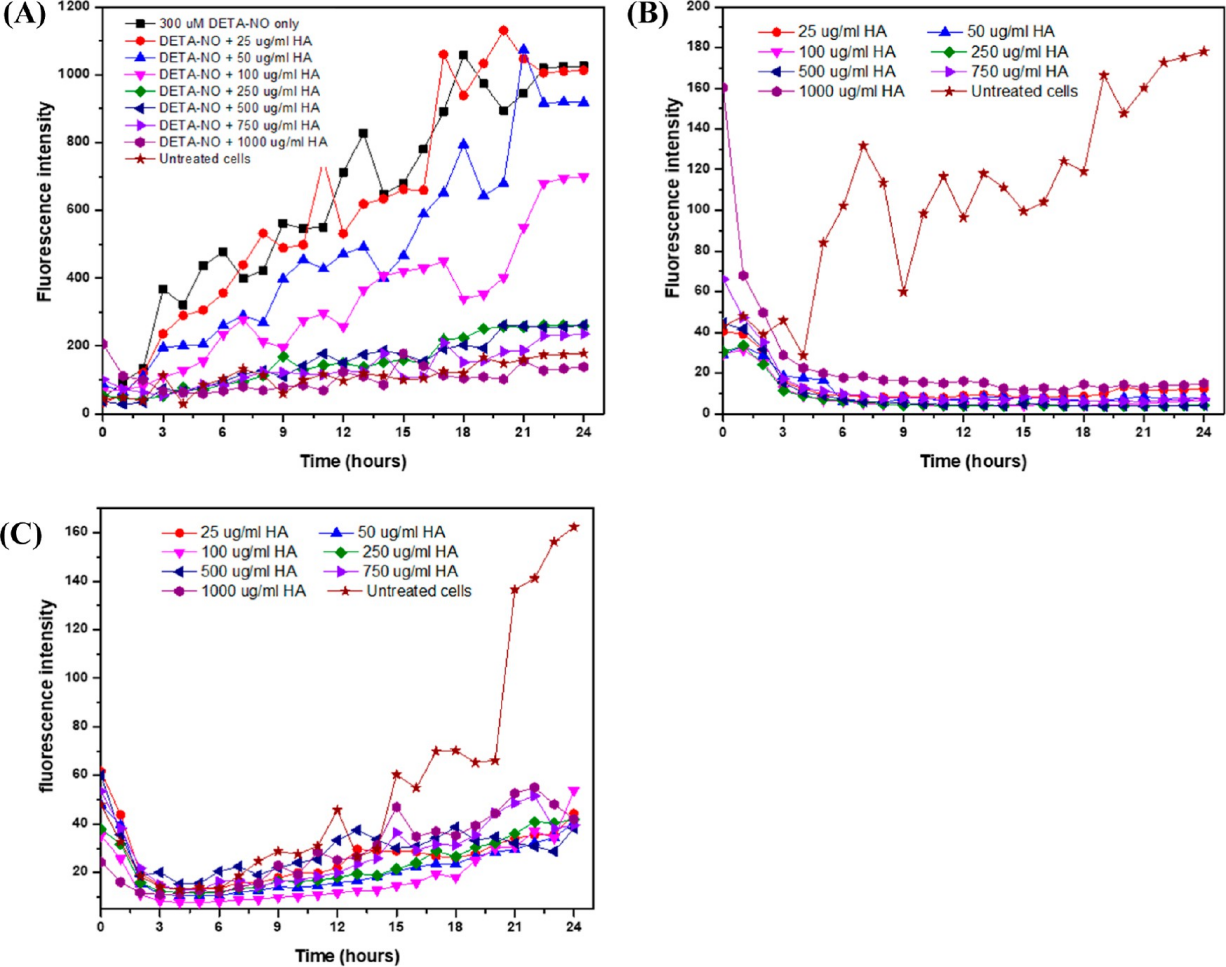
Kinetics of the changes in intracellular ^•^NO levels revealed by the ^•^NO-specific indicator DAF-FM-DA and its fluorescence in MDA-MB-231 cells in response to 300 μM DETA-NO in the presence of different concentrations of 1000 kDa HA (A) and the typical fluorescence in the presence of HA without a ^•^NO donor (B) and iNOS-transfected HCC1806 cells in response to the treatment with different concentrations of HA (C). The cells were treated with DAF-FM-DA for 1 h and then photographed after adding the different treatments using the real-time Incucyte imaging system (phase contrast and green fluorescence signals). Data are represented as the mean of readings of three samples per group.

DAF-FM diacetate was employed as a probe for detection of the intracellular ^•^NO, where it diffuses through the cellular membranes and, under the action of intracellular esterases, converts to DAF-FM as the primary probe for ^•^NO.^87^ However, the detection does not rely on the reaction of DAF-FM with ^•^NO itself but its oxidation products, particularly N_2_O_3_ and ^•^NO_2_, causing an enhancement in the fluorescence intensity.^88,89^ The Incucyte system was employed to analyze the fluorescence images acquired over time and generate data graphics. In the case of MDA-MB-231 cells, the intracellular ^•^NO levels, expressed in terms of the intensity of the green fluorescence, were found to enhance following culturing with 300 μM DETA-NO only compared to the untreated cells. However, in the presence of HA, a decrease in the fluorescence was observed, which was proportional to the HA concentration. For instance, the lowest tested concentration, 25 μg/mL, showed no statistically significant differences from the DETA-NO only group. Still, the higher concentrations caused a gradual decrease in the fluorescence intensity, with the concentrations of 750 and 1000 μg/mL showing insignificant differences from the untreated cells (Figure 10A).

To compare these results with the ^•^NO-free systems, the intensity of fluorescence of MDA-MB-231 cells was studied following the treatment with the same HA concentrations as before without the ^•^NO source. It can be observed that the fluorescence intensity was sharply inhibited, with no significant differences between the different HA concentrations, mainly after 4 h of culturing (Figure 10B), raising the possibility of side reactions of HA with DAF-FM or its active form, leading to an inhibition of its fluorescence. However, in the presence of ^•^NO, the enhanced fluorescence signal indicates an overcoming of these interferences. Although there is still a probability of interference of HA with the DAF-FM-related fluorescence, this would be expected to require specific signaling pathways for the final inhibition of the fluorescence intensity. Accordingly, the main changes in the fluorescence signal relate to the interactions of ^•^NO, N_2_O_3_, and ^•^NO_2_ with DAF-FM.

Furthermore, by comparing the fluorescence intensity once the cells have been treated with 25, 50, and 100 μg/mL in the presence and absence of DETA-NO, the functionality of the NO probe and the specificity of the signal toward ^•^NO can be verified, and these results confirm that HA itself does not interfere with its fluorescence. Accordingly, these results relate to the efficiency of HA to scavenge the extracellular ^•^NO, and therefore, its intracellular levels decrease, with the concentrations above 50 μg/mL having a significant efficiency against the increased ^•^NO levels.

Generally, lyophilized HA products from the HA/DETA-NO groups, HA/SNP and HA/SNAP, showed a similar affinity in decreasing the intracellular ^•^NO induced by 300 μM DETA-NO with the lHA and nHA showing lower efficiencies (Figure S15A). Furthermore, the HA/DETA-NO/hemin products did not differ from the HA/DETA-NO products (Figure S15B). Moreover, HA/SIN-1 and HA/1000-H_2_O_2_ had the lowest efficiency to scavenge ^•^NO compared to the other products (Figure S15C). These results indicate that the interactions of the HA fragments with ^•^NO are governed by its MW and the mechanism by which the ROS/RNS induces its depolymerization, including how they modify the structure of HA alongside the termination of the radical reaction. Moreover, HA is a polyelectrolyte whose physico-chemical properties depend on its MW; the long chains of HA will have higher stability in the random coiled formation, while the short chains will be more extended.^6^ Accordingly, both the nHA and lHA will be less exposed to the ^•^NO released in the medium than the chains of the HA products.

To evaluate the effects of HA on the levels of ^•^NO produced intracellularly, iNOS-HCC1806-transfected cells were employed, and no ^•^NO donor was used. The kinetics of change in fluorescence levels in the case of iNOS-transfected cells was similar to that of MDA-MB-231 in the absence of the ^•^NO donor, particularly after 8 h of culture (Figure 10C). Although these cells were transfected to produce more ^•^NO, there were no significant differences in the accompanying fluorescence signal compared to the untransfected MDA-MB-231 cells. Moreover, the changes in fluorescence were similar in the case of the different HA concentrations for up to 8 h, followed by significant differences, with the low HA concentrations having the highest affinity toward the decrease of the intracellular ^•^NO levels. For instance, the 100 and 1000 μg/mL HA concentrations showed the lowest and highest fluorescence levels, respectively, compared to the other HA concentrations. The higher efficiency to quench the intracellular ^•^NO and its accompanied fluorescence in the case of the low HA concentrations refer to easier penetration inside the cells. However, the steric hindrance due to the accumulated HA chains may affect their ability to penetrate the cells, and accordingly, showed a lower efficiency in scavenging ^•^NO.

#### 3.5.3 Effects of HA and HA Products on the ^•^NO-Induced Cell Migration

**Figure 11:**
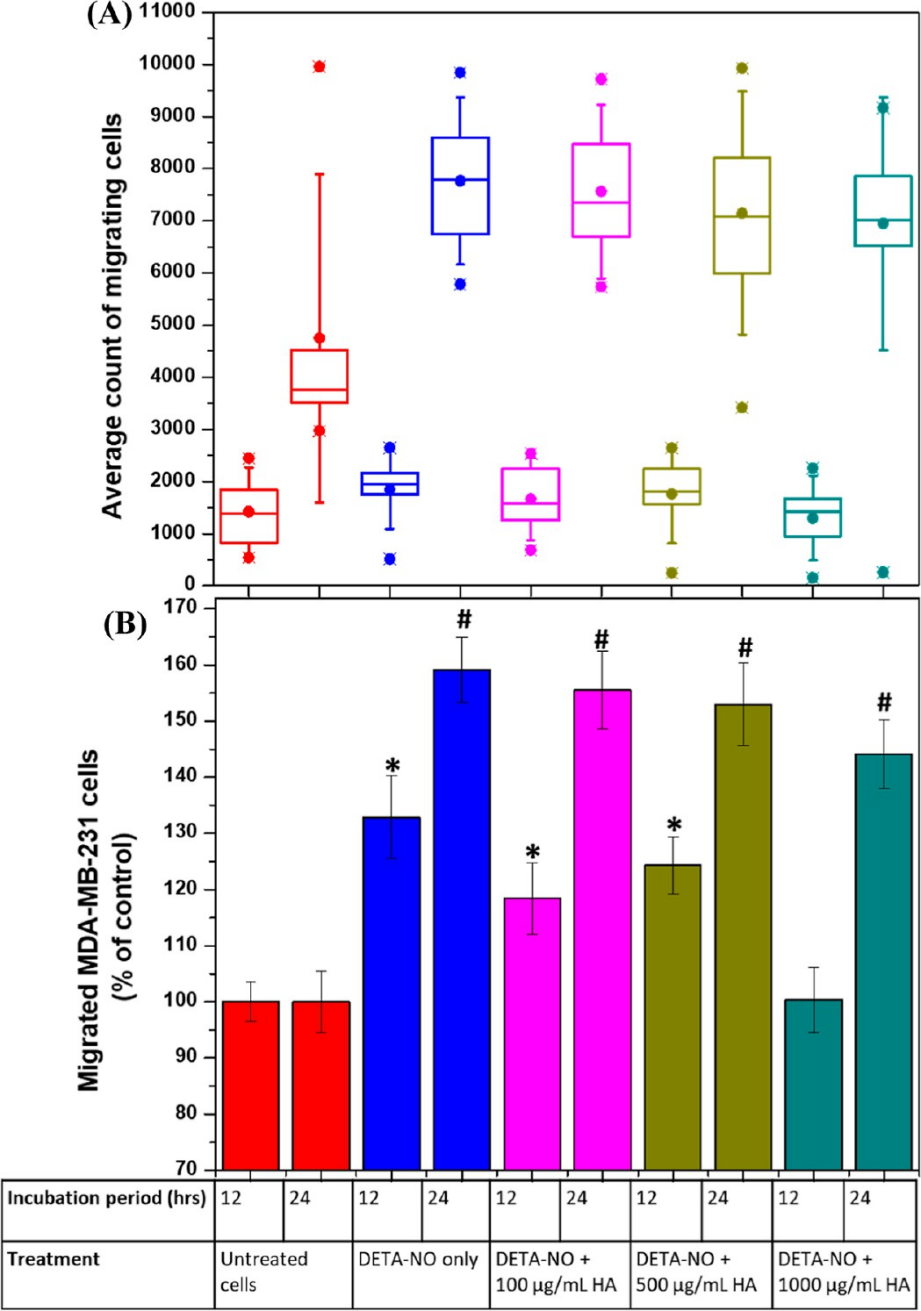
Effects of DETA-NO and 1000 kDa HA on the migration of MDA-MB-231 cells. (A) Box-whisker blot showing the distribution of the number of counted cells migrated through the transwell membranes toward the chemoattractant composed of 300 μM DETA-NO in FBS-containing RPMI in the presence or absence of 100, 500, and 1000 μg/mL HA. The whiskers represent the SD values. (B) % of migrated cells normalized to the count in the control group (untreated cells migrated toward the medium only). Following culture for 12 and 24 h, the number of migrated cells was counted. Data are represented as mean ± SD, *n* = 3. *^,#^, *P* < 0.05 versus the untreated cells (negative control) left to migrate for 12 and 24 h, respectively, using a two-tailed unpaired Student *t*-test.

The migration of cells through the transwell membrane requires them to sense a gradient of the chemoattractant, which in turn leads to their polarization. DETA-NO served as the donor of ^•^NO due to its long half-life reaching 20 h at 37 °C^36^ and so allows the investigation of ^•^NO’s effects over a long period of incubation. The effects of ^•^NO on cell migration and tumor proliferation and whether it has pro- or anti-tumorigenic effects depend on the concentration of ^•^NO and its flux and the tumor microenvironment.^29,90^ A comparison of the average count of migrated cells after 12 and 24 h of culturing of MDA-MB-231 with the different treatments is shown in Figure 11A,B, with more details on the cell count distribution shown in the Figure S16. Generally, the number of migrated cells increased significantly over time, particularly in the presence of DETA-NO. ^•^NO has been reported as an inducer of migration of ER^–^ breast cancer cells,^91^ with its concentrations released from 100 and 300 μM DETA-NO reported to induce the migration of MDA-MB-231 cells.^92^ Although this explains the observed results in the case of DETA-NO-induced cell migration, the presence of HA had different effects depending on its concentration, the flux of ^•^NO existing, and the incubation period. For instance, a significant decrease in cell migration was observed when the chemoattractant contained HA in addition to DETA-NO after 12 h of culture compared to the HA-free medium, while the high levels of ^•^NO produced during the 24 h of culture negated these effects.

Similarly, while only HA at a concentration of 1000 μg/mL was observed to attenuate the enhanced NO-induced cell migration after 12 h of culturing with no significant differences from the untreated cells, this effect was less significant after 24 h of culture (Figure 11B).

The migration of MDA-MB-231 toward a chemoattractant containing HA was dependent on CD44 expression, and these cells were found to exhibit constitutive hyaluronan binding.^8,9^ However, in the presence of ^•^NO, its side reactions with HA seem to have effects on cell migration in two stages: (1) retarding the effects of ^•^NO, released during the first hours of incubation, on cells responsible for their enhanced migration, and this is dependent on the HA concentration; (2) with the continuous release of ^•^NO, and as HA cannot completely abolish its levels, the greater probability of interactions of ^•^NO with the cells exists with its functionality as a chemoattractant causing an enhancement of cell migration. These results indicate that the reaction between HA and ^•^NO is limited by the concentration of each one and by the ^•^NO-release rate. In contrast, when the cells were mixed with DETA-NO in the upper chamber in FBS-free RPMI, its migration rate decreased but was improved in the presence of HA (Figure S17). It should be emphasized here that the degradation of DETA-NO in the former case takes place slowly in the chemoattractant medium containing FBS. At the same time, the rate is faster when mixed with the cells in an FBS-free medium, which may be the reason for the decreased rate of cell migration. However, the enhanced cell migration in the presence of HA confirms the essential roles played by the direct interactions between HA and cell surface receptors and their regulatory functions for cell migration, as reported previously.^93,94^ Moreover, ^•^NO was found to cause the development of a stem cell-like phenotype with upregulation of CD44,^91,95^ so it would be expected to enhance the interactions between the breast cancer cells and HA.

In the case of iNOS-transfected HCC1806 cells, HA in the bottom chamber enhanced the cell migration with 100 μg/mL concentration showing the highest significant efficiency. In comparison, 1000 μg/mL had the lowest efficiency (Figure S12C,D). Moreover, by comparing the effects of HA in the upper and lower chambers, it was found that HA added to the cells in FBS-free RPMI enhanced the migration at a higher rate than in the case of its addition to the chemoattractant medium. This supports the results in the case of MDA-MB-231 cells. In the transwell assay, the cells migrate toward the chemoattractant, which is an FBS-containing medium in the current study. However, considering the reported interactions between HA and various proteins,^96,97^ certain interactions between HA and the medium components, mainly its protein contents, would be expected. With the increase in the concentration of HA, there is a higher probability of these interactions, which decreases the effects of the concentration gradient responsible for the enhancement of cell migration. This can explain these observed results. Although these HA-protein interactions will also be expected in the case of MDA-MB-231 cells, the presence of ^•^NO in the medium seems to alter them. This can be through its nitrosylating effects for HA and proteins.

#### 3.4.5 Wound Healing Assay

**Figure 12:**
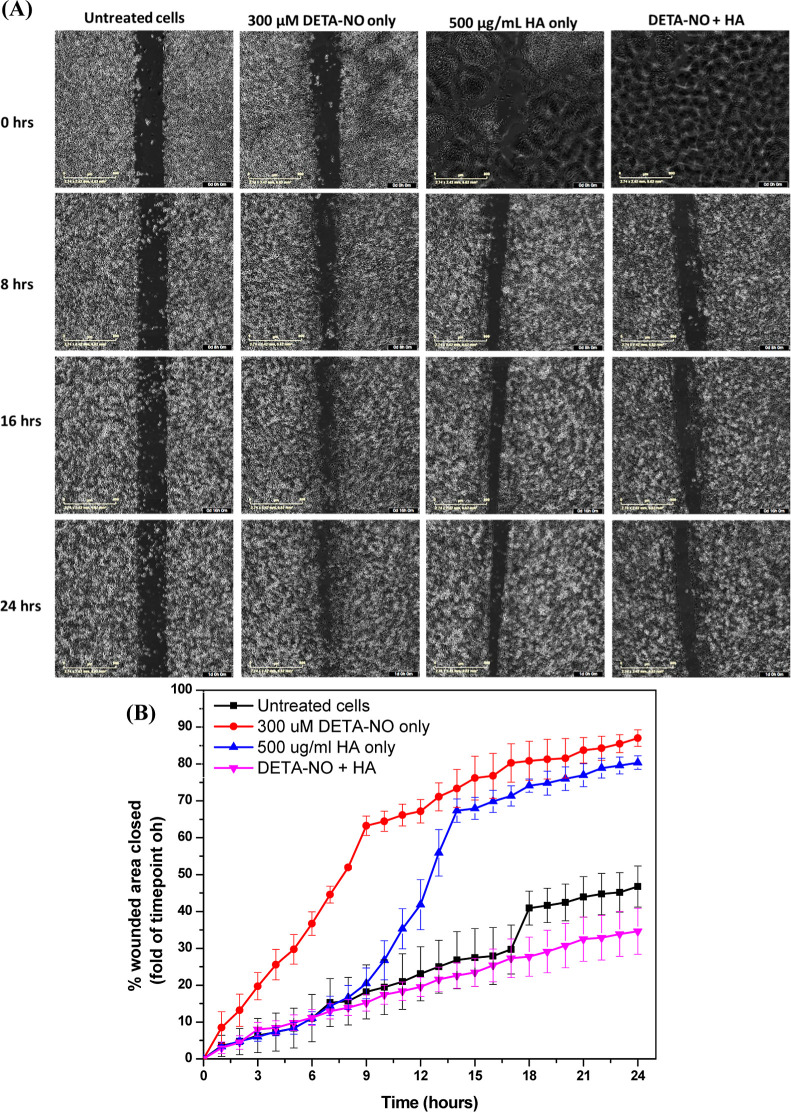
Effects of DETA-NO and 1000 kDa HA on MDA-MB-231 cell migration. (A) Images of scratches at 0, 8, 16, and 24 h in the case of untreated cells or cells treated with 300 μM DETA-NO only, 500 μg/mL HA, or a mixture of 300 μM DETA-NO and 500 μg/mL HA. Scale bar: 800 μm. (B) Quantification of the percentage of the wounded area closed over time concerning the initial wound area (at time 0). The scratch was generated using a 2-well insert. Following the treatment of cells with the different treatments, each well was imaged using an IncuCyte S3 Automated Live-Cell Analysis System at regular intervals of 1 h for 24 h. The change in wound area was quantified using ImageJ software. Data are represented as mean ± SD, *n* = 3.

The cell migration experiments described above depended on cells’ migration within a chemotactic gradient. To determine whether these results are specific to these experimental conditions or the implications of ^•^NO/HA interactions are the same under different experimental designs, the cell migration was further studied via the wound healing assay in the absence of any chemoattractant medium. Here, while DETA-NO enhanced a rapid closure of the gap (scratch), this was inhibited in the presence of HA, relating to interference between the biological effects of ^•^NO and its interactions with HA. Figure 12A shows examples for the images acquired after 0, 8, 16, and 24 h of adding the different treatments to cells, and the quantification of these results is illustrated in Figure 12B. Videos S1, S2, S3, and S4 show the overtime change in the gap due to cell migration following the different treatments.

**Figure 13:**
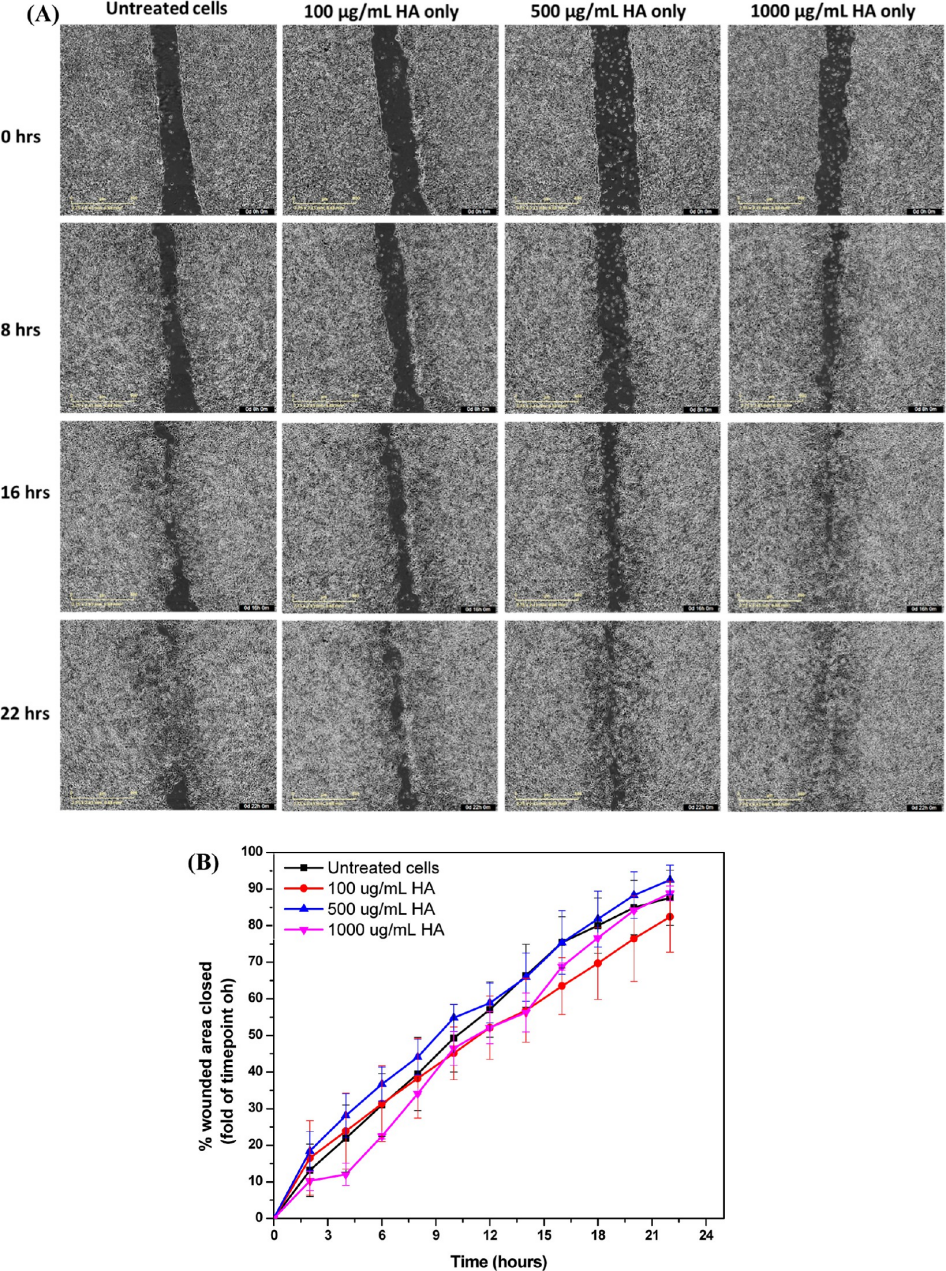
Effects of different concentrations of 1000 kDa HA on iNOS-transfected HCC1806 cell migration. (A) Images of scratches at 0, 8, 16, and 22 h in the case of untreated cells or cells treated with 100, 500, or 1000 μg/mL HA. Scale bar: 800 μm. (B) Quantification of the percentage of the wounded area closed over time concerning the initial wound area (at time 0). The scratch was generated using a 2-well insert. Following the treatment of cells with the different treatments, each well was imaged using the IncuCyte S3 Automated Live-Cell Analysis System at regular intervals of 2 for 22 h. The change in wound area was quantified using ImageJ software. Data are represented as mean ± SD, *n* = 3.

However, although the effects of HA only on cells were not significantly different from those of the untreated and DETA-NO/HA groups during the first 10 h of culturing, the closure rate was enhanced after that, with similar rates of wound closure between the DETA-NO and HA only groups. These results support the previous transwell assay findings. For comparison, the HA products showed different effects on the DETA-NO-induced cell migration, relating to possible different interactions of these modified HA with the cells. At the same time, all of them proved their effectiveness in scavenging ^•^NO and decreasing intracellular ^•^NO levels, particularly after 10 h of incubation (Figure S18). Similar to the results of MDA-MB-231, the treatment of iNOS-transfected HCC1806 with different concentrations of HA did not stop the cell migration, despite their affinity to decrease the levels of intracellular ^•^NO (Figure 13A,B). Videos S5, S6, S7, and S8 show the overtime change in the gap due to cell migration following the different treatments.

This may indicate a separate mechanism by which HA accelerates cell migration and proliferation, independent of its action against the intracellular-produced ^•^NO. Moreover, these results, alongside the previous results of MDA-MB-231 and the transwell migration results, suggest that the interactions between HA existing within the ECM of the tumor tissue and ^•^NO occur principally within the tumor matrix. Accordingly, when designing new drugs and drug-loading carriers targeting the intracellular ^•^NO, the carrier should be able to penetrate the cell membrane, and the drug should have sufficient hydrophobicity.

## 4 Conclusions

In addition to being one of the main components of the ECM in tumor tissues, HA has been employed in the fabrication of various drug delivery vehicles for cancer treatment. While the effects of different ROS on HA have been studied before, the mechanism of its interactions with ^•^NO, as one of the central regulators of tumor cell migration and further tumor metastasis, has not been thoroughly examined. Here, the HA/^•^NO reaction was studied. It was found that the scavenging of ^•^NO by HA was accompanied by NO-induced oxidation reactions involving a series of radical chain reactions, which are finally terminated with cleavage O-glycosidic bonds of the long HA chains with their partial fragmentation. Moreover, ^•^NO’s effects were compared with those of O_2_^•–^, ONOO^•–^, and hyaluronidase targeting different positions on the HA chains. In addition, via the in vitro study, the downstream effects of both ^•^NO and HA on cell attachment and migration were studied alongside their impact on the intracellular ^•^NO. However, a clear understanding of the molecular mechanism by which ^•^NO induces the cell migration alongside the signaling pathways through which nHA induces its effects on breast cancer cells is still required. Moreover, studying the molecular effects of HA binding to the cell surface receptors following its reaction with ^•^NO will help determine their further implications in the migration of breast cancer cells. These studies are ongoing in our lab, and we believe that they will help optimize the composition/formulation of various HA-based drug delivery vehicles for breast cancer.

## Supporting Information Available

Effects of treatment with 300 μM DETA-NO only on MDA-MB-231 (MP4)
MDA-MB-231 cell migration after treatment with 300 μM DETA-NO and 500 μg/mL HA (MP4)
MDA-MB-231 cell migration after treatment with 500 μg/mL HA only (MP4)
MDA-MB-231 cell migration without any treatment (MP4)
iNOS-transfected HCC1806 cell migration without any treatment (MP4)
iNOS-transfected HCC1806 cell migration after treatment with 100 μg/mL HA (MP4)
iNOS-transfected HCC1806 cell migration after treatment with 500 μg/mL HA (MP4)
iNOS-transfected HCC1806 cell migration after treatment with 1000 μg/mL HA (MP4)
Change in voltage readings corresponding to NO measurements; radical scavenging efficiency of HA; ^1^H NMR spectra and FTIR spectra products of the final HA products; viscosity values of HA solutions under different treatments; agarose gel electrophoresis results; metabolic activity results; changes in luminescence intensity results; adhesion study results; intracellular NO measurements; and cell migration results (PDF)

## Abbreviations Used

BSA: bovine serum albumin
CL: chemiluminescence
DAF-FM: diacetate 4-amino-5-methylamino-2′,7′-difluorofluorescein diacetate
DETA-NO: diethylenetriamine NONOate
DMTMM: 4-(4,6-dimethoxy-1,3,5-triazin-2-yl)-4-methylmorpholinium chloride
DPPH: 2,2-diphenyl-1-picrylhydrazyl
ECM: extracellular matrix
HA: hyaluronic acid
lHA: unmodified lyophilized HA
MES: 2-(*N*-morpholino)ethanesulfonic acid
^•^NO: nitric oxide
^•^NO_2_: nitrogen dioxide radical
NO_2_^–^: nitrite
nHA: unmodified non-lyophilized HA
O_2_^•–^: superoxide radical
ONOO^•–^: peroxynitrite
RNS: reactive nitrogen species
ROS: reactive oxygen species
RHAMM: receptor of hyaluronic acid-mediated motility
SIN-1: morpholinylsydnonimine chloride
SNAP: *S*-nitroso-*N*-acetyl-d,l-penicillamine
SNP: sodium nitroprusside
