# Supplementary material for: Interactions between Nitric Oxide and Hyaluronan Implicate the Migration of Breast Cancer Cells
Source: Biomacromolecules. 2022 Aug 3;23(9):3621–47. doi: 10.1021/acs.biomac.2c00545 (PMC9472231; doi:10.1021/acs.biomac.2c00545)
Supplement: Supplementary file 9 — bm2c00545_si_009.pdf [file bm2c00545_si_009.pdf]

Interactions between Nitric Oxide and Hyaluronan Implicate the Migration of Breast Cancer Cells

Amir M. Alsharabasy[1], Sharon Glynn[1,2], Pau Farràs[1,3], Abhay Pandit[1,]*

[1]CÚRAM, SFI Research Centre for Medical Devices, National University of Ireland Galway, Ireland

[2]Discipline of Pathology, Lambe Institute for Translational Research, School of Medicine, National University of Ireland Galway, Ireland

[3]School of Biological and Chemical Sciences, Ryan Institute, National University of Ireland Galway, Ireland

Corresponding Author: **Prof**. **Abhay Pandit**

[1]CÚRAM, SFI Research Centre for Medical Devices,

National University of Ireland Galway,

Ireland

E-mail: Abhay.pandit@nuigalway.ie

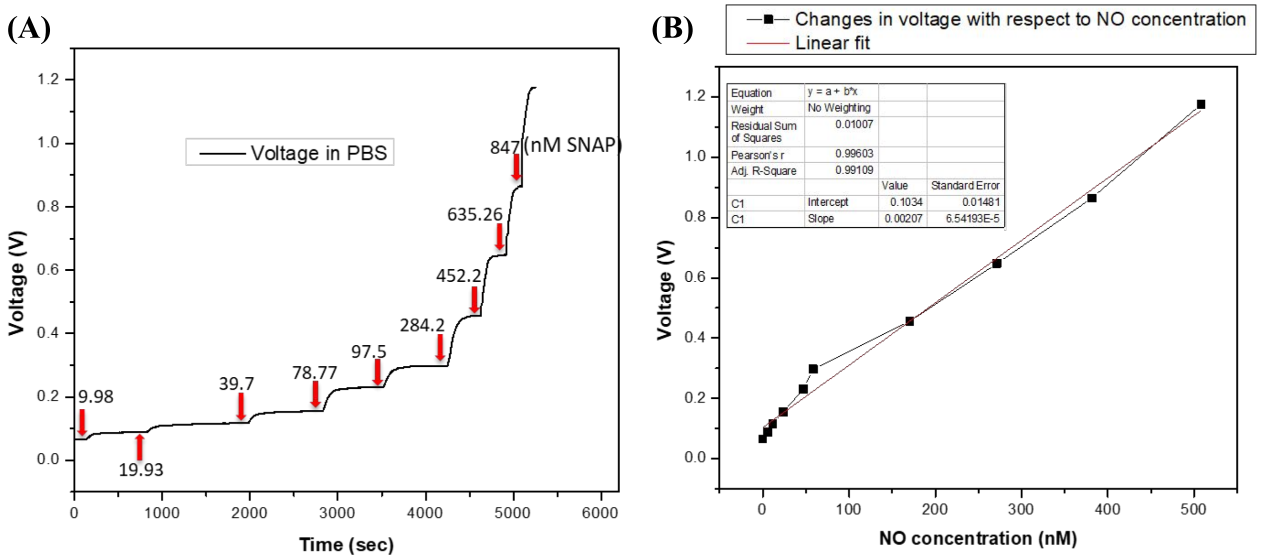

Figure S1: The change in voltage reading following the direct dilution of SNAP in PBS generating different concentrations, and **(b)** the accompanying final standard curve.

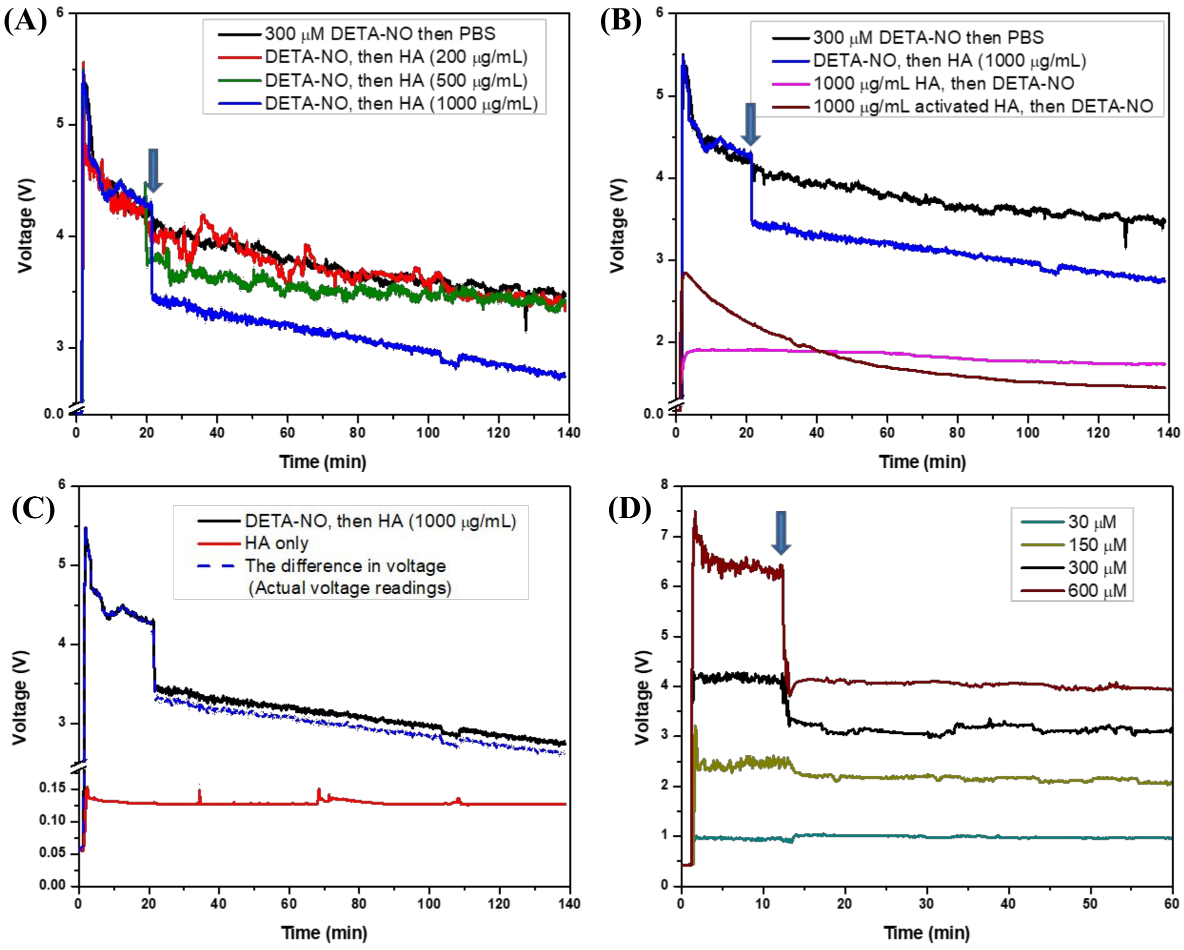

Figure S2: Temporal changes in the voltage signal in PBS: **(A)** The changes in the signal recorded over 140 min after injection of 300 μM DETA-NO to PBS only **(black colour)** or followed by a further addition of different concentrations of 1000 KDa HA, as indicated by an arrow. **(B)** The changes in the signal recorded following the injection of 300 μM DETA-NO to overnight-polarized PBS solution containing 1000 μg/mL of HA or DMTMM-activated HA. **(C)** Comparison of the voltage signal due to injected DETA-NO, followed by 1000 μg/mL HA to the signal due to HA only. **(D)** The changes in the signal following the injection of 30, 150, 300 and 600 μM DETA-NO, followed by the injection of HA for a final concentration of 1000 μg/mL, as indicated by an arrow. The data are represented as the mean voltage reading of three measurements per group.

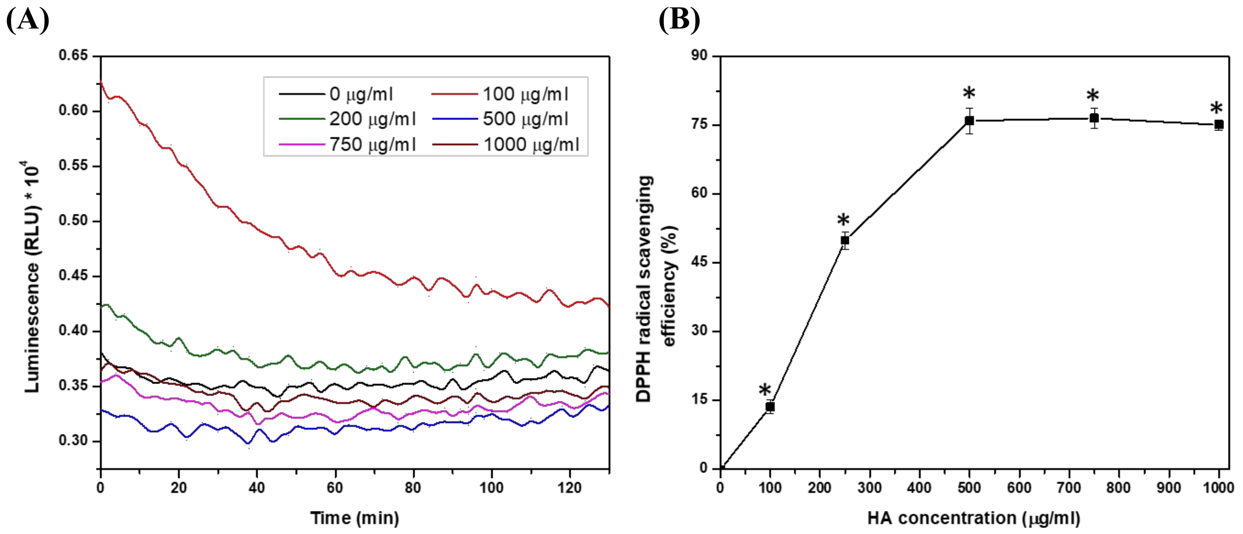

Figure S3: The radical scavenging efficiency of HA: **(A)** the changes in luminescence intensity owing to H_2O_2/luminol in the presence of different concentrations of 1000 KDa HA in phosphate buffer (50 mM, pH 7.4). **(B)** DPPH radical scavenging activities of various concentrations of HA in phosphate buffer. DPPH was mixed with different concentrations of HA in phosphate buffer and incubated at R.T for 30 min before measuring the absorbance at 517 nm. Data are represented as mean ± SD, n = 3. *, $P < 0.05$ versus the HA-free group using a two tailed unpaired student t-test.

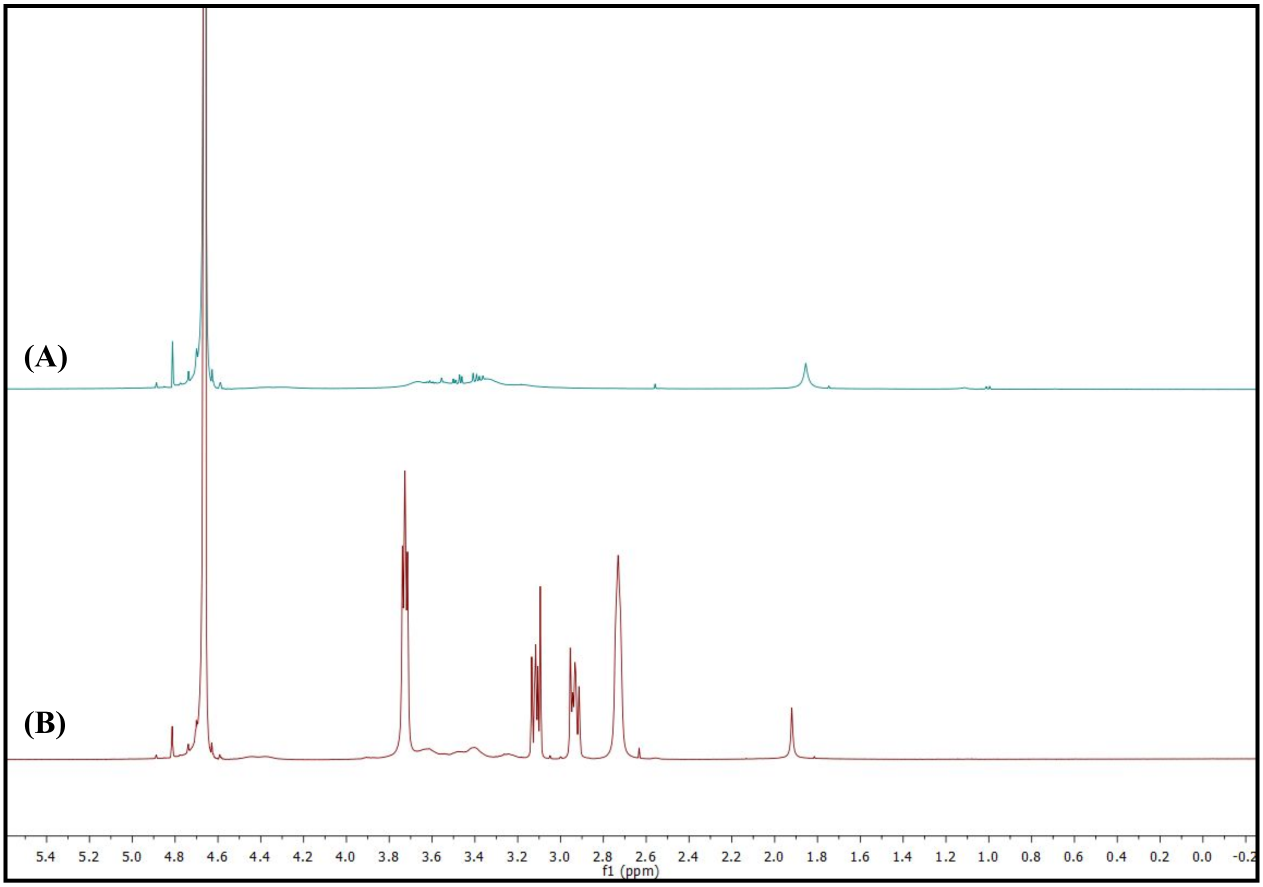

Figure S4: Comparison of the ^{1}H NMR spectra of **(A)** 1000 KDa HA dissolved directly in D_2O and **(B)** HA dissolved in PBS, lyophilised and dissolved in D_2O for the NMR measurements, denoted as (lHA). The NMR spectra were recorded at 400 MHz.

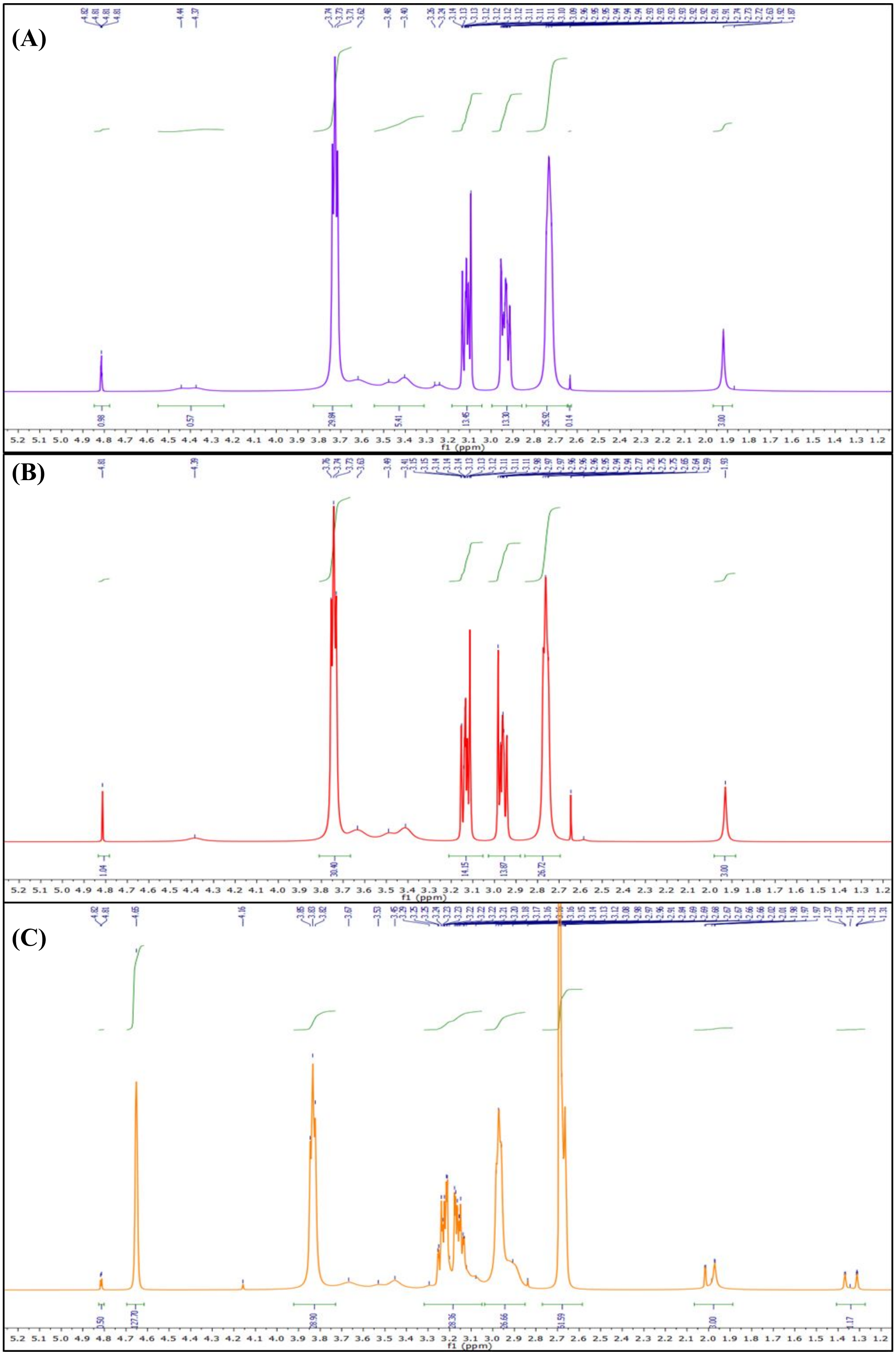
(A)
0.98
0.57
29.84
5.41
13.45
13.30
25.92
0.14
3.00
f1 (ppm)
(B)
1.04
30.40
14.15
13.87
26.72
3.00
f1 (ppm)
(C)
0.50
127.70
28.90
28.36
35.66
31.59
3.00
1.17
f1 (ppm)

Figure S5: Comparison of the ^{1}H NMR spectra of **(A)** 1000 KDa lHA, **(B)** HA/300-SNP, and **(C)** HA/300-SNAP. Following the lyophilisation of the different HA products, they were dissolved in D2O and the NMR spectra were recorded at 400 MHz. A description of each HA product is given in **Table 1** in the main manuscript.

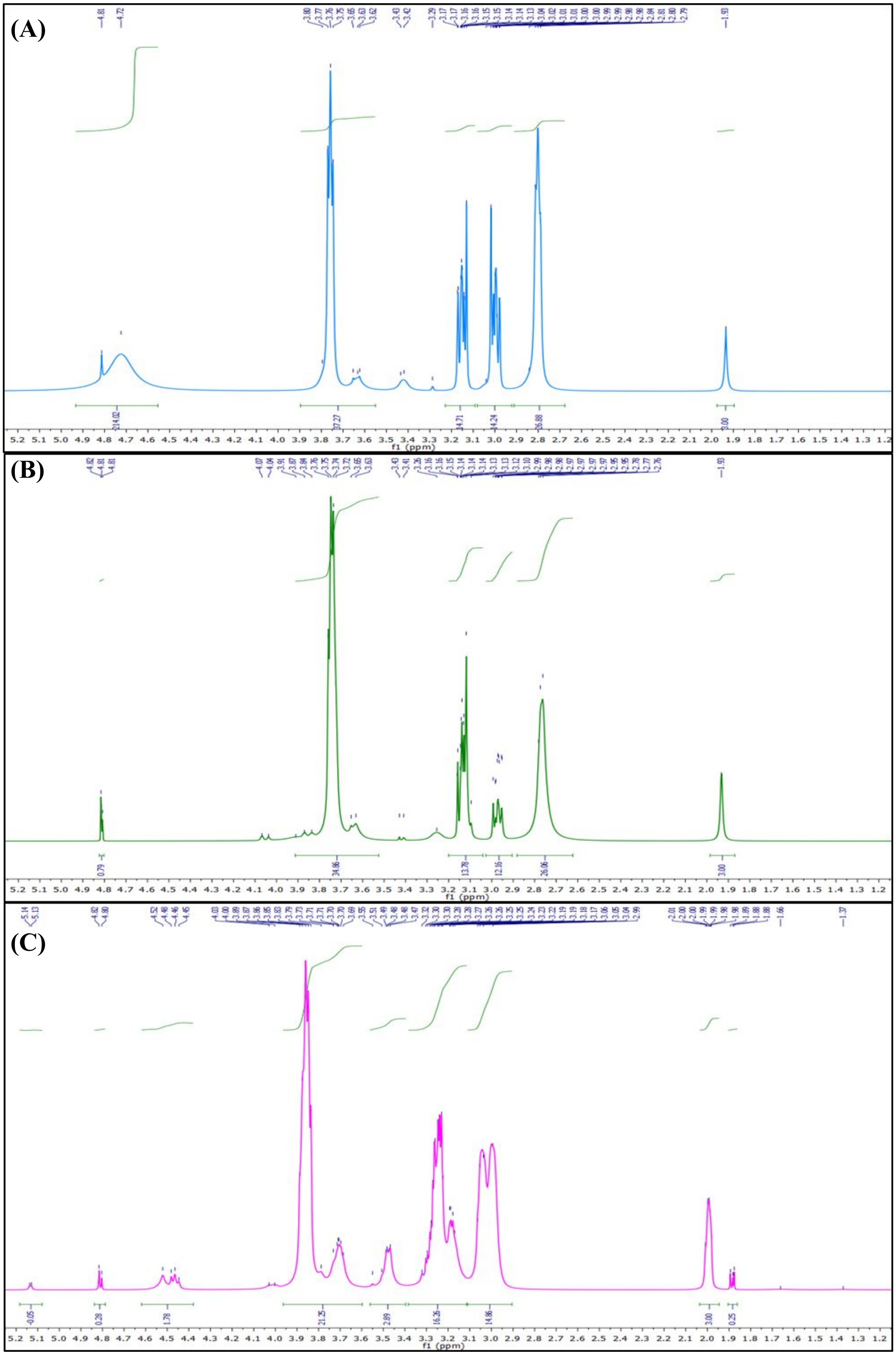
(A)
(B)
(C)
f1 (ppm)

Figure S6: Comparison of the ^{1}H NMR spectra of **(A)** HA/300-SIN-1, **(B)** HA/1000-H_2O_2, and **(C)** HA/HAase. Following the lyophilisation of the different HA products, they were dissolved in D2O and the NMR spectra were recorded at 400 MHz. A description of each HA product is given in **Table 1** in the main manuscript.

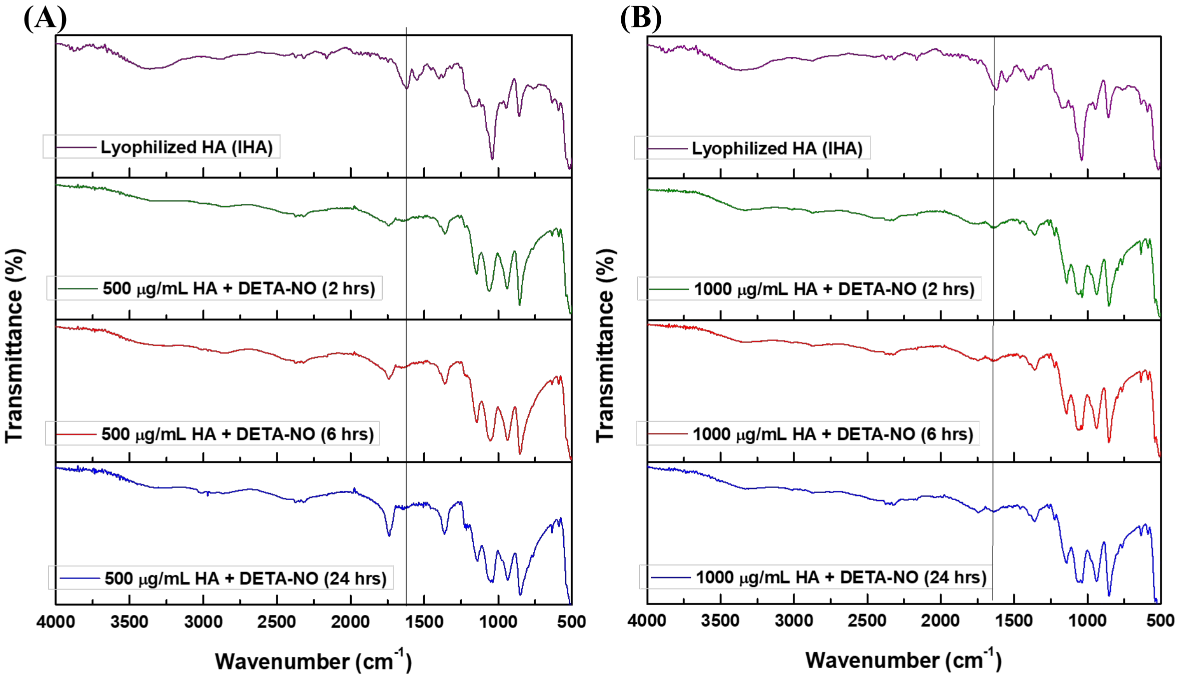

Figure S7: FTIR spectrum of untreated 1000 KDa HA, and the lyophilized products from 500 **(A)** and 1000 µg/mL HA **(B)** dissolved in PBS, and treated with 300 µM DETA-NO for 2, 6 and 24 hours at 37°C. A description of each HA product is given in **Table 1** in the main manuscript.

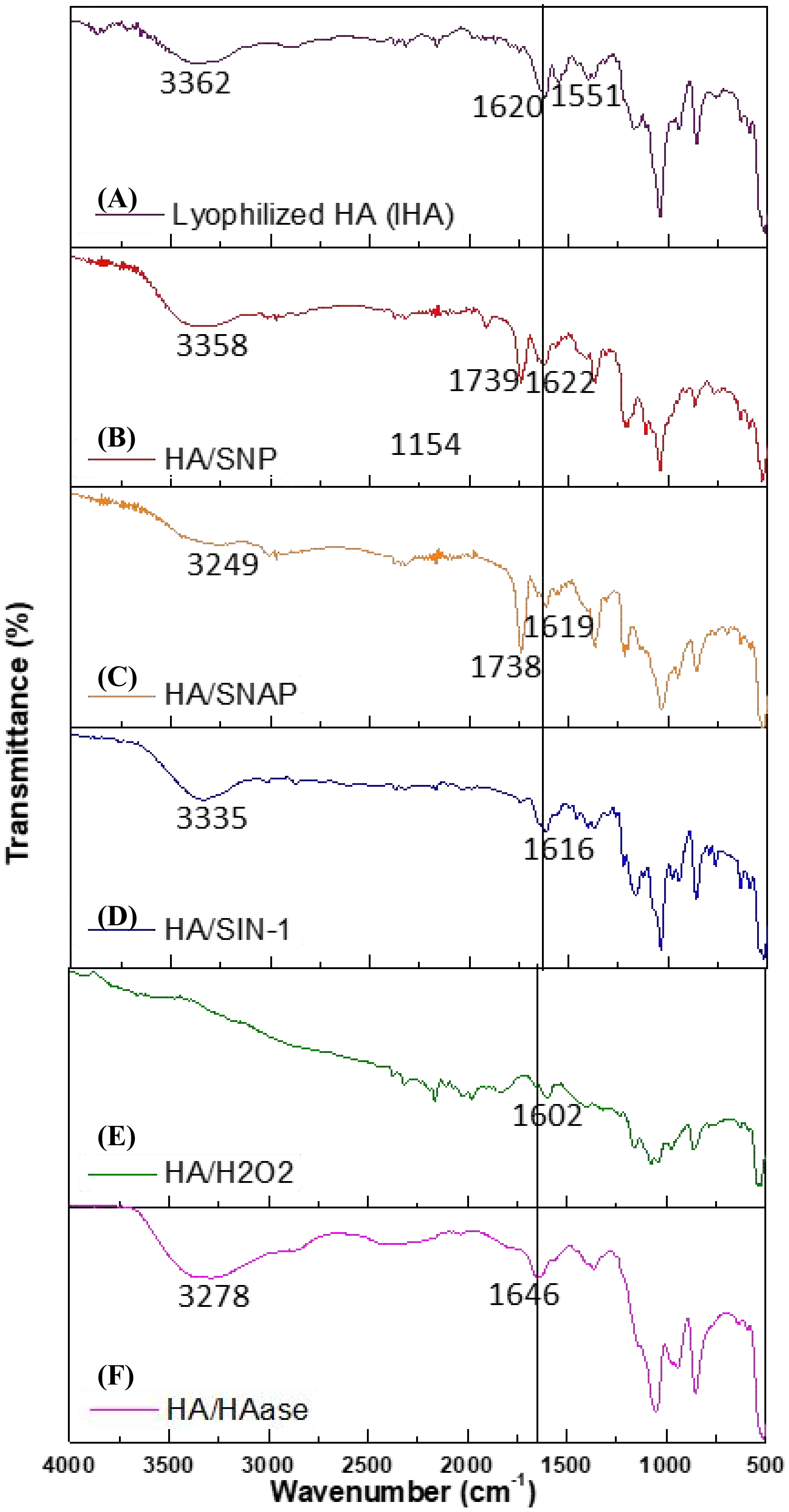

3362
1620
1551
(A) Lyophilized HA (lHA)
3358
1739
1622
1154
(B) HA/SNP
3249
1619
1738
(C) HA/SNAP
3335
1616
(D) HA/SIN-1
1602
(E) HA/H2O2
3278
1646
(F) HA/HAase
Transmittance (%)
4000 3500 3000 2500 2000 1500 1000 500
Wavenumber (cm-1)

Figure S8: FTIR spectrum of lyophilized untreated 1000 KDa HA, HA/SNP, HA/SNAP, HA/SIN-1, HA/H_2O_2, and HA/HAase and HA treated with 300 μM SNP, SNAP, and SIN-1 as well as 1000 μM H_2O_2 and 100 U/mL hyaluronidase (HAase) in PBS for 24 hours at 37°C, followed by lyophilisation and testing. A description of each HA product is given in **Table 1** in the main manuscript.

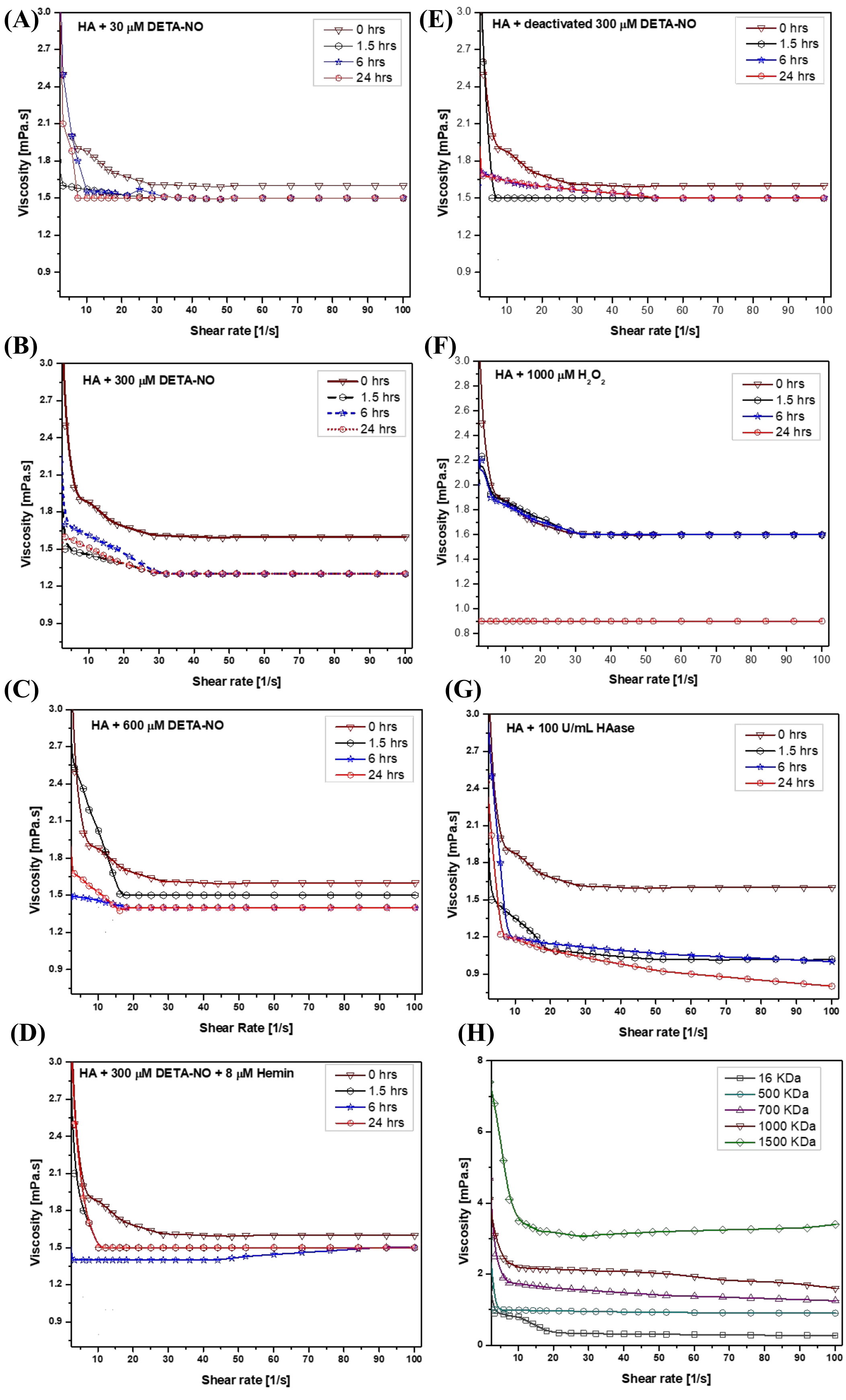

(A)
HA + 30 μM DETA-NO
0 hrs
1.5 hrs
6 hrs
24 hrs
Viscosity [mPa.s]
Shear rate [1/s]
(B)
HA + 300 μM DETA-NO
0 hrs
1.5 hrs
6 hrs
24 hrs
Viscosity [mPa.s]
Shear rate [1/s]
(C)
HA + 600 μM DETA-NO
0 hrs
1.5 hrs
6 hrs
24 hrs
Viscosity [mPa.s]
Shear Rate [1/s]
(D)
HA + 300 μM DETA-NO + 8 μM Hemin
0 hrs
1.5 hrs
6 hrs
24 hrs
Viscosity [mPa.s]
Shear rate [1/s]
(E)
HA + deactivated 300 μM DETA-NO
0 hrs
1.5 hrs
6 hrs
24 hrs
Viscosity [mPa.s]
Shear rate [1/s]
(F)
HA + 1000 μM H2O2
0 hrs
1.5 hrs
6 hrs
24 hrs
Viscosity [mPa.s]
Shear rate [1/s]
(G)
HA + 100 U/mL HAase
0 hrs
1.5 hrs
6 hrs
24 hrs
Viscosity [mPa.s]
Shear rate [1/s]
(H)
16 KDa
500 KDa
700 KDa
1000 KDa
1500 KDa
Viscosity [mPa.s]
Shear rate [1/s]

Figure S9: Viscosity values of HA solutions under different treatments. **(A-G)** The measured change in viscosity over time of 1000 μg/mL of 1000 KDa HA with differing the share rate from 0.001 to 100 1/s, following its treatment with 30 μM DETA-NO (HA/30-DETA-NO) **(A)**, 300 μM DETA-NO (HA/300-DETA-NO) **(B)**, 600 μM DETA-NO (HA/600-DETA-NO) **(C)**, 300 μM DETA-NO and 8 μM hemin (HA/300-DETA-NO/8-hemin) **(D)**, deactivated 300 μM DETA-NO (HA/Deactivated-300-DETA-NO) **(E)**, 1000 μM H_2O_2 (HA/H_2O_2) **(F)** and 100 U/mL hyaluronidase (HA/HAase) **(G)**. At time 0. the initial viscosity of HA in PBS was measured **(wine colour)**, followed by mixing with the different additives and incubation at 37oC for 24 hours, with measurement of the viscosity of each solution after 1.5 **(black colour)**, 6 **(blue colour)** and 24 hours **(red colour)**. **(H)** Comparison of the viscosity of HA with the MW 16 **(grey colour)**, 500 **(cyan colour)**, 700 **(purple colour)**, 1000 **(wine colour)** and 1500 KDa **(olive colour)** dissolved in PBS for a final concentration of 1000 μg/mL.

(A)

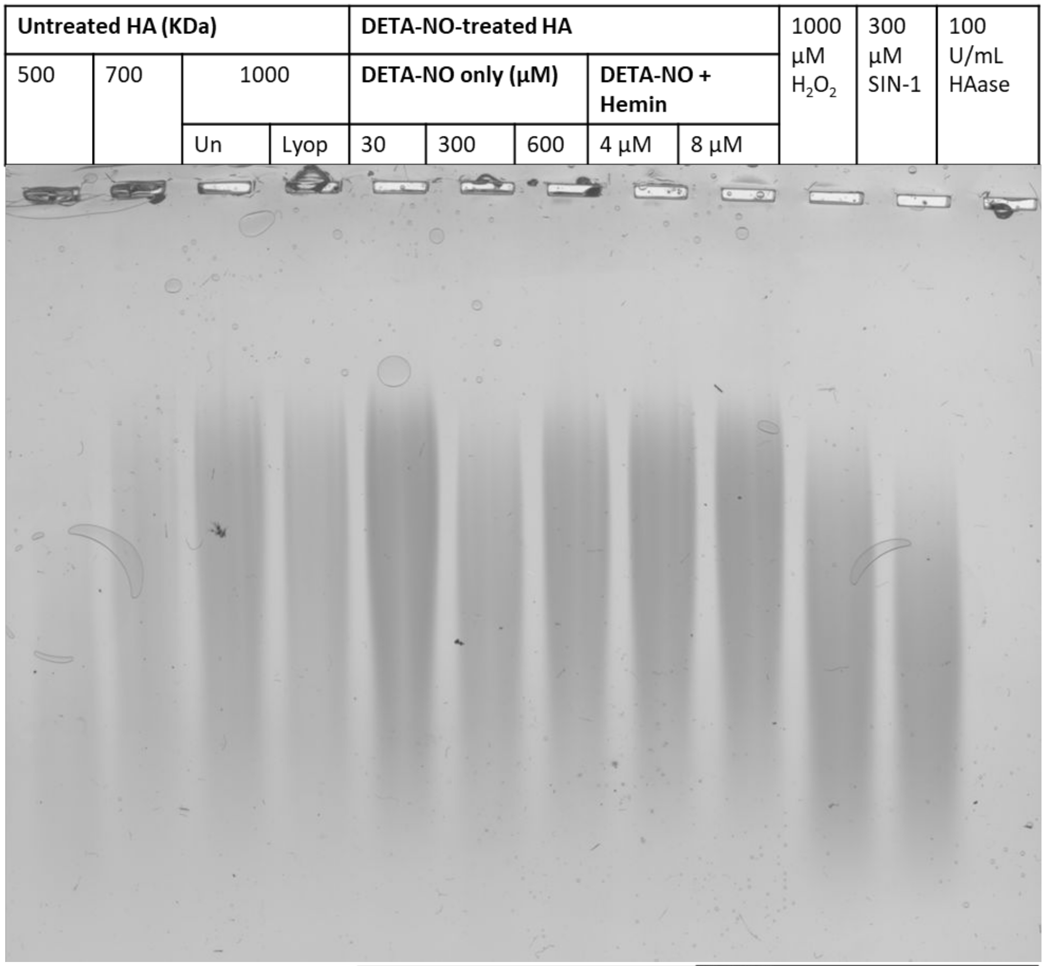
Untreated HA (KDa)
DETA-NO-treated HA
1000 μM H2O2
300 μM SIN-1
100 U/mL HAase
500
700
1000
DETA-NO only (μM)
DETA-NO + Hemin
Un
Lyop
30
300
600
4 μM
8 μM

(B)

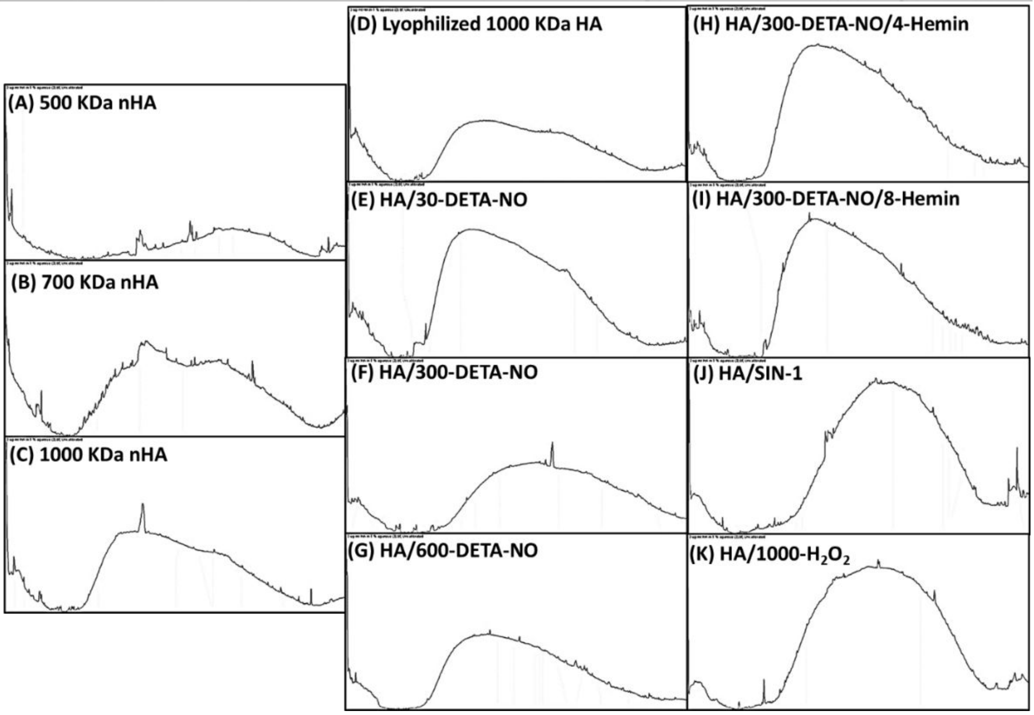
(A) 500 KDa nHA
(B) 700 KDa nHA
(C) 1000 KDa nHA
(D) Lyophilized 1000 KDa HA
(E) HA/30-DETA-NO
(F) HA/300-DETA-NO
(G) HA/600-DETA-NO
(H) HA/300-DETA-NO/4-Hemin
(I) HA/300-DETA-NO/8-Hemin
(J) HA/SIN-1
(K) HA/1000-H2O2

Figure S10: (A) Agarose gel electrophoresis of unmodified HA with different MW, lyophilized untreated 1000 KDa HA and HA treated with 30, 300 and 600 μM DETA-NO, 300 μM SIN-1, 1000 μM H_2O_2 and 100 U/mL hyaluronidase. Additionally, HA batches treated with 300 μM and 4 and 8 μM hemin are shown. The assignment of each well is shown at the top of the Figure. **(B)** The OD profiles of separated HA through the gel (Y-axis) versus their relative mobility (X-axis), extracted from the electrophoresis blots using ImageJ software. The profile corresponding to each separate band was measured from the lower edge of each well in **A** (shown at the far left of each subgraph) to a point at the edge fixed to all wells (shown at the far right). Following the treatment, each sample was incubated at 37 ^{o}C for 24 hours, freeze-dried, dissolved in PBS and the migration of the products was evaluated by agarose gel electrophoresis and stained with Stains-all stain. A description of each HA product is given in **Table 1** in the main manuscript.

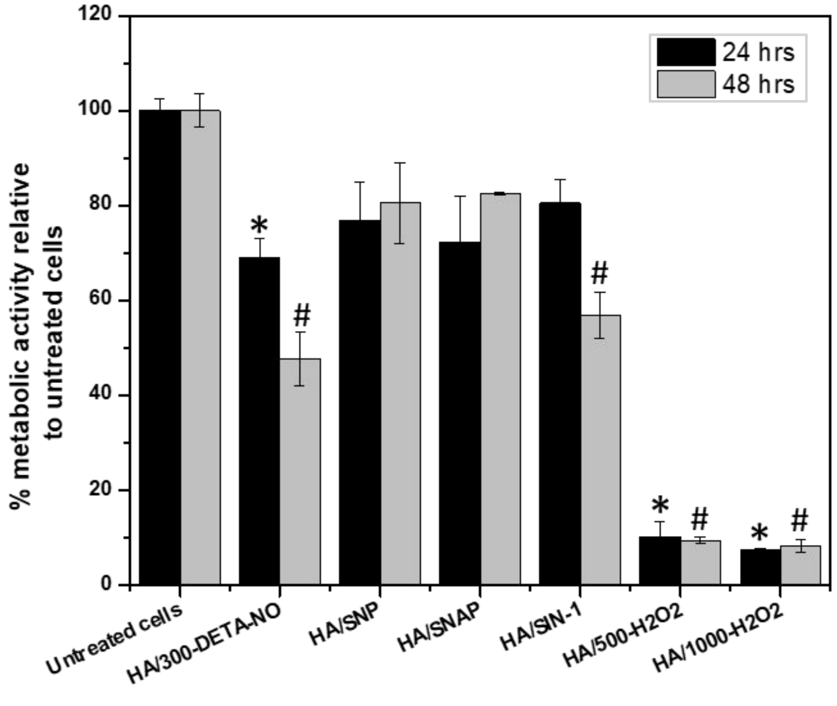

Figure S11: Metabolic activity of MDA-MB-231 after 24 **(black colour)** and 48 hours **(grey colour)** of culture with 300 μM DETA-NO (HA/300-DETA-NO), 300 μM SNP (HA/SNP), 300

μM SNAP (HA/SNAP), 300 μM SIN-1 (HA/SIN-1), 500 μM H_2O_2 (HA/500-H_2O_2) and 1000 μM H_2O_2 (HA/1000-H_2O_2). Each HA product was diluted in FBS-free RPMI to a final concentration of 500 μg/mL, added to the cells, which were cultured further at 37°C in 5% CO_2 and the metabolic activity was measured using the alamarBlue™ assay. Data are represented as mean ± SD, n = 3. $^{*,\#}$, $P < 0.05$ versus the cells treated with lHA only for 24 and 48 hours, respectively using a two tailed unpaired student t-test. A description of each HA product is given in **Table 1** in the main manuscript.

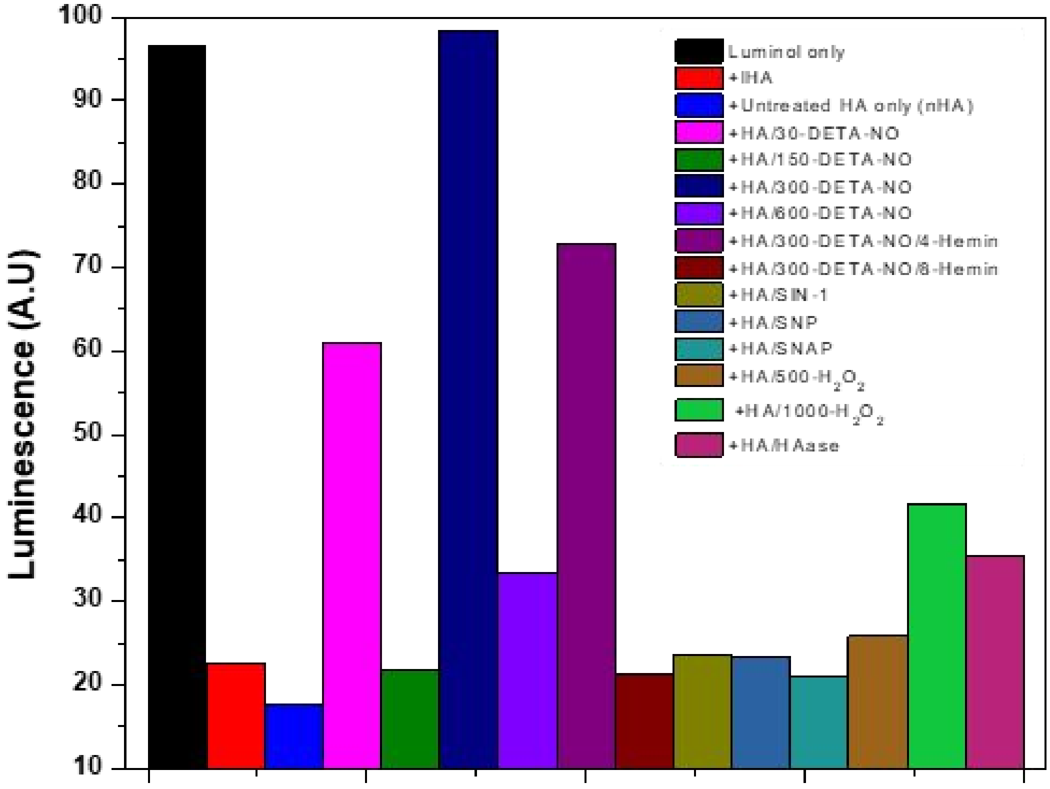

Figure S12: The changes in luminescence intensity owing to 1 mM luminol in the presence of different HA products (500 μg/mL) in phosphate buffer (50 mM, pH 7.4) at 37 °C. Data are represented as the mean readings of three samples taken after mixing for 50 min.

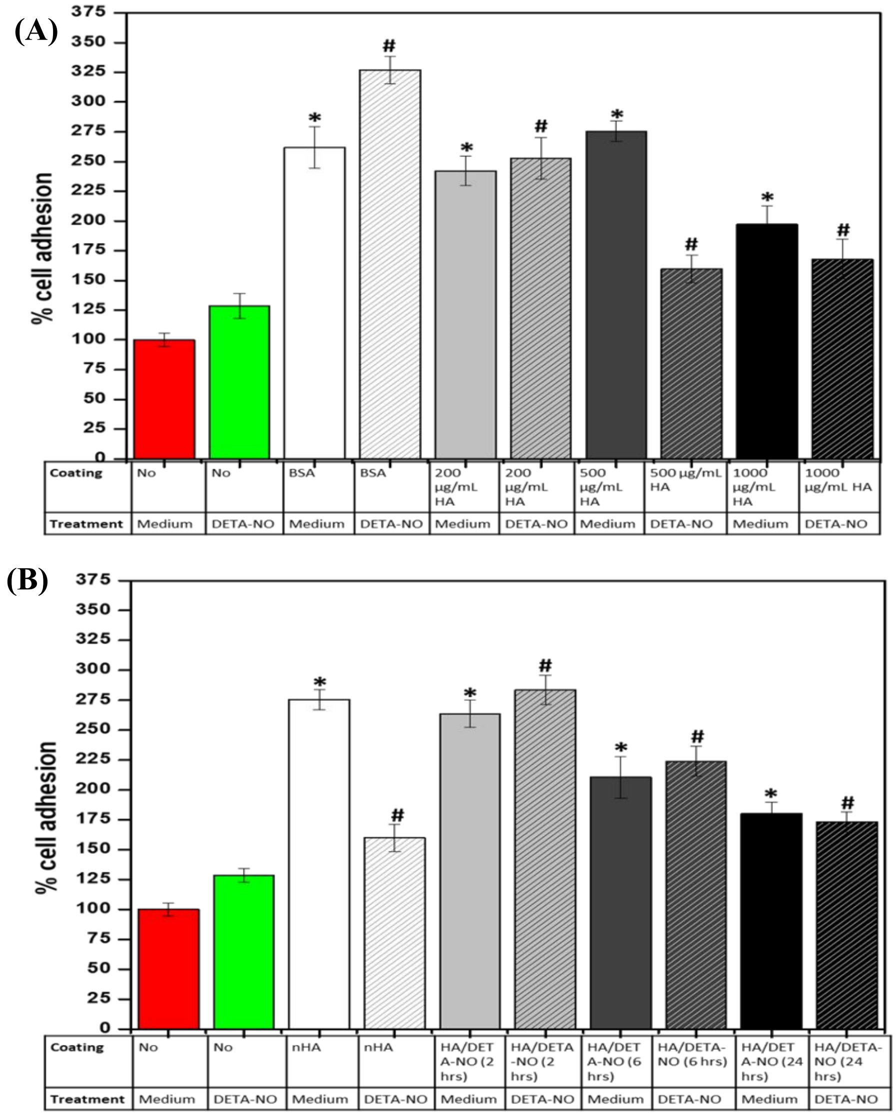

Figure S13: Adhesion of MDA-MB-231 cells to **(A)** pre-coated multi-well plate with BSA or different concentrations of 1000 KDa HA. **(B)** pre-coated plates with nHA or HA/300-DETA-NO product following treatment of HA with 300 μM DETA-NO for 2, 6 and 24 hours. Following coating, the cells were seeded to each well in the presence or absence of •NO added in the form of DETA-NO (300 μM) and cultured for 24 hrs before counting of the number of attached cells. Bars represent mean values of the percentage of cell adhered adherent cells ± SD, n = 3. *, #, $P < 0.05$ versus the untreated and DETA-NO-treated cells without coating, respectively using a two tailed unpaired student t-test.

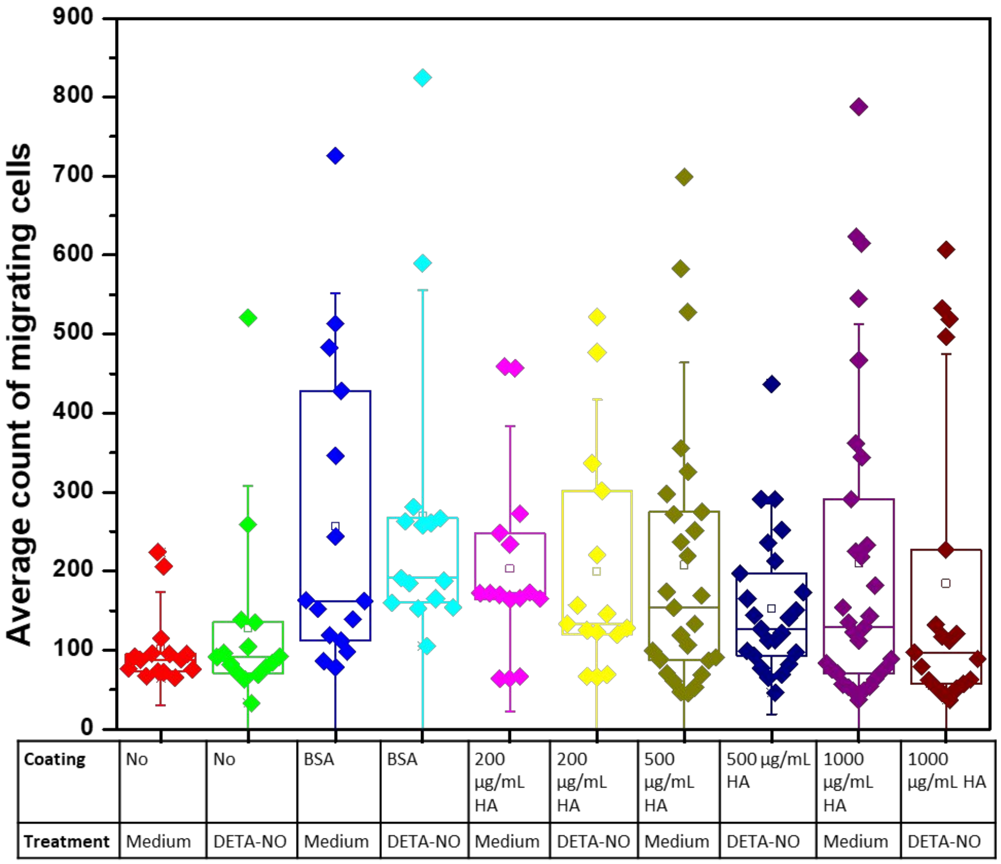

Figure S14: Box-whisker blot showing the distribution of the number of counted MDA-MB-231 cells adhered to BSA and 1000 KDa HA-pre-coated multi-well plates. The wells were either not coated, or pre-coated with BSA or different concentrations of HA, then cells were seeded in the presence or absence of •NO, added in the form of DETA-NO (300 µM), and cultured for 24 hrs before counting of the number of attached cells. The whiskers represent the SD values, n = 3.

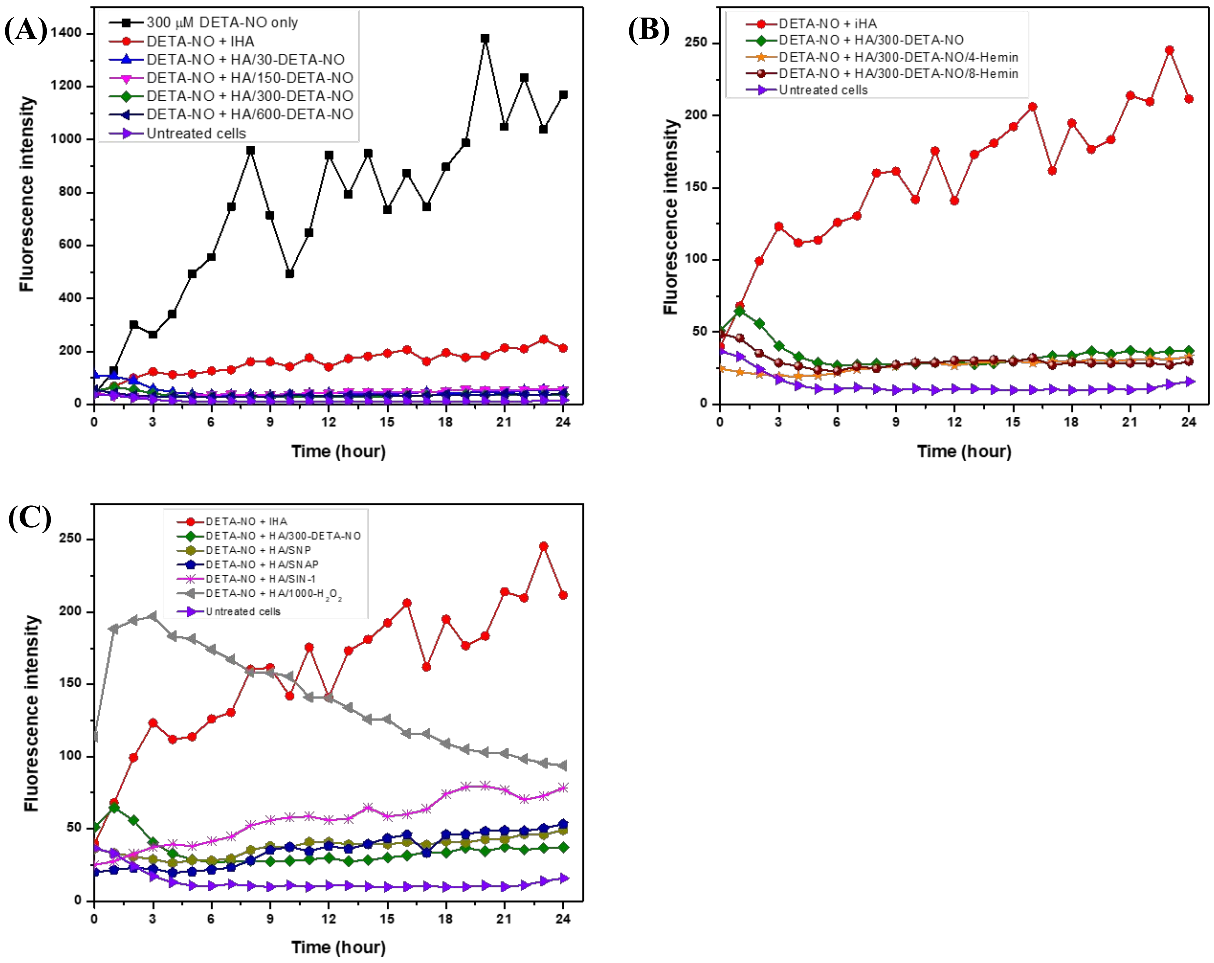

Figure S15: Kinetics of the changes in intracellular •NO levels revealed by the •NO -specific indicator DAF-FM-DA and its fluorescence in MDA-MB-231 cells in response to 300 µM DETA-NO and **(A)** 1000 KDa Lyophilized HA (lHA), HA/30-DETA-NO, HA/150-DETA-NO, HA/300-DETA-NO, HA/600-DETA-NO, **(B)** HA/300-DETA-NO, HA/300-DETA-NO/4-hemin, and HA/300-DETA-NO/8-hemin, and **(C)** HA/300-DETA-NO, HA/SNP, HA/SNAP, HA/SIN-1**,** HA/1000-H_2O_2**.** The cells were treated with DAF-FM-DA for 1 hour, then photographed after the addition of the different treatments using the real-time Incucyte® imaging system (phase contrast and green fluorescence signals). Data are represented as the mean of readings of three samples per group. A description of each HA product is given in **Table 1** in the main manuscript.

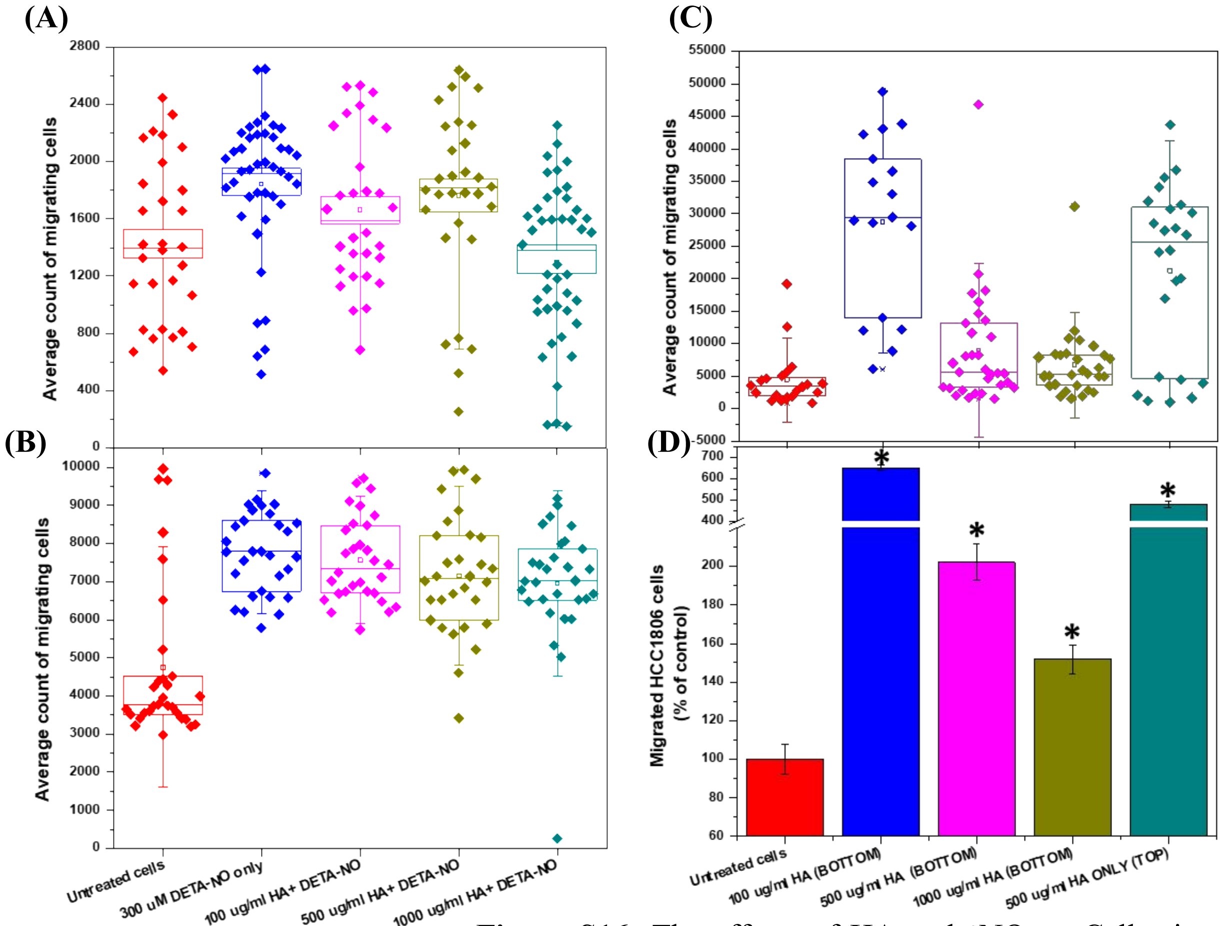

Figure S16: The effects of HA and •NO on Cell migration. **(A,B)** Detailed Box-whisker blots showing the distribution of the number of counted MDA-MB-231 cells migrated through the transwell membranes for 12 **(A)** and 24 hours **(B)**. The cells migrated towards the chemoattractant composed of 300 μM DETA-NO in FBS-containing RPMI in the presence or absence of 100, 500, and 1000 μg/mL 1000 KDa HA, followed by counting the number of migrated cells. **(C)** Box-whisker blot showing the distribution of the number of counted cells migrated through the transwell membranes towards the chemoattractant composed of 100, 500, or 1000 μg/mL 1000 KDa HA in FBS-containing RPMI or the migration of cells mixed with 500 μg/mL HA in FBS-free RPMI towards FBS-containing RPMI following culturing for 24 hours. The whiskers represent the SD values. **(D)** % of migrated cells, normalized to the count in

the control group (untreated cells migrated towards the medium only), and the data are represented as mean ± SD, n = 3. *, $P < 0.05$ versus the untreated cells (Negative control) using a two tailed unpaired student t-test.

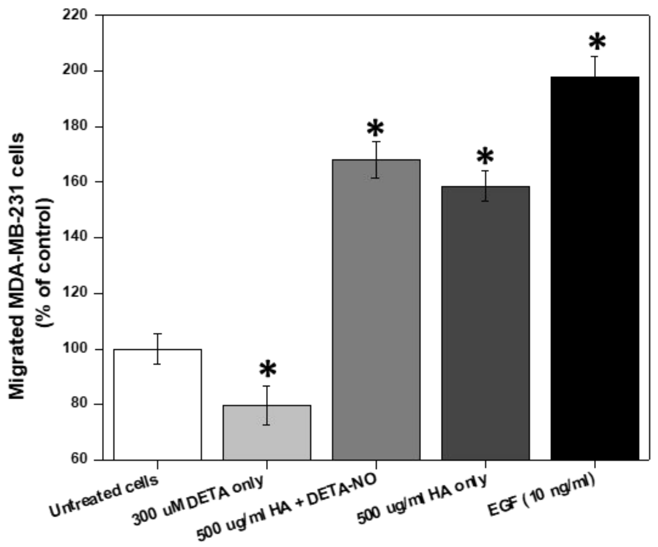

Figure S17: The % of migrated cells, normalized to the count in the control group (untreated cells migrated towards the medium only), following their culture with 300 µM DETA-NO, 500 µg/mL 1000 KDa HA, or a mixture of both in the upper chamber of the transwell. The cells were cultured for 24 hours for migration towards the FBS-containing RPMI in the lower chamber, and the number of migrated cells was counted. EGF (10 ng/mL) was employed as the positive control. Data are represented as mean ± SD, n = 3. *, $P < 0.05$ versus the untreated cells (Negative control) using a two tailed unpaired student t-test.

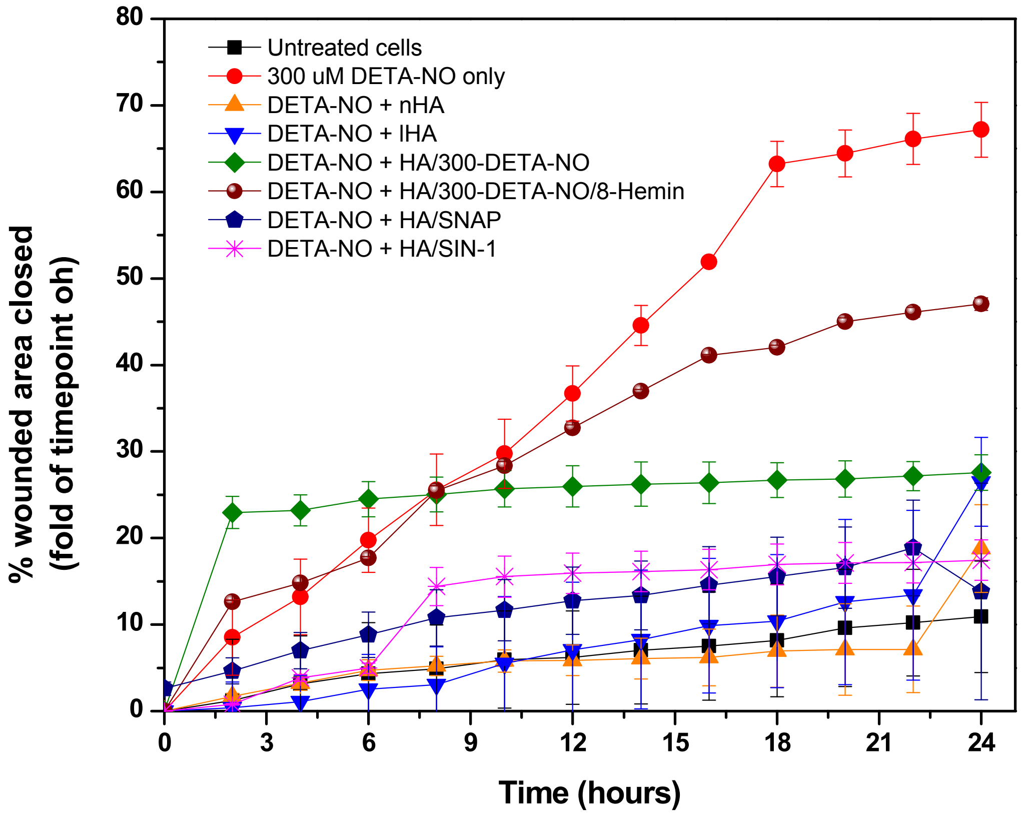

Figure S18: The effects of DETA-NO and different HA products on MDA-MB-231 cell migration reported by quantifying the percentage of the wounded area closed over time with respect to the initial wound area (at time 0). The scratch was generated using a 2-well insert, and, following the treatment of cells with the 300 μM DETA-NO and 500 μg/mL of one of the HA products, each well was imaged using the IncuCyte® S3 Automated Live-Cell Analysis System at regular intervals of 1 hour for a total period of 24 hours. The change in wound area was quantified using ImageJ software. Data are represented as mean ± SD, n = 3. A description of each HA product is given in **Table 1** in the main manuscript.

Video S1 to be inserted here

Video S1: The effects of 300 μM DETA-NO only on MDA-MB-231 cell migration, measured by scratch assay. The images shown are from one representative dataset out of tested three samples per the group. Following cell treatment, each well was imaged using the IncuCyte® S3 Automated Live-Cell Analysis System at regular intervals of 1 hour for 24 hours. Scale bar, 800 μm.

Video S2 to be inserted here

Video S2: The effects of 300 μM DETA-NO and 1000 KDa HA on MDA-MB-231 cell migration, measured by scratch assay. The images shown are from one representative dataset out of tested three samples per the group. Following cell treatment, each well was imaged using the IncuCyte® S3 Automated Live-Cell Analysis System at regular intervals of 1 hour for 24 hours. Scale bar, 800 μm.

Video S3 to be inserted here

Video S3: The effects of 500 μg/mL of 1000 KDa HA only on MDA-MB-231 cell migration, measured by scratch assay. The images shown are from one representative dataset out of tested three samples per the group. Following cell treatment, each well was imaged using the IncuCyte® S3 Automated Live-Cell Analysis System at regular intervals of 1 hour for 24 hours. Scale bar, 800 μm.

Video S4 to be inserted here

Video S4: The migration of MDA-MB-231 cells, measured by scratch assay without any treatment. The images shown are from one representative dataset out of tested three samples per

the group. Each well was imaged using the IncuCyte® S3 Automated Live-Cell Analysis System at regular intervals of 1 hour for 24 hours. Scale bar, 800 μm.

Video S5 to be inserted here

Video S5: The migration of iNOS-transfected cells, measured by scratch assay without any treatment. The images shown are from one representative dataset out of tested three samples per the group. Each well was imaged using the IncuCyte® S3 Automated Live-Cell Analysis System at regular intervals of 1 hour for 24 hours. Scale bar, 800 μm.

Video S6 to be inserted here

Video S6: The effects of 100 μg/mL of 1000 KDa HA only on HCC1806 cell migration, measured by scratch assay. The images shown are from one representative dataset out of tested three samples per the group. Following cell treatment, each well was imaged using the IncuCyte® S3 Automated Live-Cell Analysis System at regular intervals of 1 hour for 24 hours. Scale bar, 800 μm.

Video S7 to be inserted here

Video S7: The effects of 500 μg/mL of 1000 KDa HA on HCC1806 cell migration, measured by scratch assay. The images shown are from one representative dataset out of tested three samples per the group. Following cell treatment, each well was imaged using the IncuCyte® S3 Automated Live-Cell Analysis System at regular intervals of 1 hour for 24 hours. Scale bar, 800 μm.

Video S8 to be inserted here

Video S8: The effects of 1000 μg/mL of 1000 KDa HA only on HCC1806 cell migration, measured by scratch assay. The images shown are from one representative dataset out of tested three samples per the group. Following cell treatment, each well was imaged using the IncuCyte® S3 Automated Live-Cell Analysis System at regular intervals of 1 hour for 24 hours. Scale bar, 800 μm.

Supplementary Table 1: Average peak wave numbers of 1000 KDa lHA, HA/30-DETA-NO, HA/150-DETA-NO, HA/300-DETA-NO, HA/600-DETA-NO, HA/300-DETA-NO/8-Hemin and Native HA (nHA) and their approximate assignment. The main bands of untreated lyophilized HA (lHA) are denoted from **a** to **l**, and the new bands observed in the HA products are denoted from **m** to **t**. A description of each HA product is given in **Table 1** in the main manuscript**.**

**No.**	**Band (cm^{-1})**	**Approximate assignments**	**HA products**						**Native HA**
			**HA/30-DETA-NO**	**HA/150-DETA-NO**	**HA/300-DETA-NO**	**HA/600-DETA-NO**	**HA/300-DETA-NO/4-Hemin**	**HA/300-DETA-NO/8-Hemin**	
**a**	3362	OH and NH stretching	3293	Broad	Broad	Broad	3389	3326 (broad)	3301
**b**	2874	CH stretching	2881	2840	2871	2877	2876	2868	2886
**c**	1620	Amide I (C=O stretching)	1624	1639	1641	1641	1618	1620	1610
**d**	1551	Amide II (N-H bending and C-N stretching)	1547 (weak)	1547	1543	1542	1561	1555	1553 (weak)
**e**	1454		1456 (weak)	1466	1457	X	1453	1457	X

**f**	1401	C-O stretching	1401 (weak)	1408	1408	1407	1407	1404	1406
**g**	1373		1374 (weak)	X	1374	1393	1375	136	1376
**h**	1316	Amide III	1615	1323	X	X	1311	1312	1319
**i**	1154	Carbohydrate peaks (C-O, O-H and C-O-H deformation 1154: C-O-C (O-bridge) 1039.5: C-OH group 945.6: Asymmetrical out-of-phase ring vibration	1179 (strong)	1179	1145	1179	1178	1165	1149
**j**	1039		1038	1038	1042	1044	1040	1037	1039
**k**	977		980 (Stronger)	971 (very weak)	X	X	X	X	X
**l**	945		X	X	938	X	948	947	947
	New bands in the HA products								
**m**	1514	may be NO_2-stretching	1541	1528 (strong)	1525	1522	X	X	X

**n**	1787		X	1787	1787	1787	X	X	X
**o**	1740	C=O stretching of new amide bond	1746	1746	1745	1745	X	1747 (very weak)	X
**p**	1224	C-N stretching	1224	X	1224	X	1219	X	1238 (very weak)
**q**	1261		1261	X	Yes	X	1253		X
**r**	1062	C-O (exocyclic)	1054	X	Yes		X	X	X
**s**	2166		2157		2169 (very weak)	Yes	X	X	X
**t**	1684			1687		Yes	X	X	X

Supplementary Table 2: Average peak wave numbers of the 1000 KDa lHA, and HA treated at the concentration 500 and 1000 μg/mL with 300 μM DETA-NO for 2, 6 and 24 hours and their approximate assignment. The main bands of untreated lyophilized HA (lHA) are denoted from **a** to **l**, and the new bands observed in the HA products are denoted from **m** to **v**.

**No.**	**Band (cm^{-1})**	**Approximate assignments**	**HA/DETA-NO products**

			500 µg/mL HA			1000 µg/mL HA		
			2 hrs	6 hrs	24 hrs	2 hrs	6 hrs	24 hrs
**a**	3362	OH and NH stretching	Very broad	Very broad	Very broad	3334	3169 (Very broad)	Broad
**b**	2874	CH stretching	2868	2865	2970	2870	2868	2871
**c**	1620	Amide I (C=O stretching)	1639 (very weak)	1639.5924	X	1639	1639	1641
**d**	1550	Amide II (N-H bending and C-N stretching)	1547.65 (very weak)	X	X	1544 (very weak)	X	1543
**e**	1454		X	X	1458 (very weak)	1459	1455	1457
**f**	1401	C-O stretching	1396	1395	1395	1402	1398	1408
**g**	1373		1362	1363	1364	1361	1362	1374
**h**	1316	Amide III	X	X	X	X	X	X
**i**	1150	Carbohydrate peaks (C-O, O-	1146	1145	1141	1140	1142	1145

**j**	1039	H and C-O-H deformation	X	X	1038	1037	1038	1042
**k**	977		X	X	976 (very weak/shoulder)	976	X	X
**l**	945		939	937	935	937	938	938
**m**	1514	may be NO_2-stretching	1524	1524	1525	1525	1524	1525
**n**	1787		1787	1787	1787	1787	1787	1787
**o**	1740	C=O stretching of new amide bond	1745	1741	1738	X	1750	1745
**p**	1224	C-N stretching	1225	1227	1227	1224	1223	1224
**q**	1261		X	X	X	1262 (very weak)	X	X
**r**	1062	C-O (exocyclic)	1061	1052	1051	1062	1061	1061
**s**	2166		X	X	X	X	X	2169 (very weak)
**t**	1684		X	X	X	X	X	

**u**	1918		X	X	X	X	X	X
**v**	2347		2347	X	2347	X	X	

Supplementary Table 3: Average peak wave numbers of 1000 KDa lHA, HA/300-DETA-NO, HA/SNP, HA/SNAP, HA/SIN-1, HA/1000-H_2O_2 and HA/HAase and their approximate assignment. The main bands of untreated lyophilized HA (lHA) are denoted from **a** to **l**, and the new bands observed in the HA products are denoted from **m** to **v**.

**No.**	**Band (cm^{-1})**	**Approximate assignments**	**HA products**					
			**HA/300-DETA-NO**	**HA/SNP**	**HA/SNAP**	**HA/SIN-1**	**HA/1000-H_2O_2**	**HA/HAase**
**a**	3362	OH and NH stretching	Broad	3358	3249 (very broad)	3335	Very broad	3278
**b**	2874	CH stretching	2871	2970	2970	X	X	2876
**c**	1620	Amide I (C=O stretching)	1641	1622	1619	1616	1602	1646
**d**	1551	Amide II (N-H bending and	1543	X	1561 (weak)	1565	X	1569

**e**	1454	C-N stretching)	1457	X	X	1459	X	1450
**f**	1401	C-O stretching	1408	X	X	1405	1397	1398
**g**	1373		1374	1373	1372	1370	X	1363
**h**	1316	Amide III	X	1311	1312	1309	1313	1315
**i**	1154	Carbohydrate peaks (C-O, O-H and C-O-H deformation 1154: C-O-C (O-bridge) 1039: C-OH group 945: Asymmetrical out-of-phase ring vibration	1145	X	X	1164	1159	Or 1139?
**j**	1039		1042	1039	1035	1036	1038	Or 1053?
**k**	977		X	983	980	978	971	970
**l**	945		938	954	952	945	X	942
**m**	1514	may be NO_2-stretching	1525	X	X	X	X	
**n**	1787		1787	X	X	X	X	1768 (very weak)

**o**	1740	C=O stretching of new amide bond	1745	1739 (strong)	1738 (strong)	1744 (Very weak)	X	X
**p**	1224	C-N stretching	1224	1227	1227	1224	1234	X
**q**	1261		Yes	X	X	1262	X	1254
**r**	1062	C-O (exocyclic)	Yes	1068	X	X	1077	1053
**s**	2166		2169 (very weak)	X	X	X	2162	X
**t**	1684			X	X	X	X	X
**u**	1918		X	Yes	X	X	X	X
